# Macrostructural Evolution of the Mitogenome of Butterflies (Lepidoptera, Papilionoidea)

**DOI:** 10.3390/insects13040358

**Published:** 2022-04-06

**Authors:** Di Liu, Andrea Basso, Massimiliano Babbucci, Tomaso Patarnello, Enrico Negrisolo

**Affiliations:** 1 Department of Comparative Biomedicine and Food Science, University of Padova, Viale dell’Università 16, 35020 Legnaro, Italy; di.liu@studenti.unipd.it (D.L.); massimiliano.babbucci@unipd.it (M.B.); tomaso.patarnello@unipd.it (T.P.); 2 Istituto Zooprofilattico Sperimentale delle Venezie, Viale dell’Università 10, 35020 Legnaro, Italy; andrea.basso.1988@gmail.com

**Keywords:** Papilionoidea, butterflies, mitogenomics, mitochondrial structural evolution, new gene orders

## Abstract

**Simple Summary:**

Papilionoidea is a superfamily of Lepidoptera encompassing about 19,000 species. In the present work, we study the evolution of the structure of the mitogenome of these lepidopterans. The mechanisms generating the eight arrangements known for Papilionoidea were investigated analysing the movements of different mitochondrial genes. Five newly sequenced/assembled mitogenomes were included in our analysis involving more than 600 genomes. We provide new findings that help to understand the evolution of the gene orders MIQGO, IMQGO, 2S1GO, ES1GO and S1NGO in different butterflies. We demonstrate that the evolution of the 2S1GO in Lycaenidae followed a complicated pathway with multiple events of duplication and loss of *trnS1* and changes in anticodon. We describe two new gene orders 2FFGO and 4QGO for *Ampittia subvittatus* (Hesperiidae) and *Bhutanitis thaidina* (Papilionidae).

**Abstract:**

The mitogenome of the species belonging to the Papilionodea (Lepidoptera) is a double stranded circular molecule containing the 37 genes shared by Metazoa. Eight mitochondrial gene orders are known in the Papilionoidea. MIQGO is the plesiomorphic gene order for this superfamily, while other mitochondrial arrangements have a very limited distribution. 2S1GO gene order is an exception and is present in several Lycaenidae and one species of Hesperiidae. We studied the macrostructural changes generating the gene orders of butterflies by analysing a large data set (611 taxa) containing 5 new mitochondrial sequences/assemblies and 87 de novo annotated mitogenomes. Our analysis supports a possible origin of the intergenic spacer *trnQ*-*nad2*, characterising MIQGO, from *trnM*. We showed that the homoplasious gene order IMQGO, shared by butterflies, species of ants, beetles and aphids, evolved through different transformational pathways. We identify a complicated evolutionary scenario for 2S1GO in Lycaenidae, characterised by multiple events of duplication/loss and change in anticodon of *trnS1*. We show that the gene orders ES1GO and S1NGO originated through a tandem duplication random loss mechanism. We describe two novel gene orders. *Ampittia subvittatus* (Hesperiidae) exhibits the gene order 2FFGO, characterised by two copies of *trnF*, one located in the canonical position and a second placed in the opposite strand between *trnR* and *trnN*. *Bhutanitis thaidina* (Papilionidae) exhibits the gene order 4QGO, characterised by the quadruplication of *trnQ*.

## 1. Introduction

**Figure 1:**
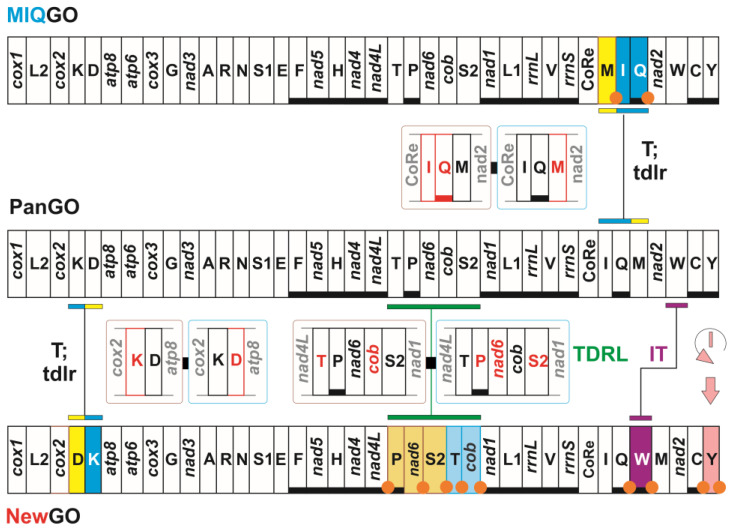
Mitochondrial macro-structural rearrangements. On top, transformational pathway from the gene order PanGO to the gene order MIQGO. On bottom, mechanisms generating a hypothetical new gene order. NewGO: hypothetical gene order generated by the rearrangements depicted in Figure 1. PanGO is linearized starting from *cox1*. The genes encoded on the plus strand (orientation from right to left in Figure 1) are black-boxed, while those encoded on the minus strand (orientation from left to right in Figure 1) are underlined and black-boxed. Nomenclature: *atp6* and *atp8*: ATP synthase subunits 6 and 8; cob: apocytochrome b; *cox1–3*: cytochrome c oxidase subunits 1–3; *nad1*–6 and *nad4* L: NADH dehydrogenase subunits 1–6 and 4 L; *rrnS* and *rrnL*: small and large subunit ribosomal RNA (rRNA) genes; X: transfer RNA (tRNA) genes, where X is the one-letter abbreviation of the corresponding amino acid, in particular L1 (CTN codon family) L2 (TTR codon family), S1 (AGN codon family) S2 (TCN codon family); CoRe: Control Region. I: inversion: IT: inverse transposition, T: transposition event. tdrl: tandem duplication random loss mechanism producing the observed rearrangement. TDRL, Tandem Duplication Random Loss move. Gene/genes, transposed relative to PanGO, is/are shown with a yellow/blue background. The extra copy of every gene that is lost in the genomic rearrangement is red-boxed. Genes located immediately upstream/downstream the rearranged genes, and, possibly, partly involved by the genomic change are grey-boxed. An orange dot marks an intergenic spacer present in a position associated with a genomic rearrangement.

The mitochondrial genome (hereafter mitogenome) of all species of Lepidoptera (Insecta) sequenced so far is a double stranded circular molecule spanning 15–18 Kilo-base pairs [1]. The lepidopteran mitogenome encodes the standard 37 genes of the Insecta and more generally of bilaterian Metazoa [1]. This set includes 13 protein-encoding genes, 22 tRNAs, 2 ribosomal rRNA (small and large subunits) and a control Region (Figure 1) [1].

The disposition of genes, i.e., the gene order, along the mitogenomic sequence is not fixed (Figure 1). This dynamic behaviour of the mitogenomic structure can generate multiple gene orders within the same clade (e.g., [1,2]). With respect to a reference gene order, novel/alternative gene orders are generated through events of transposition, inversion and inverse-transposition of one or multiple genes (Figure 1). The transposition of single genes/cluster of adjacent genes can be explained through a tandem duplication random loss model [3,4]. The inversion of a gene, which implies its repositioning on the opposite DNA filament, can be modelled through an intramitochondrial recombination mechanism [5]. The inverse-transposition can be due to a combination of the mechanisms described above [2].

More complicated structural rearrangements can involve large segments of the mitogenome (e.g., [2,6,7]). Papetti et al. [7] reviewed the many models developed for explaining these rearrangements. However, most of them do not apply to known gene orders for butterflies (see below). We cite here only the Tandem Duplication Random Loss move that allows explaining the simultaneous transposition of multiple non-adjacent genes (Figure 1) [8,9,10].

The 37 mitochondrial genes can be arranged in a huge number of gene orders (i.e., 37! = 1.367 × 1043 or 38! if the control region is also included), assuming that the movement of every gene is equally probable [1,2]. However, this scenario is unrealistic because the movements occur preferentially along specific pathways and some genes, especially the tRNAs, are much more mobile than others [1,2,6]. The reduction in the possible rearrangements increases the probability of convergent evolution in gene orders. Convergence can be limited to the sharing of local homoplastic gene-dispositions or involve the full gene order (e.g., [2,6,11]).

When a gene order becomes a molecular landmark defining a clade in a unique manner, it assumes the role of mitogenomic apomorphy. However, not every gene order is a mitochondrial apomorphy [2]. The degree of rearrangement of a gene order plays a key role for reaching this status. If the level of rearrangement is very high and involves multiple genes and large genomic segments, the probability that homoplasious identical gene orders appear in unrelated taxa is minimal/null [2]. Conversely, low levels of mitogenomic rearrangements are prone to generate fully homoplasious gene orders (e.g., [2,6,11]).

The clade, Pancrustacea, containing Insecta plus Crustacea, is characterized by a gene order (hereafter listed as PanGO) that sets them apart from all other animals [12,13]. PanGO is widespread in Insecta, but different gene orders originating from its rearrangements occur in various taxa (e.g., [1]). The number of alternative gene orders and their level of rearrangement have a very uneven distribution among insect orders [1,11].

Within Lepidoptera, PanGO is found only in a few moth families that branch off at the base of the lepidopteran phylogenetic tree [14,15]. Most of the lepidopteran mitogenomes sequenced to date exhibit a gene order (hereafter listed as MIQGO) characterised by a transposition of *trnM*, *trnI* and *trnQ* with respect to PanGO (Figure 1) (e.g., [15,16]). The formation of MIQGO can be explained through a tandem duplication random loss mechanism (Figure 1) [3,4,17].

MIQGO is shared by a very large clade including Ditrysia (the biggest lineage of Lepidoptera), Tischeriidae and the Australian Palaephatoidea [15]. MIQGO has been hypothesised to be a potential synapomorphy for the phyletic group exhibiting it [15]. However, some ants (Hymenoptera), some bush-crickets (Orthoptera) and a praying mantis (Mantodea) exhibit MIQGO in their mitogenomes [6,11,18,19]. Even if homoplasious, MIQGO characterises a large phyletic lineage within the Lepidoptera. However, its value as a clade-delimiting character and its range of applicability is determined within a phylogenetic contest [2]. Here, we introduce the concept of mito-signature. A mito-signature is a mitochondrial feature, and its possible modifications, which characterises all the taxa located downstream to a node in a phylogenetic tree. According to this definition, a mito-signature is not necessarily an apomorphy, as it happens for the MIQGO.

The superfamily Papilionoidea is a large clade of the Lepidoptera order encompassing about 19,000 species [20]. This superfamily contains butterflies belonging to the families Papilionidae, Pieridae, Nymphalidae, Lycaenidae and Riodinidae; the skippers (so named because they tend to skip from place to place with a quick flap of the wings) belonging to the family Hesperiidae and the moth-like species to the family Hedylidae [20,21,22,23,24,25].

**Figure 2:**
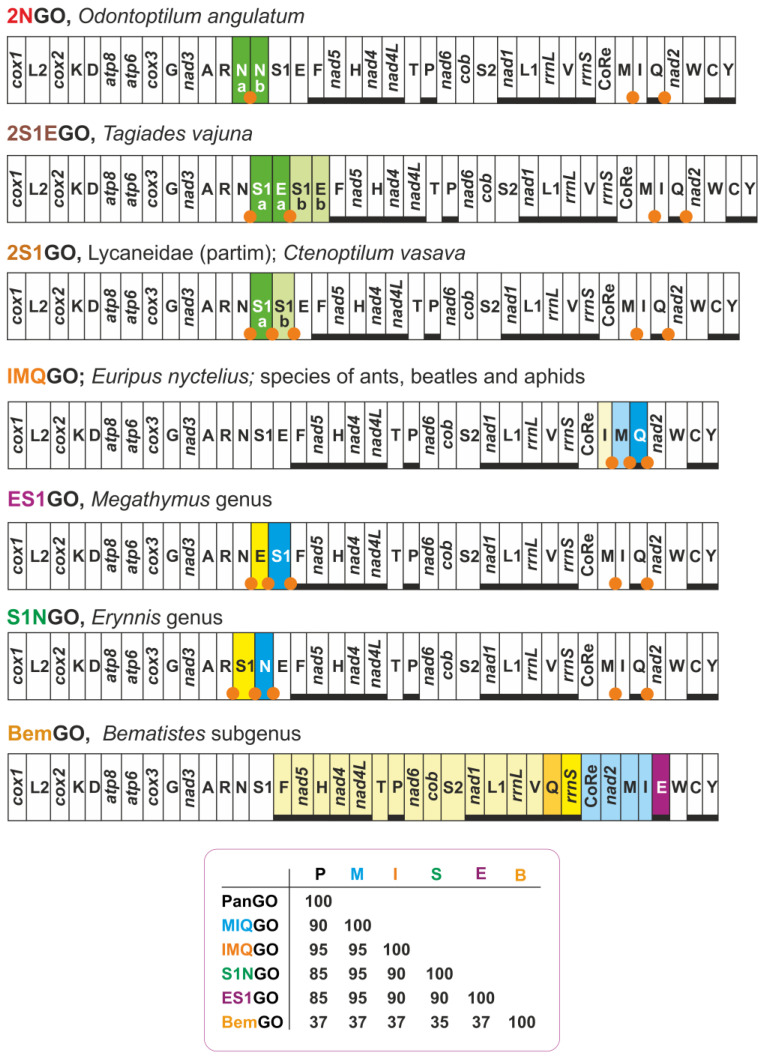
Alternative known gene orders and their distribution in butterflies. Genes characterizing each of genomic arrangements are presented with a coloured background. Green background, duplicated genes; yellow-orange/blue (different shades) background, transposed genes; purple background, gene that experienced and inverse-transposition. Nomenclature of genes as in Figure 1. On bottom, matrix-presenting scores (expressed in percentage) relative to shared common intervals among selected gene orders.

MIQGO characterises the mitogenomes of most of the butterflies sequenced to date (Figure 1), but some butterflies have different gene orders. Distribution of the alternative gene orders is limited to terminal and sub-terminal branches of the Papilionoidea phylogenetic tree (Figure 1 and Figure 2; Table 1).

We can divide the alternative gene orders into three types (Figure 2). Three gene orders with the same arrangement of MIQGO but presenting an extra copy of one or two adjacent tRNAs belong to the first type. The mitogenome of the skipper *Odontoptilum angulatum* exhibits a duplication of *trnN* (2NGO) [26]. Two contiguous copies of the *trnS1* and *trnE* occur in the mitogenome of the skipper *Tagiades vajuna* (2S1EGO) [27]. Finally, two copies of *trnS1* (2S1GO) appear independently in the mitogenomes of the skipper *Caenoptillum vasava* [28] and of several lycaenid butterflies (e.g., [29,30], present paper).

Three gene orders, characterised by a single event of transposition involving 2–3 contiguous tRNAs, form the second type. The arrangement IMQGO, where the *trnI* and *trnM* are transposed (IM vs. MI) with respect to MIQGO, is found in the mitogenome of the nymphalid *Euripus nyctelius* [31]. IMQGO is a homoplastic rearrangement as it also occurs in ants of the genus *Camponotus*, in curculionid beetles of the genus *Trypodendron*, and in the aphids of the family Pemphigidae [11,32,33]. A transposition of *trnS1* and *trnN* (S1N vs. NS1) characterises the arrangement S1NGO of the mitogenome of the skipper *Erynnis montanus* [34]. S1NGO is present in the mitogenome of other *Erynnis* species as described below. Finally, the giant-skippers of the *Megathymus* genus show an ES1GO arrangement, where *trnE* and *trnS1* are transposed (ES1 vs. S1E) with respect to MIQGO. The evolution of ES1GO is analysed in the present paper.

Only one gene order is known for being very different from MIQGO in Papilionidea. Within the genus *Acraea* (Nymphalidae, Heliconinae), the four species of the subgenus *Bematistes* sequenced to date have a mitogenome exhibiting a major structural rearrangement (BemGO) encompassing multiple genes [35] (Figure 2 and Figure S1). The complexity of the rearrangement occurring in the mitogenomes of *Bematistes* is evident provided that the reconstructed evolutionary pathway leading to this gene order implies the occurrence of the inverse-transposition of a gene (*trnE*) and two tandem duplication random loss moves (Figure S1). The peculiarity of BemGO is corroborated by the low percentage (37%) of shared common intervals with MIQGO (Figure 2). We will not analyse BemGO further in the present work, but we predict that it will prove to be a true synapomorphy for *Bematistes*.

Irrespective of the gene order, neighbour genes located on the same strand or opposite strands can be adjacent, overlapped or spaced by a variable number of nucleotides forming intergenic spacers (ISPs). DNA slippage during genome replication or mitogenomic rearrangements produce the ISPs [2,7,16]. The occurrence of a set of ISPs, congruent with the predicted evolutionary pathway leading to the formation of a specific gene order, is the first level of support for the proposed mitogenomic evolutionary scenario (e.g., [2,7]). The presence in the sequences of these ISPs of remnants of the genes involved in the rearrangement is more compelling evidence to confirm the transformational pathway generating a studied gene order (e.g., [2,7]).

Currently near complete/complete mitogenomes are available in the GenBank for more than 400 species of Papilionoidea and for some of them multiple sequences exist. This mitogenomic coverage is unmatched by any other lepidopteran Superfamily. Taking advantage of the large amount of data, we performed an in-depth analysis of the structural evolution of Papilionoidea mitogenome, both at a micro- and macro-structural level. We analysed more than 500 mitochondrial genomic sequences of Papilionoidea and about 140 outgroups were selected among the most closely related taxa [20]. We also sequenced the mitogenomes of two endangered butterflies, *Parnassius apollo* and *Lopinga achine*. Finally, we assembled the mitogenomes of the skippers *Erynnis brizo brizo*, *Gesta gesta* and *Ephyriades brunnea brunnea,* starting from available Illumina reads [36], as these taxa are key species for better understanding the evolution of S1NGO within the Hesperiidae [37] (see below).

In this paper, we present the results of our research focusing on the macro-structural evolution of Papilionoidea mitogenome. In particular, we present new findings on: (a) the evolution of the key ISP *trnQ*-*nad2* characterizing MIQGO, (b) the transformational pathways generating IMQGO, ES1GO and S1NGO, (c) the complicated evolutionary scenario behind the occurrence of 2S1GO in Lycaenidae, and (d) the discovery of two new gene orders 2FFGO and 4QGO, and the genomic mechanisms producing them.

## 2. Materials and Methods

### 2.1. Sequencing of Two New Mitogenomes

One specimen of *Parnassius apollo* (Linnaeus, 1758) (Voucher: FPa02. Collection data: Italy, Trento Province, Caoria, 46°11′41.39″ N 11°41′4.11″ E, 28–30 June 2008, collected by E. Negrisolo) and one specimen of *Lopinga achine* (Scopoli, 1763) (Voucher: FLa03. Collection data: Italy, Trento Province, Tonadico, Villa Welsperg, 29 June 2008, 46°11′54.81″ N 11°52′13.22″ E, collected by E. Negrisolo) were collected in the Natural Park Parco di Paneveggio—Pale di San Martino (Trentino Province, Italy). Collection of the specimens was carried out according to Italian national rules on protected species and with the permission of the Paneveggio—Pale San Martino Park. Total DNA was isolated using the ZR Genomic DNA-Tissue Midiprep (Zymo Research corp., Irvine, CA, USA) Kit. DNA quality was assessed through electrophoresis. The amplification and sequencing of both genomes were carried out through a combination of universal and species-specific designed primers [38,39]. The sequencing of purified PCR products was performed at the BMR Genomics service (Padova, Italy) on automated DNA sequencers mostly employing the primers used for PCR amplification. Chromatograms were visualised and corrected with the Chromas 2.5.1 software (Technelysium Ltd., City of Brisbane, Australia). The assembly of the whole genome sequences was performed with the SeqMan II program from the Lasergene software package (DNAStar, Madison, WI, USA). The two new mitogenomes are available in GenBank through the accession numbers ON087695 (*L. achine*) and ON087696 (*P. Apollo*).

### 2.2. Assembly of New Mitogenomes for Three Skippers (Hesperiidae)

The raw reads for *Erynnis brizo brizo* (GenBank accession number: SRR7174469), *Gesta gesta* (GenBank accession number: SRR7174466) and *Ephyriades brunnea brunnea* (GenBank accession number: SRR7174465) were downloaded from the European Nucleotide Archive (ENA) public database in FASTQ format. The software FastQC [40] was used to assess the quality of the reads and the presence of adapter sequences. We used SPAdes v-3.15.3 [41] to assemble, de novo, the complete genome (nuclear and mitochondrial scaffolds) of the three samples. SPAdes v-3.15.3 was run under the careful mode. This option runs MismatchCorrector, a post-processing tool that uses the BWA tool to correct the assemblies using the Illumina reads. The unique contig corresponding to the mitogenome (mtDNA) was identified by BLASTn [42] search against the gene sequences of the previously assembled and annotated mitogenome of *Erynnis montanus*.

### 2.3. Data Set Creation and Standardized Annotation of Mitogenomes

Initially, a starting data set including 511 near complete/complete mitochondrial genomes of Papilionoidea plus 138 complete mitogenomes of lepidopteran outgroups was created (Tables S1–S3). Outgroups were selected among the most closely related lineages to the superfamily Papilionoidea [20] in order to maximize taxonomic diversity at the genus level. In particular, all the superfamilies within the Apoditrysia clade, for which mitogenomes were available, were included in the starting data set. Outgroup species exhibiting the MIQGO were preferred over other gene orders because the bulk of mitogenomes of Papilionoidea exhibit this gene order and we wanted to make the comparisons of mitogenomic features as simple as possible.

The availability of new mitogenomes in the GenBank is a continuous process, occurring at an unpredictable pace. The partial/complete sequences of Papilionoidea available in the GenBank before 1 April 2021 were included in all analyses carried out for the present work. If available, multiple mitogenomes of the same butterfly species were included for investigating the intraspecific behaviour (Table S1). Some relevant sequences, available only later, were included only in selected analyses.

To ensure a standardised annotation of 649 mitogenomes forming the starting data set, all original annotations were checked and sometimes modified. We followed the well-established guidelines for a homogeneous annotation of mitogenomes (e.g., [6,16,43]). Several mitogenomes of butterflies are available in the GenBank as sequences without annotation. We produced new complete annotations for these sequences (Table S1).

To annotate/re-annotate the mitogenomes, we followed the strategy described in detail in previous open access works, carried out by our group [2,6,16,44]. Boundaries of every gene were further checked according to the guidelines provided by Cameron [43]. Transfer RNA genes were identified using the tRNAscan-SE program [45] or recognised manually as sequences with the appropriate anticodon and capable of folding into the typical cloverleaf secondary structure of tRNAs [46]. The validity of these predictions was further enhanced by comparison, based on multiple alignment and structural information, with published orthologous counterparts. The boundaries of the ribosomal *rrnL* and *rrnS* genes were determined by comparison to the orthologous counterparts present in the mtDNAs of the species already sequenced, as well as structural information implied by direct modelling (data not presented here). In this paper, the strand encoding the majority of genes is listed as plus-strand. Conversely, the strand encoding the minority of genes is listed here as minus strand.

After the process of checking and reannotation, 38 mitogenomes of Papilionoidea were excluded as it was impossible to correct evident errors in their sequence (Table S2). Thus, the final data set included 611 mitogenomes. Within this set, 138 sequences were from species belonging to outgroups (Table S3), while 473 mitogenomes belonged to 393 species of Papilionoidea (Table S1).

### 2.4. Sequence Heterogeneity Assessment

The GC-skew = (G − C)/(G + C) and AT-skew = (A − T)/(A + T) [47] were used to measure the base compositional differences among mitogenomes. The skews were calculated with Excel (Microsoft TM).

### 2.5. Phylogenetic Analyses

A phylogenetic analysis was performed on a data set limited to species belonging to Lycaneindae + Riodinidae (Table S1), because a reference tree was necessary to plot the evolution of 2S1GO (see below). Initially, each set of the 13 orthologous proteins, encoded in the mitogenome (Figure 1) was aligned with the MAFTT software [48,49]. Then, these alignments were concatenated in a single multiple alignment. The latter was analysed according to the maximum likelihood (ML) [50]. ML trees were computed with the program IQ-TREE 2.1.3 [51]. A phylogenomic partition model was used [52]. The optimal partitioning scheme in addition to the best fitting evolutionary model were selected with the ModelFinder method implemented in the program IQ-TREE 2.1.3 [53]. In the tree searching analyses, fifty independent runs were performed in order to avoid/minimize the possibility of becoming entrapped in sub-optimal trees. The ultrafast bootstrap test (UFBT) was performed to assess the statistical support of ML tree topologies (10,000 replicates) [54]. Finally, a neighbour-joining tree (NJ) was created through the analysis of the alignment of *trnS1* genes of Lycaenidae + Riodinidae. The NJ-tree was computed by applying the evolutionary model Kimura 2- parameter model [50] with the MEGA software [55]. The interior branch test (1000 replicates) was computed for the NJ-tree [56].

### 2.6. Macrostructural Evolution in the Mitogenome of Butterflies

Pairwise comparisons between different gene orders were performed with the CREx program [8]. This software analyses genomic rearrangement pathways using common intervals [8,9,10]. A common interval is a subset of genes that appear consecutively in two (or more) gene orders being investigated [8]. Initially, the number of shared common intervals (SCI) for each pair of gene orders was computed with CReX. The control region was included in the computation of values of shared common intervals. The computed values were transformed in percentages with Excel (Microsoft™). Percentages were used to evaluate the level of conservation among different gene orders [6]. Highly divergent gene orders share low percentages of shared common intervals (e.g., [2,6]).

The CREx program models rearrangements involving transpositions, inversions and inverse transpositions, in addition to the more complicated tandem duplications random loss moves [3,4,5,8,9,10]. In a tandem duplications random loss move, a tandem duplication of a continuous segment of genes occurs. Thus, the original segment and its copy are arranged consecutively. This duplication is followed by the loss of one copy of each redundant gene [10]. Multiple genes simultaneously change their position in a tandem duplications random loss move.

The online version of the ClustalW program available at the PRABI Rhone-Alpes Bioinformatics Centre NPS@ (http://www.prabi.fr/, accessed on 5 April 2022) was used to perform the alignments of ISPs and the genes involved in the mitogenomic rearrangements. The alignments were improved manually by visual inspection.

## 3. Results and Discussion

### 3.1. Mitogenomes: New Sequences, New Assemblies and New Annotations

We sequenced/assembled the complete mitogenomes of *Parnassius apollo* (Papilionidae), *Lopinga achine* (Nymphalidae), *Ephyriades brunnea brunnea* (Hesperiidae), and *Erynnis brizo brizo* (Hesperiidae), and the near complete genome of *Gesta gesta* (Hesperiidae). Size, AT-, GC- contents and skews are provided in Table S1. The five mitogenomes contained the canonical 37 genes of insect mitogenomes [1]. MIQGO characterises the mitogenome of *P. apollo*, *L. achine*, *E. brunnea brunnea* and *G. gesta*, while S1NGO occurs in the sequence of *E. brizo brizo* (see below). The five new genomes were included in the data set used in this paper to study the structural macroevolution of the mitogenome of butterflies. We annotated, de novo, 87 mitogenomes. Fifty-four sequences represented new species while the remaining sequences belonged to taxa with at least one mitogenome available (Table S1).

### 3.2. Some Statistics on Mitogenomes of Butterflies

The average size of the mitogenomes of butterflies was 15,303.35 ± 224.69 bases. The range of variation spanned from 14,964 bases (*Protesilaus protesilaus*, Papilionidae) to 17,733 (*Hesperia comma*, Hesperiidae). Mitogenomes were A + T-rich (%-average = 80.49% ± 0.95) with a range of variation spanning from 76.01% (*Acraea* (*Telchinia*) *polis*, Nymphalidae) to 83.13% (*Hesperia comma*, Hesperiidae). On the other hand, the G + C content (%-average = 19.48% ± 0.95) varied from 16.87% (*H. comma*) to 23.98% (*A. polis*). The mitogenomes exhibit AT-skews (AT-skew-average = −0.077 ± 0.018) ranging from −0.021 (*Cethosia cyane*, Nymphalidae) to 0.017 (*Hypolimnas bolina*, Nymphalidae). The GC-skews were always negative (GC-skew-average = −0.214 ± 0.024) and ranged from −0.326 (*Acraea* (*Bematistes*) *alcinoe*, Nymphlaidae) to −0.149 (*Dodona durga*, Riodinidae). All the species listed above exhibited the MIQGO arrangement with only exception represented by *A. alcinoe* (BemGO) (Figure 1 and Figure 2). Three of the four *Bematistes* butterflies exhibited the lowest GC-skews (Table S1). This result would suggest an impact of the BemGO genomic arrangement on the GC-skew. However, *Isoteinon lamprospilus*, a skipper with MIQGO, shows a GC-skew lower than the fourth *Bematistes* butterfly, i.e., *Acraea* (*Bematistes*) *poggei*. Very similar GC-skews characterised the mitogenomes of other butterflies presenting MIQGO (Table S1). These results suggest that the analysed different gene orders have a minimal/null effect on GC-skews. The homogenous behaviour of the statistics presented above also supports the view that no mitogenome experienced inversion of the control region, an event that is not immediately evident but can be detected through the computations of AT- GC-skews, which exhibit a change in the sign of their values if the control region is located on the opposite strand [57]. The statistics computed for the mitogenomes of outgroups mirrored those of butterflies, suggesting a homogenous behaviour within the analysed data set (Table S3).

### 3.3. The Origin of the Intergenic Spacer trnQ-nad2 in the Mitogenome of Butterflies

Mitogenomes of butterflies sequenced to date, exhibiting MIQGO, have an intergenic spacer located between *trnQ* and *nad2* (ISP *trnQ*-*nad2*) (Figure 1). This ISP was produced during the formation of MIQGO. Some authors think that the ISP *trnQ*-*nad2* is a remnant of a partly duplicated *nad2* that was generated during the rearrangement process [58,59,60]. Supporters of this hypothesis provided pairwise alignments of the ISPs *trnQ*-*nad2* and *nad2s* [58,59,60]. In these comparisons, the ISPs *trnQ*-*nad2* aligned with different portions (hereafter segments) of the *nad2* with percentage of identity for each pair varying from 62% to 78%. The search of these segments for the new mitogenomes of *P. apollo* and *L. achine* provided ambiguous results (Alignments S1–S2). In both species, at least two distinct *nad2* segments were identified with very similar percentages of identity (67.44% vs. 68.89%, *P. apollo*; 68.33% vs. 70.97%; *L. achine*), and further segments with percentages of identity >62% occurred in both butterflies. Because this behaviour could be peculiar for the new mitogenomes, the same search was repeated for species of Hesperiidae including all taxa analysed by Kim et al. [60]. Results were very similar to those obtained for of *P. apollo* and *L. achine* and multiple distinct segments of *nad2* were identified with very similar percentages of identity for each species (Alignments S3–S8). In some cases, the newly identified *nad2* segments exhibited higher percentages of identity than those identified by Kim et al. [60]. In *Choaspes benjaminii*, the segments B (percentage of identity = 78.08%) and E (percentage of identity = 78.08%) outperformed the D segment (percentage of identity = 69.32%) identified as the best match by Kim et al. [60] (Alignment S3). The same behaviour was observed for *Lobocla bifasciatus* and *Carterocephalus silvicola*. Thus, multiple distinct segments with very similar percentages of identity can be identified in each comparison (Alignments S4 and S7). The positioning of these segments along *nad2* is very variable, spanning most of the gene length.

A detailed analysis of the *nad2* segments vs. ISP *trnQ*-*nad2* alignments identified some common patterns (Alignments S1–S8). (a) The number of identical contiguous positions is low/very low and ranges from two to 13 (e.g., *C. benjaminii*, segment B), with 3–8 representing the more common condition. (b) Nucleotide composition of conserved positions is A/T-rich, in agreement with the compositional bias of butterflies’ mitogenomes. (c) The motifs of conserved nucleotides are, in many cases, not unique and have multiple counterparts scattered along the whole mitogenomic sequence.

If the *nad2* segment with the best percentage of identity is regarded as the true orthologous counterpart of the ISP *trnQ*-*nad2*, the following evolutionary scenario must be hypothesised.

First, the formation of MIQGO generated a near complete/complete second upstream copy of *nad2* that remained largely unchanged in the last common ancestor of Papilionoidea, and in the successive ancestors of families, subfamilies and so on. This behaviour is necessary because the ISPs *trnQ*-*nad2* align, according to the best percentage of identity scores, with very different segments, covering the whole length of *nad2*. Second, the shrinking of the pseudo-*nad2* leading to ISP *trnQ*-*nad2* must have occurred suddenly in the most recent ancestors of current species, because the length of these spacers is usually smaller than 100 nucleotides.

This evolutionary scenario is not plausible as it implies a very different behaviour of the lepidopteran mitogenome, i.e., a long period of stasis with the duplicated *nad2* transmitted through most of the branches of the tree, followed by very rapid shrinking in the sub-terminal/terminal nodes. Until now, there has been no evidence of this double behaviour in any studied animal mitogenome. The hypothesis of a long-lasting presence for a second near complete *nad2* is further contradicted by the fact that there is a strong selective pressure towards the maintenance of a compact mitogenome and constant gene content [7]. Finally, by assuming the origin of ISP *trnQ*-*nad2* from distinct segments of *nad2*, aligning them should not be possible as they represent the remnants of different non-orthologous segments of *nad2,* as identified through pairwise comparisons.

**Figure 3:**
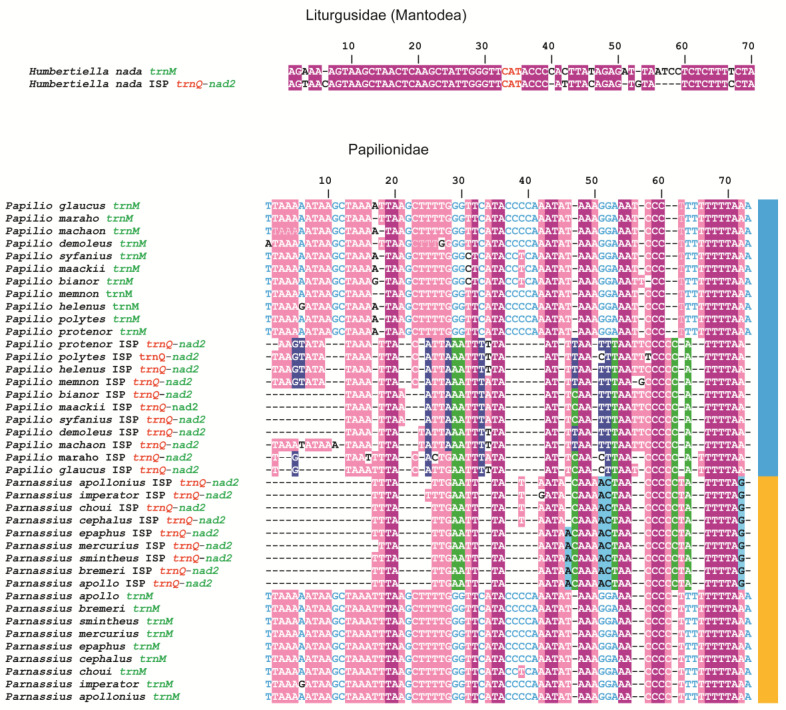
Alignments of *trnMs* vs. ISPs *trnQ*-*nad2*. On top, pairwise alignment of *trnM* vs. ISPs *trnQ*-*nad2* in *Humbertiella nada*. On bottom, multiple alignments of *trnMs* and ISPs *trnQ*-*nad2* of butterflies of the genera *Papilio* (Paapilioninae) and *Parnassius* (Parnassiinae). Deep-purple background, fully conserved nucleotide; pink background, majority rule (>50%) conserved nucleotide; cyan-coloured position, conserved nucleotide in *trnMs*; dark-blue background, conserved position in ISPs *trnQ*-*nad2* of *Papilio* species; light-blue background, conserved position in ISPs *trnQ*-*nad2* of *Parnassius* species; green-background conserved position in ISPs *trnQ*-*nad2* of *Papilio* + *Parnassius*.

The analysis of the mitogenome of the praying mantis *Humbertiella nada*, which shares with butterflies the homoplastic MIQGO, allowed us to hypothesise a different evolutionary scenario for the ISP *trnQ*-*nad2* origin. The ISP *trnQ*-*nad2* of *H. nada* contains in its sequence a pseudogene of *trnM* (Figure 3).

The ISP *trnQ*-*nad2* and *trnM* of *H. nada* share the identical segment AGTAAGCTAACTCAAGCTATTGGGTTCATACCC, spanning 33 nucleotides, including the anticodon CAT. The probability that an identical segment of this size appeared independently in ISP *trnQ*-*nad2* and *trnM* is null, as shown in other animal mitogenomes [7].

This finding prompted us to test the hypothesis that in lepidopteran mitogenomes, during the homoplastic genomic rearrangement that generated MIQGO, *trnM* also contributed to the formation of the ISP *trnQ*-*nad2* (Figure 1). To test this alternative evolutionary scenario, multiple alignments were created at different taxonomic levels containing the ISP *trnQ*-*nad2* and *trnM* sequences (Figure 3; Alignments S9–S12).

Before discussing the outputs of these alignments in detail, an explanation is necessary. The nucleotide substitution process is not limited by constraints in the sequences of ISP *trnQ*-*nad2*; conversely, the *trnMs* are under strong purifying selection. This opposite behaviour suggests that there should be a rapid/very rapid drop in the percentage of fully conserved positions when alignments are carried out at a taxonomic rank higher than genus. To mitigate this problem, we evaluated the pattern of conservation through a majority rule approach. Thus, we considered not only invariable positions but also positions conserved in more than 50% of the aligned sequences.

The analysis of the *trnMs* vs. ISPs *trnQ*-*nad2* alignments revealed some general features (Figure 3, Alignments S9–S12). (a) ISPs *trnQ*-*nad2* and *trnMs* can be aligned up to the level of families that are sister taxa (e.g., Lycaenidae and Riodinidae) (Alignment S10). We did not attempt a global alignment at a superfamily level. Within Papilionidae the level of conserved positions is 68.49% (50/73) (Figure 3). This alignment included butterflies of the genera *Parnassius* and *Papilio*, two taxa belonging to two distinct subfamilies that diverged around 59 MYA [61]. Within the Hesperiidae, the conserved positions account for the 55.67% (54/97) and the aligned sequences are derived from butterflies of different subfamilies that share a last common ancestor around 65 MYA old [61] (Alignment S9). Within the subfamily Pierinae, the analysed species share a last common ancestor dating back to 55.25 MYA [61] and the conserved positions represent the 64.47% (49/76) (Alignment S11). Within Lycaenidae and Riodinidae (Alignment S10), sharing a last common ancestor 97.36 MYA old [60], the conserved positions represent the 60.52% (46/76). Thus, the percentage of conserved positions is always above 50%. (b) ISPs *trnQ*-*nad2* and *trnMs* share conserved motifs in their alignments.

(c) The ISPs *trnQ*-*nad2* exhibit levels of conservation that make an origin from different mitochondrial segments implausible, as outlined in the examples listed below (Figure 3; Alignments S9–S12). Within Lycaenidae, the ISPs *trnQ*-*nad2* alignment exhibits 76.92% of conserved positions (50/65). Within Hesperiidae, the conserved positions account for 62.36% (58/93). Within Lycaenidae + Riodinidae, the conserved positions are 63.23% (43/68). Finally, hypothesising an origin from *trnM* is also a good fit to the actual size of most of ISPs *trnQ*-*nad2* and does not require very asymmetrical behaviour, i.e., a long stasis followed by a very rapid gene shrinking, necessary if *nad2* is considered the true ancestor of ISP *trnQ*-*nad2*.

Our findings exhibit some limitations. In contrast to what was found in the praying mantis *H. nada*, we did not identify within the alignments a unique DNA segment shared only by *trnMs* and ISPs *trnQ*-*nad2*. Furthermore, the sequence motifs shared by *trnMs* and ISPs *trnQ*-*nad2* are short, ranging from two to seven nucleotides, and not unique. The formation of the ISP *trnQ*-*nad2* is a very old event in the mitogenomes of Lepidoptera [15] and as mentioned above, this spacer does not have any structural constraint preventing it from changing quickly and not homogenously in the different lineages of butterflies.

Our results provide support to the origin of ISP *trnQ*-*nad2* from the *trnM*. However, this support is not undisputable, and alternative evolutionary scenarios can be put forward to explain the percentages of identical nucleotides shared by ISPs *trnQ*-*nad2* and *trnMs*. We believe that the *trnM* is a better candidate than *nad2* for explaining the origin of *ISP trnQ-nad2* in Lepidoptera. However, future analyses and more compelling evidence will be necessary to definitely prove or disprove this hypothesis.

### 3.4. Different Transformational Pathways Generate the Homoplastic IMQGO

**Figure 4:**
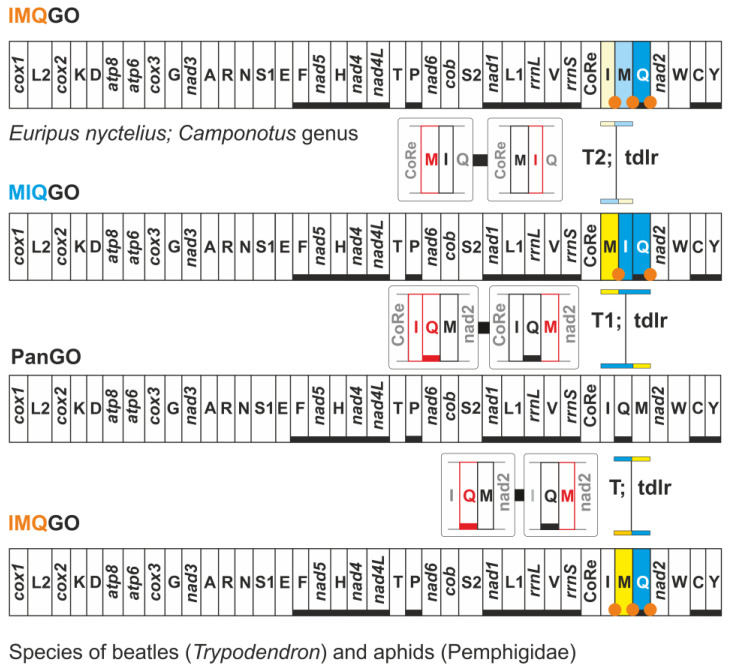
Mitochondrial transformational pathways generating IMQGO. Gene colours, nomenclature, as well mechanisms responsible for rearrangements depicted and labelled as in Figure 1.

As mentioned above, IMQGO is the homoplastic rearrangement shared by the nymphalid *E. nyctelius*, the ants of genus *Camponotus*, the beetles of the genus *Trypodendron* (Curculionidae), and the aphids of the family Pemphigidae. However, IMQGO appeared in these taxa following different transformational pathways (Figure 4).

Two events of transposition generated the final arrangement in *E. nyctelius* and the ants of the genus *Camponotus*. MIQGO is the plesiomorphic condition for both butterflies and ants [6,15]. Thus, there was not a direct transformational pathway going from PanGO to IMQGO for ants and lepidopterans. Conversely, a single event of transposition generated IMQGO in beetles and aphids (Figure 4). The analysis of the ISPs, associated with IMQGO, supports this single-step evolutionary scenario due to the presence of undisputed remnants of the copies of genes lost during the rearrangement (Figure 4; Alignments S13 and S14) [2,7]. Within the ISP, *trnQ*-*nad2* and ISP *trnM*-*trnQ* are present remnants of the copies of *trnM* and *trnI* that were lost in transposition. A remnant of the control region was identified in the ISP *trnM*-*trnQ* of *Trypodendron signatum* (Alignment S14). The presence of the control region remnant does is not surprising if we consider that any rearrangement process for the integrity of the moving genes must also involve portions of the genes located upstream/downstream to the genomic segment interested by the macro-structural change. Interestingly, IMQGO is structurally more similar to PanGO than MIQGO, as revealed by the percentages of shared common intervals (Figure 2).

### 3.5. The Evolution of 2S1GO within the Lycaenidae

**Figure 5:**
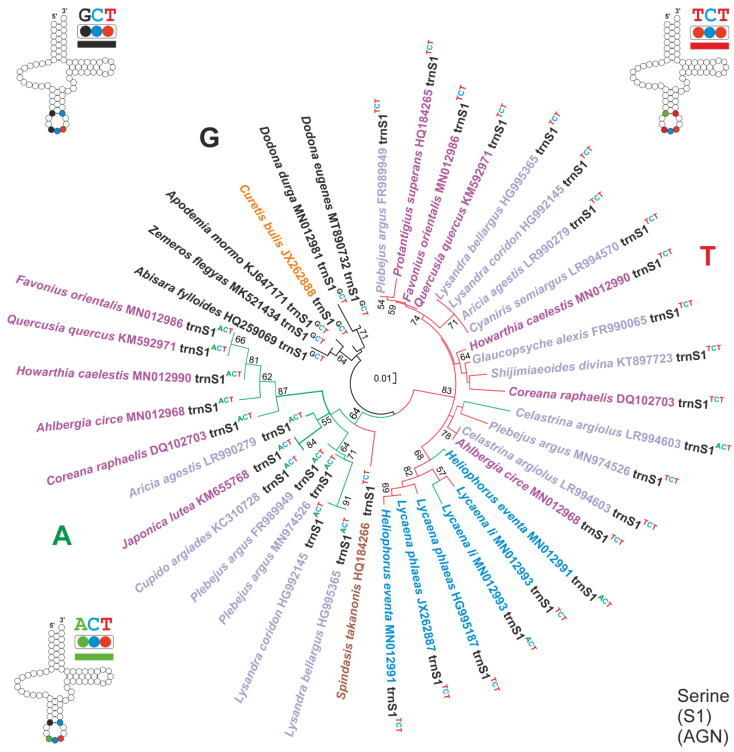
Phylogenetic relationships among *trnS1s* of Riodinidae + Lycaenidae. Neighbour-joining tree computed with MEGA. Number on branches are values, expressed in percentage, of the interior branch test. Secondary structures are provided for different types of *trnS1*. Names of species of Lycaenidae belonging to different subfamilies are coloured as follows: orange, Curetinae; cyan, Lycaenainae, light brown, Aphnaeinae; light-purple, Theclinae; light lilac, Polyommatinae.

Several lycaenid butterflies share with the skipper *C. vasava* the homoplastic 2S1GO (Figure 2), which implies the occurrence of two contiguous copies of *trnS1* (this paper) [28,29,30]. To study the evolution of 2S1GO in Lycaenidae, the multiple alignment of *trnS1s* belonging to the 611 taxa was generated. The alignment was carried out using the secondary structure determined for *trnS1* (Alignment S15) as a template. A phylogenetic tree was computed for the *trnS1s* of Riodinidae + Lycaenidae using the software MEGA (Figure 5).

**Figure 6:**
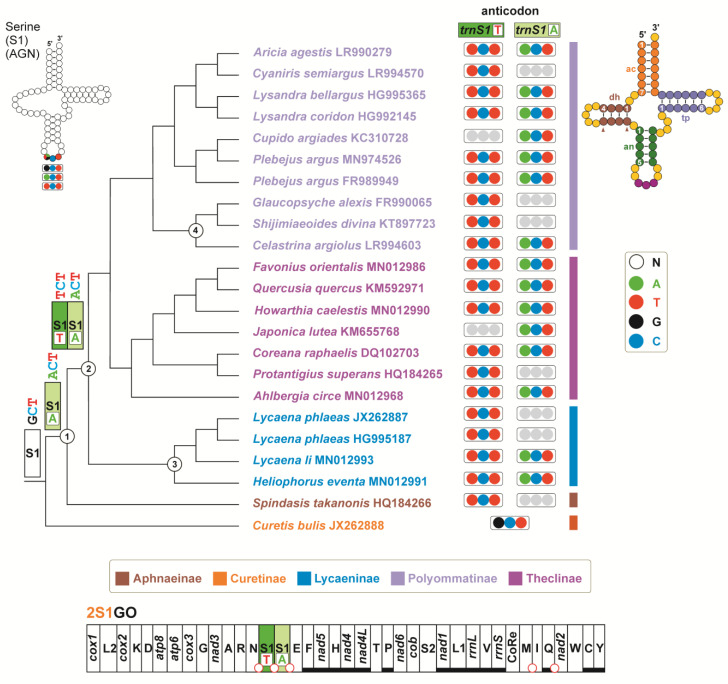
Evolution of *trnS1* in Lycaenidae. Tree, obtained with a ML analysis, depicting the phylogenetic relationships among analysed Lycaenidae. For reason of clarity, only the tree topology of the ingroup is provided. Outgroups (five species of Riodinidae) as well as UFBT node support (always 100%) are not presented. On top right, the secondary structure of *trnS1*: ac, acceptor stem; dh, DHU stem; an anticodon stem; tp, TΨC stem (nomenclature as in Montelli et al. [62]).

Finally, the distribution and the type of *trnS1* present in the different lycaenid butterflies was mapped in the ML reference phylogeny (Figure 6).

Outputs of these combined analyses revealed that the evolution of 2S1GO in Lycaenidae followed an unexpected, complicated pathway.

In Lepidoptera, the *trnS1* sequences exhibit three different anticodons, i.e., trnS1^GCT^, *trnS1^ACT^* and *trnS1^TCT^* (Alignment S15). The *trnS1^GCT^* is the most widespread and represents the plesiomorphic condition, while the alternative *trnS1^ACT^* and *trnS1^TCT^* appears independently through a process of parallel evolution in butterflies/moths belonging to different families (some examples are provided in Alignment S15).

Among the Lycaenidae sequenced so far, only *Curetis bulis* exhibits the *trnS1^GCT^* (Figure 5 and Figure 6). All other species have one *trnS1^ACT^*/*trnS1^TCT^* or both. When both *trnS1* types occur, they are always arranged in the 5′–3′ order *trnS1^TCT^*–*trnS1^ACT^* (Figure 6). The phylogenetic analysis performed on the *trnS1* of Lycaenidae + Riodinidae identified three major clusters: A, G and T (Figure 5). Only *trnS1^GCT^* sequences are included in cluster G. Only *trnS1^ACT^* sequences belong to cluster A. The *trnS1^TCT^* of *Spindasis takanonis* is a sister taxon of this cluster. Finally, cluster T includes all other lycaenid *trnS1^TCT^* sequences, and the *trnS1^ACT^* of *Lycaena li*, *Heliophorus eventa* and *Celastrina argiolus*. These three species have a second *trnS1^TCT^* located within the cluster T (Figure 5). Irrespective of the anticodon type, all *trnS1* sequences belonging to cluster T exhibit a fully compensatory base change in the an5 fifth base-pair of anticodon stem (i.e., G–C vs. A–T) (Figure 6; Alignment S15) [62]. This change is considered fully compensatory because it does not alter the secondary structure of *trnS1* [62]. Among the 611 sequences analysed here, which belong to 586 lepidopteran species, the same fully compensatory base change is present also in the skipper *Ochlodes venatus* (Hesperiidae) and in the moth *Eogystia hippophaecolus* (Cossidae), while all other taxa exhibit the plesiomorphic condition A-T. Interestingly, both *O. venatus* and *E. hippophaecolus* have a *trnS1^TCT^* sequence (Alignment S15).

Combining the phylogenetic output with the structural features exhibited by the *trnS1* sequences and the distribution of different anticodon types in lycaenid butterflies, an evolutionary scenario was reconstructed and is described in the steps (A–G) detailed below. Some parts of this transformational pathway are well corroborated, while others are tentatively sketched (Figure 6).

(A) The common ancestor of Lycaenidae had a single *trnS1^GCT^* in its mitogenome, a condition that is still present in *C. bulis*, which belong to the Curetinae, the sister group of all other Lycaenidae [23]. The *trnS1^GCT^* of *C. bulis* also shows the plesiomorphic condition for all the nucleotide pairs in the stems of the secondary structure (Alignment S15).
(B) First, a shift from the *trnS1^GCT^* to *trnS1^ACT^*, favoured by the fact that it is a transition from G to A, occurred in the ancestor of the not-Curetinae Lycaenids (node 1, of Figure 6). Representative of this early change are the *trnS1s^ACT^* contained in cluster A, which exhibit mostly a plesiomorphic condition in their nucleotide sequences (Alignment S15).
(C) Successively, in the common ancestor of the Lycaeninae, Polyommatinae and Theclinae, a duplication of *trnS1^ACT^* occurred and was followed by a shift in the TCT anticodon in the upstream copy (node 2, Figure 6). In this ancestral *trnS1s^TCT^* the G-C fully compensatory change also appeared, characterizing the 5an-pair of the anticodon stem (Figure 6; Alignment S15). The G-C change, present in the 5an-pair, has an extremely limited distribution among the 586 lepidopteran species studied here (Tables S1 and S3), which are representative of quite a large taxonomic diversity within Lepidoptera [20]. Thus, we regard it as highly improbable that this type of change occurred independently multiple times in Lycaenidae.
(D) Within Polyommatinae and Theclinae, multiple independent losses interested both *trnS1^ACT^* and *trnS1s^TCT^*.
(E) Within the Lycaenainae (node 3, Figure 6), the ancestral *trnS1^ACT^* was lost, but successively *trnS1s^TCT^* was duplicated and one copy reverted to *trnS1^ACT^*. The latter is present in *Lycaena li* and *Heliophorus eventa*, while it was successively lost in *Lycaena phlaeas*. This transformational pathway is supported by the fact that (1), irrespective to the anticodon type, the *trnS1s* of Lycaenainae share and unique nucleotide pattern in their TΨC loop. (2) The Lycaenainae *trnS1s^ACT^* are nested within the T cluster and share the G-C pair in the an5 position of the anticodon stem with all the *trnS1s^TCT^* (Figure 5; Alignment S15).
(F) The ancestral *trnS1^ACT^* was lost in a subclade of Polyommatinae (node 4; Figure 6), but in *Celastrina argioulus* there occurred a duplication of *trnS1^TCT^* and successive transformation of the downstream copy in *trnS1^ACT^*.
(G) A shift from *trnS1^ACT^* to *trnS1s^TCT^* occurred in the branch leading to *Spindasis takanonis.* In favour of this reconstruction is that the *trnS1^TCT^* of *S. takanonis* is sister taxon to cluster A and retains the plesiomorphic A-T pair in its anticodon stem (Figure 5; Alignment S15).

The *trnS1^ACT^* of *Japonica lutea* (KM655768) is identical to the *trnS1^ACT^* of *Cupido argiades* (KC310728), which belongs to the different subfamily Polyommatinae. Furthermore, the *J. lutea trnS1^ACT^* (KM655768) lacks a very peculiar fully compensatory base change (A–T vs. T–A) in the tp1-pair of the TΨC stem, which is present in all other *trnS1s^ACT^* of Theclinae (Figure 6; Alignment S15). All this is very unusual and needs further corroboration.

The evolutionary scenario depicted above shows a very complex transformational pathway with repeated events of duplications and loss of the different types of *trnS1,* as well as changes between the ACT and TCT anticodons. Not all steps described above are fully settled and a more complete taxon sampling is necessary to obtain a well-defined complete picture. Irrespective of some level of uncertainty, what clearly emerges is that the Lycaenidae are unique among butterflies and, more in general, Lepidoptera, for the high plasticity of their *trnS1*. This plasticity was present in the common ancestor of the non-Curetinae lycaenids and remained in the descendants, as proved by the multiple changes described above. Finally, the presence of this high plasticity suggests that *trnS1s* must be excluded from phylogenetic analyses because there is no guarantee that the relationship of orthology is fulfilled even among sequences sharing the same anticodon.

### 3.6. Two Mito-Signatures for the Genera Erynnis and Megathymus

**Figure 7:**
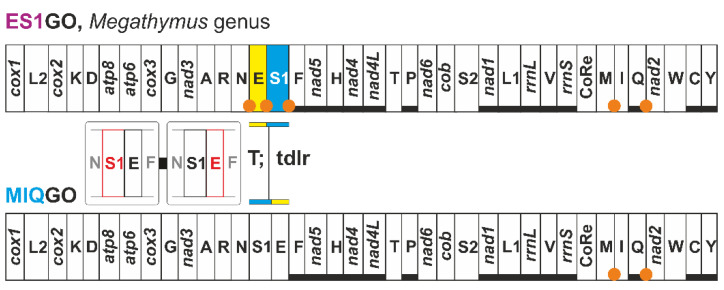
Mitochondrial transformational pathway generating ES1GO. Gene colours, nomenclature as well mechanisms responsible of rearrangements depicted and labelled as in Figure 1.

To our best knowledge, the evolution of ES1GO in the skippers of the *Megathymus* genus has never been studied in detail. S1NGO was unrecognised by the group that sequenced the *Megathymus* mitogenomes [63]. Indeed, these authors wrote that the sequences exhibit “… a gene order typical for mitogenomes of Lepidoptera”. The transformational pathway leading from MIQGO to ES1GO is shown in Figure 7.

A single tandem duplication random loss event can be assumed to be responsible for the appearance of this macro-structural change and a set of ISPs congruent with this transformational pathway is present in all the *Megathymus* mitogenomes. Reliable remnants of the genes involved in the tandem duplication random loss event, which would have better supported this transformational pathway [2,7], could not be found due to the rapid shortening and change in sequences associated with these intergenic spacers. Currently, ES1GO is known only for butterflies belonging to *Megathymus*, and its phylogenetic distribution represents a mito-signature for the genus. ES1GO does not show a highly rearranged gene disposition. Indeed, it shares 95% of shared common intervals with MIQGO (Figure 2). With current limited sampling, it is not possible to define unambiguously ES1GO as a true synapomorphy. Better genomic coverage is necessary to fix this point.

**Figure 8:**
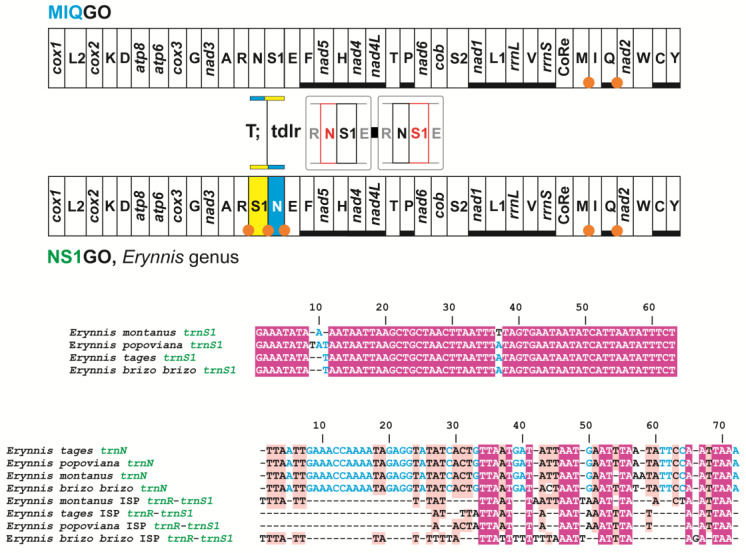
Mitochondrial transformational pathway generating NS1GO. Gene colours, nomenclature as well mechanisms responsible of rearrangements depicted and labelled as in Figure 1. On bottom, multiple alignments of *trnS1s* and *trnNs* vs. ISPs *trnR*-*trnS1* in species belonging to the genus *Erynnis*. Deep-purple background, fully conserved nucleotide; pink background, majority rule (>50%) conserved nucleotide; cyan-coloured position, conserved nucleotide in *trnMs*.

Initially, S1NGO was described for *Erynnis montanus* [34]. Our annotation/re-annotation and new assembly of the mitogenomes of *Erynnis tages*, *Erynnis popoviana* and *Erynnis brizo brizo* revealed that they share S1NGO with *E. montanus* (Figure 8).

The presence of S1NGO in the mitogenome of *E. popoviana* was unrecognized in the paper describing it [37]. However, the comparison of the *trnN* and *trnS1* sequences, identified during reannotation, with the orthologous counterparts of other *Erynnis* species provides compelling evidence in favour of this genomic arrangement (Figure 8). The occurrence of a single tandem duplication random loss event was hypothesised to explain the appearance of S1NGO [34]. All mitogenomes exhibiting S1NGO present ISPs in the segment interested by the tandem duplication random loss event (Figure 8). Furthermore, we identified possible remnants of the *trnNs* within the ISPs *trnR*-*trnS1*, even if in this case the evidence is not conclusive. All these findings corroborate the hypothesised tandem duplication random loss transformational pathway [2,7].

Currently, S1NGO is known only for the genus *Erynnis*. Indeed, *Gesta gesta* and *Ephyriades brunnea brunnea*, which resulted in the taxa with an available mitogenome, most closely related to the genus *Erynnis* in the very recent phylogeny of Xiao et al. [37], who presents the standard MIQGO. Available taxonomic coverage, combined with the low level of rearrangement of S1NGO that shares 95% of shared common intervals with MIQGO (Figure 2), suggests considering for the moment this gene order as a structural mito-signature, awaiting further corroboration to be possibly elevated to true synapomorphy.

### 3.7. FFGO and 4QGO: Two Novel Gene Orders for the Papilionoidea

Two novel gene orders were identified through the annotation of multiple mitogenomes, available as unannotated sequences in the GenBank (Table S1).

**Figure 9:**
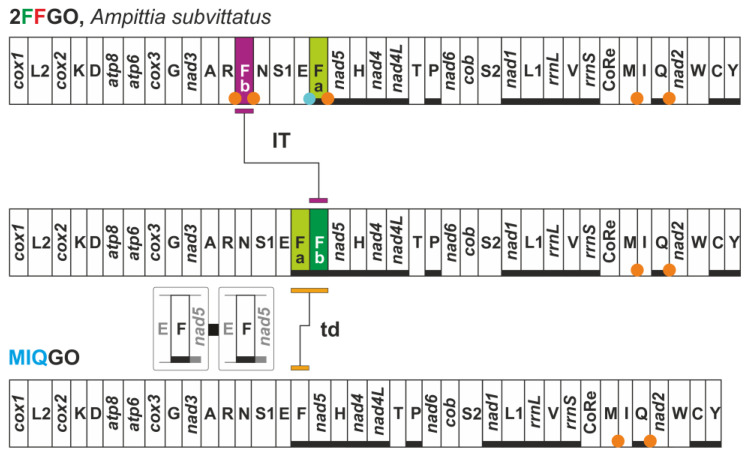
Mitochondrial transformational pathway generating 2FFGO. Gene colours, nomenclature as well mechanisms responsible of rearrangements depicted and labelled as in Figure 1 and Figure 2.

The first new arrangement (2FFGO) characterises the mitogenome of the skipper *Ampittia subvittatus* (Hesperiidae, Hesperiinae) (Figure 9).

Currently, 2FFGO is known only for this species as the congeneric *Ampittia dioscorides* exhibits the standard MIQGO. 2FFGO is characterised by the presence of two copies of *trnF*, located on opposite strands (Figure 9). Both *trnFa* (69 nucleotides) and *trnFb* (66 nucleotides) can perfectly fold and produce their secondary structure (Alignment S16). Their sequence identity is very high (92.28%), with most of the different nucleotides located in the TΨC loop (Alignment S16). The transformational pathway leading from MIQGO to 2FFGO implies the first duplication of *trnF,* followed by the reverse transposition of the downstream *trnFb* copy to a final placement between *trnR* and *trnS1*. It could be argued that after duplication took place, it was *trnFa* and not *trnFb* that moved between *trnR* and *trnS1*. The *trnE* and *trnFa* of *A. subvittatus* overlap for two nucleotides, mirroring the behaviour observed for *trnE* and *trnF* of *A. dioscorides*. This shared disposition favours the assumption that the partial superposition of this pair of tRNAs is the plesiomorphic condition for the genus *Ampittia*. The overlap of the two genes makes it more complicated to hypothesise the inverse transposition of one of the two without affecting the structure of the other, something that is not observed in the mitogenome of *A. subvittatus*. Thus, we favour in our reconstruction the reverse transposition of *trnFb*, which was free to move without affecting any other adjacent gene.

**Figure 10:**
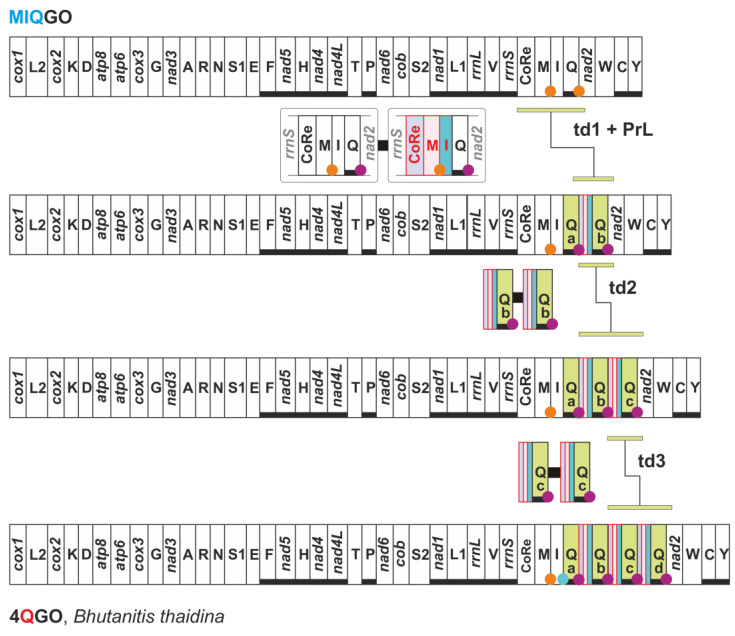
Mitochondrial transformational pathway generating 4QGO. Gene colours, nomenclature as well mechanisms responsible of rearrangements depicted and labelled as in Figure 1 and Figure 2. PrL, partial random loss; td, tandem duplication.

The mitogenome of the butterfly *Bhutanitis thaidina* (Papilionidae, Parnassiinae) presents a quadruplication of *trnQ* that generates the 4QGO arrangement (Figure 10).

This novel gene order does not occur in the congeneric *Bhutanitis mansfieldi* that shows the standard MIQGO (Table S1). To identify the transformational pathway leading to 4QGO, it is first necessary to analyse the intergenic spacers ISP *trnQa*-*trnQb*, ISP *trnQb*-*trnQc*, and ISP *trnQc*-*trnQd* (Figure 10; Alignment S17). These spacers share an identical sequence that can be divided into four segments: A–D (Alignment S17). The four segments unambiguously align with the ISP *trnQd*-*nad2* (A), a 3′ portion of control region (B); the 3′ end of *trnM* (C), and *trnI* (D). A second point to consider is that *trnI* and *trnQa* overlap for three nucleotides at their respective 3′ end, a condition they share with the *trnI* and *trnQ* pair of *B. mansfieldi*.

By combining all these data, we sketched the transformational pathway presented in Figure 10. The formation of 4QGO started with the duplication of a genomic segment spanning from the control region to *trnQ*. Successively, a partial random loss event coupled with the shortening of the DNA sequence occurred within the duplicated segment. This process produced the ISP *trnQa*-*trnQb* containing the four segments A-D. The next step was a duplication of the segment ISP *trnQa*-*trnQb* + *trnQb*, which generated the ISP *trnQb*-*trnQc* + *trnQc*. The last step was, again, a duplication of the segment the ISP *trnQb*-*trnQc* + *trnQc* that produced the final 4QGO. Alternatively, a second duplication of ISP *trnQa*-*trnQb* + *trnQb* would have created the same 4QGO arrangement. Multiple identical copies of a tRNA and the upstream spacer are not a common phenomenon in insect mitogenomes. However, *B. thaidina* is not the only example. Four identical copies of *trnL2* + upstream spacer occur in the mitogenome of the Eumenid wasp *Abispa ephippium* [64]. 2FFGO and 4QGO were identified in two non-annotated mitogenomic sequences available in the GenBank (Table S1). The sequencing of more mitogenomes of *A. dioscorides* and *B. thaidina* is desirable in the near future. This would provide independent corroboration to our findings.

### 3.8. Two Clusters of tRNAs Are the Hot Spots of Mitogenomic Diversity in Butterflies

With respect to PanGO, most of the gene orders present in butterflies (Figure 1, Figure 2, Figure 9 and Figure 10) involve duplication, transposition and inverse-transposition of one to three genes located within the two main clusters (ARNS1EF and MIQ) of tRNAs in the mitogenome of butterflies. BemGO represents the only exception (Figure 2 and Figure S1). Our results corroborate and extend previous findings that have identified the two hot spots of mitogenomic rearrangements in butterflies in these two clusters, and, more generally, in insects (e.g., [1]).

## Figures and Tables

**Table 1:** Gene orders, distribution in Papilionoidea and key features.

Name	Taxonomic Distribution	Key Feature
PanGO	Not present in Papilionoidea	apomorphic gene order for Pancrustacea
MIQGO	standard gene order for Papilionoidea	*trnM*, *trnI* and *trnQ* transposed with respect to PanGO
2NGO	*Odontoptilum angulatum* (Hesperiidae)	gene arrangement identical to MIQGO + 2 *trnN*
2S1EGO	*Tagiades vajuna* (Hesperiidae)	gene arrangement identical to MIQGO + 2 (*trnS1* + *trnE*)
2S1GO	*Caenoptillum vasava* (Hesperiidae), species of Lycaneinidae	gene arrangement identical to MIQGO + 2 *trnS1*
IMQGO	*Euripus nyctelius* (Nymphalidae)	*trnI*, *trnM* transposed (IM vs. MI) with respect to MIQGO
S1NGO	*Erynnis* genus (Hesperiidae)	*trnS1*, *trnN* transposed (S1N vs. NS1) with respect to MIQGO
ES1GO	*Megathymus* genus (Hesperiidae)	*trnE*, *trnS1* transposed (ES1 vs. S1E) with respect to MIQGO
BemGO	*Acraea* genus, subgenus *Bematistes* (Nymphalidae)	major structural rearrangement encompassing multiple genes with respect to MIQGO
2FFGO	*Ampittia subvittatus* (Hesperiidae)	two copies of *trnF* located on opposite strands
4QGO	*Bhutanitis thaidina* (Papilionidae)	gene arrangement identical to MIQGO + 4 *trnQ*

## Data Availability

Data are in the article and in the Supplementary Materials. The complete mitogenomes of *Lopinga achine* and *Parnassius apollo* are deposited in GenBank of NCBI under accession numbers ON087695 and ON087696.

## Supplementary Materials

The following supporting information can be downloaded at: https://www.mdpi.com/article/10.3390/insects13040358/s1, Figure S1: Mitochondrial transformational pathway generating BemGO; Table S1: Mitogenomes of Papilionoidea included in the final data set; Table S2: Mitogenomes of Papilionoidea excluded from the final data set; Table S3: Mitogenomes of Outgroups included in the final data set; Alignment S1: *nad2* vs. ISP *trnQ*-*nad2* pairwise alignments in *Lopinga achine* (Nymphalidae, Satyrinae); Alignment S2: *nad2* vs. *trnQ*-*nad2* ISP pairwise alignments in *Parnassius apollo* (Papilionidae, Parnassiinae); Alignment S3: *nad2* vs. ISP *trnQ*-*nad2* pairwise alignments in *Choaspes benjaminii* (KJ629164) (Hesperiidae, Coliadinae); Alignment S4: *nad2* vs. ISP *trnQ*-*nad2* pairwise alignments in *Lobocla bifasciatus* (KJ629166) (Hesperiidae, Eudaminae); Alignment S5: *nad2* vs. ISP *trnQ*-*nad2* pairwise alignments in *Euschemon rafflesia* (KY513288) (Hesperiidae, Euschemoninae); Alignment S6: *nad2* vs. ISP *trnQ*-*nad2* pairwise alignments in *Potanthus flavus* (KJ629167) (Hesperiidae, Hesperiinae); Alignment S7: *nad2* vs. ISP *trnQ*-*nad2* pairwise alignments in *Carterocephalus silvicola* (KJ629163) (Hesperiidae, Heteropterinae); Alignment S8: *nad2* vs. ISP *trnQ*-*nad2* pairwise alignments in *Daimio tethys* (KJ629165) (Hesperiidae, Tagiadinae); Alignment S9: Multiple alignment of *trnMs* and ISPs *trnQ*-*nad2* in selected species of the family Hesperiidae; Alignment S10: Multiple alignment of *trnMs* and ISPs *trnQ*-*nad2* in selected species of the families Lycaenidae and Riodinidae; Alignment S11: Multiple alignment of *trnMs* and ISPs *trnQ*-*nad2* in selected species of the subfamily Pierinae; Alignment S12: Multiple alignment of *trnMs* and ISPs *trnQ*-*nad2* in selected species of the subfamily Satyrinae (Nymphalidae); Alignment S13: *trnM* vs. ISP *trnQ*-*nad2* alignment and *trnI* vs. ISP *trnM*-trnQ alignment in species of the family Pemphigidae (Hemiptera); Alignment S14: *trnM* vs. ISP *trnQ*-*nad2* alignment and CoRe 3’p + *trnI* vs. ISP *trnM*-*trnQ* 5’p alignment in species of the family Curculionidae (Coleoptera); Alignment S15: Multiple alignment of *trnS1* sequences of selected species of Lepidoptera; Alignment S16: Multiple alignment of *trnF* sequences in Hesperiidae and pairwise alignment of *trnFa* and *trnFb* in *Ampittia subvittatus*; Alignment S17: Pairwise alignments of portions of ISP *trnQa*-*trnQb* with ISP *trnQd*-*nad2*, CoRe 3’-end, *trnM* and *trnI*; Pairwise alignment of ISPs *trnQ*-*nad2* in species of *Bhutanitis*.
