# Supplementary material for: Macrostructural Evolution of the Mitogenome of Butterflies (Lepidoptera, Papilionoidea)
Source: Insects. 2022 Apr 6;13(4):358. doi: 10.3390/insects13040358 (PMC9031222; doi:10.3390/insects13040358)
Supplement: Supplementary file 1 [file insects-13-00358-s001.zip › insects-1647722-supplementary materials-new.pdf]

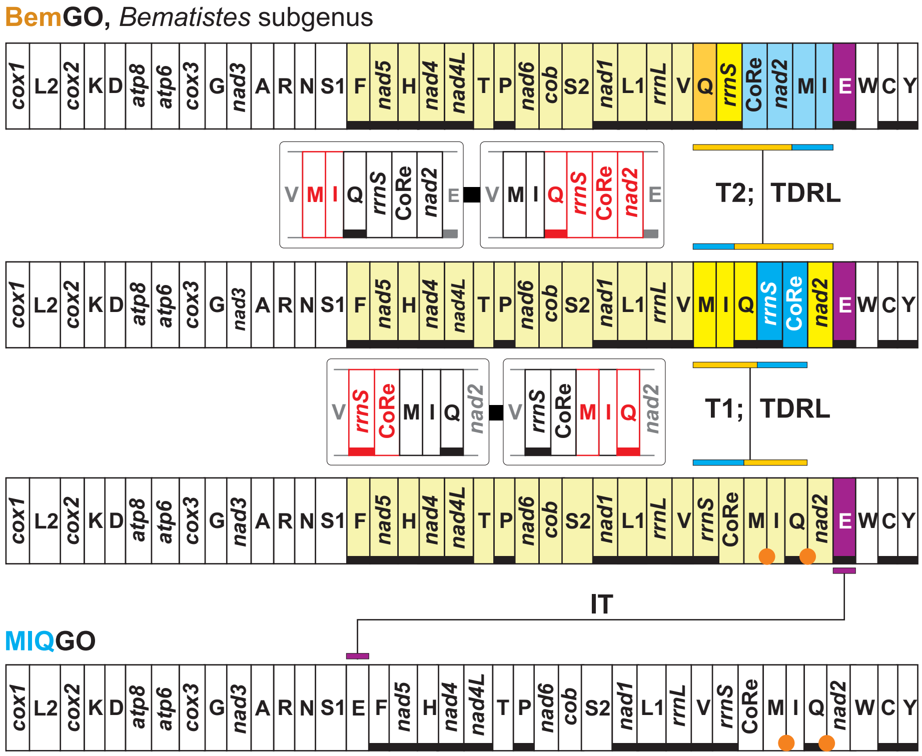

Figure S1. Mitochondrial transformational pathway generating **Bem**GO. **MIQ**GO and **Bem**GO are linearized starting from *cox1*. The genes encoded on the plus strand (orientation from right to left in Figure 1) are black-boxed, while those encoded on the minus strand (orientation from left to right in Figure 1) are underlined and black-boxed. Nomenclature: atp6 and atp8: ATP synthase subunits 6 and 8; cob: apocytochrome b; cox1-3: cytochrome c oxidase subunits 1–3; nad1-6 and nad4 L: NADH dehydrogenase subunits 1–6 and 4 L; rrnS and rrnL: small and large subunit ribosomal RNA (rRNA) genes; X: transfer RNA (tRNA) genes, where X is the one-letter abbreviation of the corresponding amino acid, in particular L1 (CTN codon family) L2 (TTR codon family), S1 (AGN codon family) S2 (TCN codon family); CoRe: Control Region. **IT**: inverse transposition, **T**: transposition event. **tdrl**: tandem duplication random loss mechanism producing the observed rearrangement. **TDRL**, **T**andem **D**uplication **R**andom **L**oss move. Genes transposed relative to **MIQ**GO are shown with a yellow-orange/blue (different shades) background. Genese that experienced an inverse-transposition are presented with purple bacground. Genes duplicated are presented with a green background. The extra copy of every gene that is lost in the genomic rearrangement is red-boxed. Genes located immediately upstream/downstream the rearranged genes, and, possibly, partly involved by the genomic change are grey-boxed. An orange dot marks an intergenic spacer present in a position associated to a genomic rearrangement.

Table S1. Mitogenomes of Papilionoidea included in the final data set

GO	SUPERFAMILY	FAMILY	SUBFAMILY	SPECIES	GenBank	C/P	Size	AT-skew	AT%	GC-skew	GC%	NOTE
MIQGO	Papilionoidea	Hedylidae		*Macrosoma conifera* (Warren, 1897)	MT852025	C	15344	-0,008	81,680	-0,185	18,320	
MIQGO	Papilionoidea	Hesperiidae	Barcinae	*Apostictopterus fuliginosus* Leech, [1893]	MH985707	C	15417	-0,006	80,736	-0,227	19,264	
MIQGO	Papilionoidea	Hesperiidae	Barcinae	*Barca bicolor* (Oberthür, 1896)	MN012971	C	15577	0,007	79,444	-0,247	20,556	new annotation
MIQGO	Papilionoidea	Hesperiidae	Barcinae	*Barca bicolor* (Oberthür, 1896)	MH985708	C	15574	0,007	79,382	-0,249	20,611	
MIQGO	Papilionoidea	Hesperiidae	Coeliadinae	*Burara striata* (Hewitson, 1867)	KY524446	C	15327	-0,020	80,127	-0,233	19,873	
MIQGO	Papilionoidea	Hesperiidae	Coeliadinae	*Choaspes benjaminii* (Guérin-Méneville, 1843)	JX101620	C	15272	-0,008	80,782	-0,218	19,218	
MIQGO	Papilionoidea	Hesperiidae	Coeliadinae	*Choaspes benjaminii* (Guérin-Méneville, 1843)	KJ629164	C	15300	0,002	80,111	-0,240	19,889	
MIQGO	Papilionoidea	Hesperiidae	Coeliadinae	*Hasora anura* (de Niceville, 1889)	KF881049	C	15280	0,005	79,503	-0,223	20,491	
MIQGO	Papilionoidea	Hesperiidae	Coeliadinae	*Hasora anura* (de Niceville, 1889)	KR189008	C	15290	0,001	79,529	-0,221	20,471	
MIQGO	Papilionoidea	Hesperiidae	Coeliadinae	*Hasora badra* (Moore, 1857)	MK238674	C	15324	-0,007	79,718	-0,229	20,282	
MIQGO	Papilionoidea	Hesperiidae	Coeliadinae	*Hasora chromus* (Cramer, 1782)	MN182753	C	15413	-0,007	79,641	-0,222	20,359	
MIQGO	Papilionoidea	Hesperiidae	Coeliadinae	*Hasora vitta* (Butler, 1870)	KR076553	C	15282	-0,010	79,918	-0,222	20,082	
MIQGO	Papilionoidea	Hesperiidae	Coeliadinae	*Hasora vitta* (Butler, 1870)	MK238675	C	15290	-0,009	79,993	-0,220	20,007	
MIQGO	Papilionoidea	Hesperiidae	Eudaminae	*Achalarus lyciades* Geyer, 1832	CM009487	C	15612	-0,012	81,899	-0,190	18,101	
MIQGO	Papilionoidea	Hesperiidae	Eudaminae	*Achalarus lyciades* Geyer, 1832	KX249739	C	15612	-0,012	81,899	-0,190	18,101	
MIQGO	Papilionoidea	Hesperiidae	Eudaminae	*Lobocla bifasciata* (Bremer & Grey, 1853)	MN012996	C	15613	-0,025	81,349	-0,192	18,651	new annotation
MIQGO	Papilionoidea	Hesperiidae	Eudaminae	*Lobocla bifasciatus* (Bremer & Grey, 1853)	KJ629166	C	15366	-0,020	80,932	-0,212	19,068	
MIQGO	Papilionoidea	Hesperiidae	Euschemoninae	*Euschemon rafflesia* (Macleay, [1826])	KY513288	C	15447	-0,040	81,440	-0,205	18,560	
MIQGO	Papilionoidea	Hesperiidae	Hesperiinae	*Agathymus mariae* (W. Barnes & Benjamin, 1924)	KY630504	C	15342	-0,004	79,983	-0,247	19,874	
2FFGO	Papilionoidea	Hesperiidae	Hesperiinae	*Ampittia subvittatus* (Moore, 1878)	MN013003	P	15403	-0,015	80,283	-0,245	19,717	new annotation
MIQGO	Papilionoidea	Hesperiidae	Hesperiinae	*Astictopterus jama* Felder & Felder, 1860	MH763663	C	15430	-0,012	80,687	-0,238	19,313	
MIQGO	Papilionoidea	Hesperiidae	Hesperiinae	*Erionota torus* Evans, 1941	MW586888	P	15987	-0,023	81,228	-0,222	18,772	new annotation
MIQGO	Papilionoidea	Hesperiidae	Hesperiinae	*Hesperia comma* (Linnaeus, 1758)	FR990041	C	17733	-0,001	83,128	-0,180	16,872	new annotation
MIQGO	Papilionoidea	Hesperiidae	Hesperiinae	*Isoteinon lamprospilus* Felder & Felder, 1862	MH763664	C	15430	-0,001	78,568	-0,283	21,432	
MIQGO	Papilionoidea	Hesperiidae	Hesperiinae	*Lerema accius* (Smith, 1797)	KT598278	C	15338	-0,022	80,858	-0,209	19,142	
ES1GO	Papilionoidea	Hesperiidae	Hesperiinae	*Megathymus beulahae* Scudder, 1872	KY630505	P	15412	0,004	79,101	-0,256	20,406	
ES1GO	Papilionoidea	Hesperiidae	Hesperiinae	*Megathymus cofaqui cofaqui* (Strecker, 1876)	KY630503	P	15421	-0,012	80,274	-0,242	19,052	
ES1GO	Papilionoidea	Hesperiidae	Hesperiinae	*Megathymus streckeri* (Skinner, 1895)	KY630501	C	15507	-0,011	80,764	-0,230	18,804	
ES1GO	Papilionoidea	Hesperiidae	Hesperiinae	*Megathymus ursus violae* D. Stallings and Turner, 1956	KY630502	C	15396	-0,008	80,508	-0,246	19,492	
ES1GO	Papilionoidea	Hesperiidae	Hesperiinae	*Megathymus yuccae* (Boisduval & Leconte, 1837)	KY630500	C	15477	-0,003	80,474	-0,244	19,229	
MIQGO	Papilionoidea	Hesperiidae	Hesperiinae	*Notocrypta curvifascia* (Felder & Felder, 1862)	MH763665	C	15546	-0,024	80,747	-0,228	19,253	
MIQGO	Papilionoidea	Hesperiidae	Hesperiinae	*Ochlodes sylvanus* (Esper, 1777)	FR990152	C	17059	-0,009	82,889	-0,207	17,111	new annotation
MIQGO	Papilionoidea	Hesperiidae	Hesperiinae	*Ochlodes thibetana* (Oberthur, 1886)	MN013004	C	15773	-0,022	82,064	-0,203	17,936	new annotation
MIQGO	Papilionoidea	Hesperiidae	Hesperiinae	*Ochlodes venata* (Bremer & Grey, 1853)	HM243593	C	15622	-0,010	81,993	-0,183	18,007	
MIQGO	Papilionoidea	Hesperiidae	Hesperiinae	*Parnara guttata* (Bremer & Grey, 1852)	JX101619	C	15441	-0,010	80,636	-0,218	19,364	
MIQGO	Papilionoidea	Hesperiidae	Hesperiinae	*Pelopidas mathias* (Fabricius, 1798)	MW264491	C	15524	-0,009	80,901	-0,213	19,099	
MIQGO	Papilionoidea	Hesperiidae	Hesperiinae	*Polytremis jigongi* J.Q. Zhu , Z.B. Chen & L.Z. Li, 2012	KP765762	C	15353	-0,016	80,942	-0,204	19,058	
MIQGO	Papilionoidea	Hesperiidae	Hesperiinae	*Polytremis nascens (Leech, 1893)*	KM981865	C	15392	-0,012	80,353	-0,213	19,647	
MIQGO	Papilionoidea	Hesperiidae	Hesperiinae	*Potanthus flavus* (Murray, 1875)	KJ629167	C	15267	-0,017	80,651	-0,217	19,349	
MIQGO	Papilionoidea	Hesperiidae	Heteropterinae	*Carterocephalus alcina* Evans, 1939	MN012974	P	15747	0,004	81,330	-0,192	18,664	new annotation
MIQGO	Papilionoidea	Hesperiidae	Heteropterinae	*Carterocephalus silvicola* Meigen, 1829	KJ629163	C	15765	0,006	80,298	-0,205	19,702	
MIQGO	Papilionoidea	Hesperiidae	Heteropterinae	*Heteropterus morpheus* (Pallas, 1771)	KF881050	C	15750	-0,001	81,308	-0,190	18,679	
MIQGO	Papilionoidea	Hesperiidae	Heteropterinae	*Leptalina unicolor* Bremer & Grey, 1853	MK265705	C	15854	0,016	81,241	-0,213	18,759	
MIQGO	Papilionoidea	Hesperiidae	Malazinae	*Malaza carmides* (Hewitson, 1868)	MN919191	C	15380	0,005	79,870	-0,250	19,863	
MIQGO	Papilionoidea	Hesperiidae	Malazinae	*Malaza empyreus* (Mabille, 1878)	MN919190	C	16103	0,004	80,997	-0,229	19,003	
MIQGO	Papilionoidea	Hesperiidae	Malazinae	*Malaza fastuosus* (Mabille, 1884)	MK301537	C	15579	0,005	80,358	-0,235	19,391	
MIQGO	Papilionoidea	Hesperiidae	Pyrginae	*Ephyriades brunnea brunnea* (Herrich-Schäffer, 1865)	SRR7174465	C	15645	-0,008	80,262	-0,196	18,498	de novo assembly
S1NGO	Papilionoidea	Hesperiidae	Pyrginae	*Erynnis brizo brizo* (Boisduval & LeConte, [1837])	SRR7174469	C	15644	0,001	82,012	-0,178	17,988	de novo assembly
S1NGO	Papilionoidea	Hesperiidae	Pyrginae	*Erynnis montanus* (Bremer, 1861)	MN012984	C	15477	-0,002	81,747	-0,183	18,253	new annotation
S1NGO	Papilionoidea	Hesperiidae	Pyrginae	*Erynnis montanus* (Bremer, 1861)	KC659955	C	15530	-0,001	81,758	-0,180	18,242	
S1NGO	Papilionoidea	Hesperiidae	Pyrginae	*Erynnis popoviana* Nordmann, 1851	MZ221165	C	15559	-0,003	81,798	-0,168	18,202	new annotation
S1NGO	Papilionoidea	Hesperiidae	Pyrginae	*Erynnis tages* (Linnaeus, 1758)	LR990102	C	15507	-0,003	82,195	-0,178	17,805	new annotation
MIQGO	Papilionoidea	Hesperiidae	Pyrginae	*Gesta gesta* (Herrich-Schäffer, 1863)	SRR7174466	P	15393	-0,007	81,043	-0,190	18,957	de novo assembly
MIQGO	Papilionoidea	Hesperiidae	Pyrginae	*Pyrgus maculatus* (Bremer & Grey, 1853)	MN013011	C	15366	-0,005	80,620	-0,216	19,380	new annotation
MIQGO	Papilionoidea	Hesperiidae	Pyrginae	*Pyrgus maculatus* (Bremer & Grey, 1853)	KP689265	C	15346	-0,003	80,679	-0,216	19,321	
MIQGO	Papilionoidea	Hesperiidae	Pyrginae	*Pyrgus malvae* (Linnaeus, 1758)	BK013352	C	15380	-0,009	81,671	-0,190	18,329	
MIQGO	Papilionoidea	Hesperiidae	Tagiadinae	*Abraximorpha davidii* Mabille, 1876	MT371044	C	15469	-0,015	81,544	-0,211	18,456	
MIQGO	Papilionoidea	Hesperiidae	Tagiadinae	*Celaenorrhinus maculosa* (C. & R. Felder, [1867])	KF543077	C	15282	0,001	79,872	-0,230	20,128	
MIQGO	Papilionoidea	Hesperiidae	Tagiadinae	*Daimio tethys* (Ménétries, 1857)	KJ629165	C	15350	-0,003	79,277	-0,239	20,723	
MIQGO	Papilionoidea	Hesperiidae	Tagiadinae	*Daimio tethys* (Ménétries, 1857)	KJ813807	C	15341	-0,002	79,154	-0,244	20,846	
2S1EGO	Papilionoidea	Hesperiidae	Tagiadinae	*Tagiades vajuna* Fruhstorfer,1910	KX865091	C	15359	-0,021	79,706	-0,220	20,294	
MIQGO	Papilionoidea	Hesperiidae	Trapezitinae	*Rachelia extrusus* C. & R. Felder, 1867	MN919192	C	16114	-0,016	80,681	-0,247	18,977	
MIQGO	Papilionoidea	Lycaenidae	Aphnaeinae	*Spindasis takanonis* (Matsumura, 1906)	HQ184266	C	15349	0,004	82,351	-0,214	17,649	
MIQGO	Papilionoidea	Lycaenidae	Curetinae	*Curetis bulis* Westwood, [1851]	JX262888	C	15162	-0,040	81,381	-0,175	18,619	
2S1GO	Papilionoidea	Lycaenidae	Lycaeninae	*Heliophorus eventa* Fruhstorfer 1918	MN012991	P	15531	0,005	80,175	-0,228	19,825	new annotation
2S1GO	Papilionoidea	Lycaenidae	Lycaeninae	*Lycaena li* (Oberthür, 1886)	MN012993	C	15420	-0,015	81,291	-0,197	18,696	new annotation
MIQGO*	Papilionoidea	Lycaenidae	Lycaeninae	*Lycaena phlaeas* (Linnaeus, 1761)	HG995187	C	16221	-0,028	82,819	-0,168	17,181	new annotation
MIQGO*	Papilionoidea	Lycaenidae	Lycaeninae	*Lycaena phlaeas* (Linnaeus, 1761)	JX262887	C	15280	-0,033	82,356	-0,165	17,644	
2S1GO	Papilionoidea	Lycaenidae	Polyommatinae	*Aricia agestis* (Denis & Schiffermüller, 1775)	LR990279	C	15467	-0,004	82,117	-0,174	17,883	new annotation
2S1GO	Papilionoidea	Lycaenidae	Polyommatinae	*Celastrina argiolus* (Linné, 1758)	LR994603	C	15337	-0,002	82,011	-0,166	17,989	new annotation
MIQGO*	Papilionoidea	Lycaenidae	Polyommatinae	*Cupido argiades* (Pallas, 1771)	KC310728	C	15330	-0,011	81,807	-0,167	18,193	
MIQGO*	Papilionoidea	Lycaenidae	Polyommatinae	*Cyaniris semiargus* (Rottemburg, 1775)	LR994570	C	15275	-0,007	81,663	-0,175	18,337	new annotation
MIQGO*	Papilionoidea	Lycaenidae	Polyommatinae	*Glaucopsyche alexis* Poda, 1761	FR990065	C	15228	-0,010	82,690	-0,161	17,310	new annotation
2S1GO	Papilionoidea	Lycaenidae	Polyommatinae	*Lysandra bellargus* (Rottemburg, 1775)	HG995365	C	15577	0,009	80,908	-0,190	19,092	new annotation
2S1GO	Papilionoidea	Lycaenidae	Polyommatinae	*Lysandra coridon* (Poda, 1761)	HG992145	C	15437	0,007	80,793	-0,197	19,207	new annotation
2S1GO	Papilionoidea	Lycaenidae	Polyommatinae	*Plebejus argus* (Linnaeus, 1758)	FR989949	C	15467	-0,008	81,748	-0,173	18,252	new annotation
2S1GO	Papilionoidea	Lycaenidae	Polyommatinae	*Plebejus argus* (Linnaeus, 1758)	MN974526	C	15426	-0,006	82,128	-0,168	17,872	
MIQGO*	Papilionoidea	Lycaenidae	Polyommatinae	*Shijimiaeoides divina* (Fixsen, 1887)	KT897723	C	15259	-0,005	82,450	-0,174	17,544	
2S1GO	Papilionoidea	Lycaenidae	Theclinae	*Ahlbergia circe* (Leech, 1893)	MN012968	C	15452	-0,040	82,701	-0,155	17,292	new annotation
2S1GO	Papilionoidea	Lycaenidae	Theclinae	*Coreana raphaelis* (Oberthür 1880)	DQ102703	C	15314	-0,047	82,663	-0,158	17,337	
2S1GO	Papilionoidea	Lycaenidae	Theclinae	*Favonius orientalis* (Murray, 1875)	MN012986	C	15390	-0,036	81,683	-0,179	18,317	new annotation
2S1GO	Papilionoidea	Lycaenidae	Theclinae	*Howarthia caelestis* Leech, 1890	MN012990	C	15383	-0,037	81,811	-0,180	18,189	new annotation
MIQGO*	Papilionoidea	Lycaenidae	Theclinae	*Japonica lutea* (Hewitson, 1865)	KM655768	C	15225	-0,043	82,010	-0,159	17,990	
MIQGO*	Papilionoidea	Lycaenidae	Theclinae	*Protantigius superans* (Oberthur, 1914)	HQ184265	C	15248	-0,036	81,683	-0,174	18,317	
2S1GO	Papilionoidea	Lycaenidae	Theclinae	*Quercusia quercus* (Linnaeus, 1758)	KM592971	P	15366	-0,040	81,674	-0,168	18,326	
MIQGO	Papilionoidea	Nymphalidae	Apaturinae	*Apatura laverna* (Leech, 1893)	MF444860	C	15187	-0,006	79,884	-0,216	20,116	
MIQGO	Papilionoidea	Nymphalidae	Apaturinae	*Apatura metis* Freyer, 1829	JF801742	C	15236	-0,012	80,441	-0,211	19,559	
MIQGO	Papilionoidea	Nymphalidae	Apaturinae	*Chitoria ulupi* (Doherty, 1889)	KP284554	C	15279	-0,026	79,809	-0,228	20,191	
IMQGO	Papilionoidea	Nymphalidae	Apaturinae	*Euripus nyctelius* (Doubleday, 1845)	KR020515	C	15417	-0,006	79,990	-0,222	20,010	
MIQGO	Papilionoidea	Nymphalidae	Apaturinae	*Herona marathus* (Doubleday, 1848)	KT279805	C	15487	-0,007	79,951	-0,248	20,049	
MIQGO	Papilionoidea	Nymphalidae	Apaturinae	*Hestina assimilis* (Linnaeus, 1758)	MN182752	C	15262	-0,013	81,136	-0,205	18,864	
MIQGO	Papilionoidea	Nymphalidae	Apaturinae	*Sasakia charonda* (Hewitson, 1863)	JX119051	C	15233	-0,007	79,905	-0,219	20,095	
MIQGO	Papilionoidea	Nymphalidae	Apaturinae	*Sasakia funebris* (Leech, 1891)	JX131328	C	15233	-0,017	81,232	-0,193	18,768	
MIQGO	Papilionoidea	Nymphalidae	Apaturinae	*Timelaea maculata* (Bremer & Grey, [1852])	KC572131	C	15178	-0,036	81,032	-0,196	18,968	
MIQGO	Papilionoidea	Nymphalidae	Biblidinae	*Ariadne ariadne* (Linnaeus, 1763)	MN012967	C	15149	-0,025	80,091	-0,226	19,909	new annotation
MIQGO	Papilionoidea	Nymphalidae	Biblidinae	*Ariadne ariadne* (Linnaeus, 1763)	KF990123	C	15179	-0,028	80,071	-0,216	19,929	
MIQGO	Papilionoidea	Nymphalidae	Biblidinae	*Hamadryas epinome* (Felder & Felder, 1867)	KM378244	C	15207	-0,031	81,502	-0,189	18,498	
MIQGO	Papilionoidea	Nymphalidae	Calinaginae	*Calinaga davidis* Oberthür, 1879	MN012976	C	15273	-0,045	80,613	-0,208	19,361	new annotation
MIQGO	Papilionoidea	Nymphalidae	Calinaginae	*Calinaga davidis* Oberthür, 1879	HQ658143	C	15267	-0,044	80,455	-0,201	19,545	
MIQGO	Papilionoidea	Nymphalidae	Charaxinae	*Polyura arja* C. Felder & R. Felder, 1867	KF590540	C	15363	-0,036	81,345	-0,188	18,655	
MIQGO	Papilionoidea	Nymphalidae	Charaxinae	*Polyura athamas* (Drury) 1773	MN013006	C	15404	-0,033	81,174	-0,196	18,826	
MIQGO	Papilionoidea	Nymphalidae	Charaxinae	*Polyura nepenthes* (Grose-Smith, 1883)	MN013013	C	15310	-0,033	80,745	-0,204	19,242	new annotation
MIQGO	Papilionoidea	Nymphalidae	Charaxinae	*Polyura nepenthes* (Grose-Smith, 1883)	KF990128	C	15333	-0,033	80,793	-0,199	19,207	
MIQGO	Papilionoidea	Nymphalidae	Cyrestinae	*Cyrestis thyodamas* Boisduval, 1836	MN012979	C	15249	-0,017	81,048	-0,188	18,952	new annotation
MIQGO	Papilionoidea	Nymphalidae	Cyrestinae	*Cyrestis thyodamas* Boisduval, 1836	KF990125	C	15254	-0,016	81,008	-0,188	18,992	
MIQGO	Papilionoidea	Nymphalidae	Cyrestinae	*Dichorragia nesimachus* (Doyere, 1840)	KF590541	C	15355	-0,029	81,420	-0,188	18,333	
MIQGO	Papilionoidea	Nymphalidae	Danainae	*Danaus chrysippus* (Linnaeus, 1758)	KF690637	C	15236	-0,031	80,369	-0,238	19,631	
MIQGO	Papilionoidea	Nymphalidae	Danainae	*Danaus genutia* (Cramer, [1779])	MN012982	C	15247	-0,040	81,229	-0,203	18,771	new annotation
MIQGO	Papilionoidea	Nymphalidae	Danainae	*Danaus gilippus* (Cramer, 1779)	LR743664	C	15237	-0,033	79,937	-0,234	20,056	new annotation
MIQGO	Papilionoidea	Nymphalidae	Danainae	*Danaus gilippus* (Cramer, 1779)	LR743665	P	14869	-0,030	79,595	-0,233	20,405	new annotation
MIQGO	Papilionoidea	Nymphalidae	Danainae	*Danaus plexippus* (Linnaeus, 1758)	KC836923	C	15314	-0,048	81,363	-0,205	18,637	

GO	SUPERFAMILY	FAMILY	SUBFAMILY	SPECIES	GenBank	C/P	Size	AT-skew	AT%	GC-skew	GC%	NOTE
MIQGO	Papilionoidea	Nymphalidae	Danainae	*Euploea core* (Cramer, 1780)	KF590546	C	15192	-0,045	81,734	-0,187	18,253	
MIQGO	Papilionoidea	Nymphalidae	Danainae	*Euploea midamus* (Linnaeus, 1758)	KJ866207	C	15187	-0,036	80,878	-0,213	19,122	
MIQGO	Papilionoidea	Nymphalidae	Danainae	*Euploea mulciber* (Cramer, 1777)	HQ378507	C	15166	-0,038	81,426	-0,209	18,574	
MIQGO	Papilionoidea	Nymphalidae	Danainae	*Idea leuconoe* Erichson, 1834	MN012994	C	15182	-0,050	80,740	-0,229	19,260	new annotation
MIQGO	Papilionoidea	Nymphalidae	Danainae	*Idea leuconoe* Erichson, 1834	KR815449	C	15278	-0,027	80,456	-0,232	19,538	
MIQGO	Papilionoidea	Nymphalidae	Danainae	*Ideopsis similis* (Linnaeus, 1758)	KJ476729	C	15200	-0,041	81,559	-0,210	18,434	
MIQGO	Papilionoidea	Nymphalidae	Danainae	*Parantica aglea* (Stoll, 1782)	MH388505	C	15219	-0,035	79,578	-0,241	20,422	
MIQGO	Papilionoidea	Nymphalidae	Danainae	*Parantica aglea aglea* (Stoll, 1782)	MN938921	C	15214	-0,035	79,578	-0,244	20,422	
MIQGO	Papilionoidea	Nymphalidae	Danainae	*Parantica sita* (Kollar, 1844)	KF590544	C	15211	-0,032	80,488	-0,234	19,512	
MIQGO	Papilionoidea	Nymphalidae	Danainae	*Parantica sita* (Kollar, 1844)	MG571524	C	15156	-0,035	80,496	-0,233	19,504	
MIQGO	Papilionoidea	Nymphalidae	Heliconiinae	*Acraea* (*Acraea*) *zetes* (Linnaeus, 1758)	KT371361	C	15228	-0,053	81,330	-0,190	18,670	
MIQGO	Papilionoidea	Nymphalidae	Heliconiinae	*Acraea* (*Alacria*) *parrhasia* (Fabricius, 1793)	KT371374	C	15261	-0,013	77,400	-0,255	22,600	
MIQGO	Papilionoidea	Nymphalidae	Heliconiinae	*Acraea* (*Alacria*) *penelope* Staudinger, 1896	KT371367	C	15271	-0,008	76,976	-0,269	23,024	
MIQGO	Papilionoidea	Nymphalidae	Heliconiinae	*Acraea* (*Alacria*) *perenna* Doubleday, 1847	KT371369	C	15315	-0,017	77,049	-0,220	22,951	
BemGO	Papilionoidea	Nymphalidae	Heliconiinae	*Acraea* (*Bematistes*) *alcinoe* C. Felder & R. Felder, 1865	KT371373	C	15729	0,011	77,125	-0,326	22,875	
BemGO	Papilionoidea	Nymphalidae	Heliconiinae	*Acraea* (*Bematistes*) *epaea* (Cramer, 1779)	KT371368	C	15712	0,004	79,213	-0,286	20,787	
BemGO	Papilionoidea	Nymphalidae	Heliconiinae	*Acraea* (*Bematistes*) *poggei* Dewitz, 1879	KT371366	C	15361	-0,011	80,001	-0,280	19,999	
BemGO	Papilionoidea	Nymphalidae	Heliconiinae	*Acraea* (*Bematistes*) *vestalis* C. Felder & R. Felder, 1865	KT371375	C	15350	-0,006	79,661	-0,303	20,339	
MIQGO	Papilionoidea	Nymphalidae	Heliconiinae	*Acraea* (*Rubraea*) *egina* (Cramer, 1775)	KT371359	C	15405	-0,042	80,221	-0,214	19,779	
MIQGO	Papilionoidea	Nymphalidae	Heliconiinae	*Acraea* (*Stephenia*) *rogersi* Hewitson, 1873	KT371365	C	15274	-0,035	80,778	-0,215	19,222	
MIQGO	Papilionoidea	Nymphalidae	Heliconiinae	*Acraea* (*Telchinia*) *acerata* Hewitson, 1874	KT371360	C	15281	-0,019	79,707	-0,223	20,293	
MIQGO	Papilionoidea	Nymphalidae	Heliconiinae	*Acraea* (*Telchinia*) *bonasia* (Fabricius, 1775)	KT371364	C	15290	-0,012	78,764	-0,215	21,236	
MIQGO	Papilionoidea	Nymphalidae	Heliconiinae	*Acraea* (*Telchinia*) *circeis* (Drury, 1782)	KT371362	C	15235	-0,013	77,217	-0,258	22,783	
MIQGO	Papilionoidea	Nymphalidae	Heliconiinae	*Acraea* (*Telchinia*) *jodutta* (Fabricius, 1793)	KT371358	C	15272	-0,022	78,195	-0,211	21,805	
MIQGO	Papilionoidea	Nymphalidae	Heliconiinae	*Acraea* (*Telchinia*) *kalinzu* Carpenter, 1936	KT371376	C	15260	-0,006	76,802	-0,261	23,198	
MIQGO	Papilionoidea	Nymphalidae	Heliconiinae	*Acraea* (*Telchinia*) *lycoa* Godart, 1819	KT371370	C	15190	-0,021	77,762	-0,216	22,238	
MIQGO	Papilionoidea	Nymphalidae	Heliconiinae	*Acraea* (*Telchinia*) *pharsalus* Ward, 1871	KT371363	C	15224	-0,023	79,020	-0,224	20,980	
MIQGO	Papilionoidea	Nymphalidae	Heliconiinae	*Acraea* (*Telchinia*) *polis* Pierre, 1999	KT371371	C	15254	-0,001	76,013	-0,233	23,981	
MIQGO	Papilionoidea	Nymphalidae	Heliconiinae	*Acraea* (*Telchinia*) *serena* (Fabricius, 1775)	KT371372	C	15258	-0,016	79,467	-0,205	20,533	
MIQGO	Papilionoidea	Nymphalidae	Heliconiinae	*Argynnis childreni* Gray, 1831	KF590547	C	15131	-0,021	80,041	-0,231	19,939	
MIQGO	Papilionoidea	Nymphalidae	Heliconiinae	*Argynnis hyperbius* (Linnaeus, 1763)	MN012969	C	15158	-0,025	80,782	-0,219	19,211	new annotation
MIQGO	Papilionoidea	Nymphalidae	Heliconiinae	*Argynnis hyperbius* (Linnaeus, 1763)	JF439070	C	15156	-0,025	80,813	-0,215	19,187	
MIQGO	Papilionoidea	Nymphalidae	Heliconiinae	*Argynnis paphia* (Linnaeus, 1758)	KM592975	P	15208	-0,033	81,003	-0,200	18,339	
MIQGO	Papilionoidea	Nymphalidae	Heliconiinae	*Boloria selene* (Schiffermuller, 1775)	HG998571	C	15156	-0,011	80,984	-0,228	19,016	new annotation
MIQGO	Papilionoidea	Nymphalidae	Heliconiinae	*Cethosia biblis* (Drury, 1773)	KF990124	C	15211	-0,067	79,837	-0,221	20,163	
MIQGO	Papilionoidea	Nymphalidae	Heliconiinae	*Cethosia biblis* (Drury, 1773)	KR066948	C	15286	-0,067	80,211	-0,200	19,776	
MIQGO	Papilionoidea	Nymphalidae	Heliconiinae	*Cethosia cyane* (Drury, 1773)	MN012975	C	15259	-0,077	80,123	-0,212	19,837	new annotation
MIQGO	Papilionoidea	Nymphalidae	Heliconiinae	*Damora sagana* (Doubleday, 1847)	KY971464	C	15151	-0,030	81,011	-0,202	18,989	
MIQGO	Papilionoidea	Nymphalidae	Heliconiinae	*Fabriciana adippe* (Schiffermuller, 1775)	FR990011	C	15118	-0,025	80,579	-0,223	19,421	new annotation
MIQGO	Papilionoidea	Nymphalidae	Heliconiinae	*Fabriciana nerippe* (Felder & Felder, 1862)	JF504707	C	15140	-0,027	80,905	-0,210	19,095	
MIQGO	Papilionoidea	Nymphalidae	Heliconiinae	*Heliconius cydno* (Doubleday 1847)	KM208636	C	15367	-0,035	81,363	-0,184	18,637	
MIQGO	Papilionoidea	Nymphalidae	Heliconiinae	*Heliconius hecale* (Fabricius, 1776)	KM068091	C	15338	-0,033	81,627	-0,188	18,373	
MIQGO	Papilionoidea	Nymphalidae	Heliconiinae	*Heliconius ismenius* Latreille, [1817]	KP294327	C	15346	-0,029	81,200	-0,200	18,800	
MIQGO	Papilionoidea	Nymphalidae	Heliconiinae	*Heliconius melpomene* (Linnaeus, 1758)	KP100653	C	15327	-0,037	81,660	-0,186	18,340	
MIQGO	Papilionoidea	Nymphalidae	Heliconiinae	*Heliconius pachinus* Salvin, 1871	KM014809	C	15369	-0,035	81,222	-0,182	18,778	
MIQGO	Papilionoidea	Nymphalidae	Heliconiinae	*Heliconius sara* (Fabricius, 1793)	KP281778	C	15372	-0,024	80,634	-0,196	19,366	
MIQGO	Papilionoidea	Nymphalidae	Heliconiinae	*Issoria eugenia* (Eversmann, 1847)	MK598743	C	15206	-0,032	81,501	-0,197	18,499	
MIQGO	Papilionoidea	Nymphalidae	Heliconiinae	*Issoria lathonia* (Linnaeus, 1758)	HM243590	C	15172	-0,031	81,169	-0,188	18,824	
MIQGO	Papilionoidea	Nymphalidae	Libytheinae	*Libythea celtis* (Laicharting, 1782)	HQ378508	C	15164	-0,017	81,205	-0,184	18,795	
MIQGO	Papilionoidea	Nymphalidae	Limenitidinae	*Abrota ganga* Moore, 1857	KF590536	C	15356	-0,048	81,206	-0,182	18,794	
MIQGO	Papilionoidea	Nymphalidae	Limenitidinae	*Adelpha bredowii* Geyer, 1837	MG747642	C	15187	-0,035	79,937	-0,207	20,063	
MIQGO	Papilionoidea	Nymphalidae	Limenitidinae	*Adelpha ethelda* (Hewitson, 1867)	MG747609	C	15350	-0,037	79,844	-0,203	20,130	
MIQGO	Papilionoidea	Nymphalidae	Limenitidinae	*Adelpha iphiclus* (Linnaeus, 1758)	MG747610	C	15263	-0,042	80,541	-0,180	19,459	
MIQGO	Papilionoidea	Nymphalidae	Limenitidinae	*Athyma asura* Moore, 1858	KF590542	C	15181	-0,033	81,082	-0,201	18,885	
MIQGO	Papilionoidea	Nymphalidae	Limenitidinae	*Athyma cama* Moore, 1858	KF590526	C	15269	-0,047	80,562	-0,198	19,425	
MIQGO	Papilionoidea	Nymphalidae	Limenitidinae	*Athyma disjuncta* Leech, 1890	MG747630	C	15198	-0,034	81,123	-0,209	18,877	
MIQGO	Papilionoidea	Nymphalidae	Limenitidinae	*Athyma fortuna* Leech, 1889	MG747635	C	15192	-0,037	80,299	-0,213	19,701	
MIQGO	Papilionoidea	Nymphalidae	Limenitidinae	*Athyma jina* Moore, 1858	MG747636	C	15202	-0,032	80,812	-0,219	19,169	
MIQGO	Papilionoidea	Nymphalidae	Limenitidinae	*Athyma kanwa* Moore, 1858	MG747619	C	15278	-0,048	80,848	-0,188	19,139	
MIQGO	Papilionoidea	Nymphalidae	Limenitidinae	*Athyma kasa* Moore, 1858	KF590524	C	15230	-0,041	80,289	-0,206	19,711	
MIQGO	Papilionoidea	Nymphalidae	Limenitidinae	*Athyma libnites* (Hewitson, 1859)	MG747643	C	15216	-0,044	80,724	-0,197	19,223	
MIQGO	Papilionoidea	Nymphalidae	Limenitidinae	*Athyma nefte* (Cramer, 1780)	MG747634	C	15438	-0,049	80,762	-0,189	19,161	
MIQGO	Papilionoidea	Nymphalidae	Limenitidinae	*Athyma opalina* (Kollar, 1844)	KF590551	C	15240	-0,026	80,138	-0,225	19,856	
MIQGO	Papilionoidea	Nymphalidae	Limenitidinae	*Athyma perius* (Linnaeus, 1758)	KF590528	C	15277	-0,037	79,636	-0,206	19,840	
MIQGO	Papilionoidea	Nymphalidae	Limenitidinae	*Athyma pravara* Moore, 1857	MG747623	C	15100	-0,049	80,974	-0,186	18,947	
MIQGO	Papilionoidea	Nymphalidae	Limenitidinae	*Athyma punctata* Leech, 1890	MG747626	C	15240	-0,044	80,131	-0,219	19,869	
MIQGO	Papilionoidea	Nymphalidae	Limenitidinae	*Athyma ranga* Moore, 1857	MG747640	C	15265	-0,039	80,832	-0,202	19,168	
MIQGO	Papilionoidea	Nymphalidae	Limenitidinae	*Athyma recurva* Leech, 1893	MG747627	C	15189	-0,033	80,980	-0,222	19,014	
MIQGO	Papilionoidea	Nymphalidae	Limenitidinae	*Athyma selenophora* (Kollar, 1844)	KF590525	C	15200	-0,035	81,309	-0,185	18,691	
MIQGO	Papilionoidea	Nymphalidae	Limenitidinae	*Athyma selenophora* (Kollar, 1844)	KF590529	C	15208	-0,035	81,293	-0,180	18,707	
MIQGO	Papilionoidea	Nymphalidae	Limenitidinae	*Athyma sulpitia* (Cramer, 1779)	JQ347260	C	15268	-0,047	81,949	-0,176	18,051	
MIQGO	Papilionoidea	Nymphalidae	Limenitidinae	*Auzakia danava* (Moore, [1858])	MG747639	C	15367	-0,057	80,640	-0,202	19,334	
MIQGO	Papilionoidea	Nymphalidae	Limenitidinae	*Bhagadatta austenia* (Moore, 1872)	KF590545	C	15615	-0,024	79,148	-0,228	20,781	
MIQGO	Papilionoidea	Nymphalidae	Limenitidinae	*Chalinga pratti* (Leech, 1890)	MG747613	C	15290	-0,047	81,550	-0,195	18,443	
MIQGO	Papilionoidea	Nymphalidae	Limenitidinae	*Dophla evelina* (Stoll, 1790)	KF590532	C	15320	-0,039	80,979	-0,211	19,014	
MIQGO	Papilionoidea	Nymphalidae	Limenitidinae	*Euthalia irrubescens* Grose-Smith, 1893	KF590527	C	15365	-0,042	81,028	-0,184	18,633	
MIQGO	Papilionoidea	Nymphalidae	Limenitidinae	*Lelecella limenitoides* Oberthür, 1890	MN922294	C	15203	-0,025	81,556	-0,184	18,444	
MIQGO	Papilionoidea	Nymphalidae	Limenitidinae	*Lexias dirtea* Fabricius, 1793	KF590531	C	15250	-0,046	81,357	-0,181	18,643	
MIQGO	Papilionoidea	Nymphalidae	Limenitidinae	*Limenitis amphyssa* Ménétriés, 1859	KY569555	C	15164	-0,036	80,975	-0,201	19,025	
MIQGO	Papilionoidea	Nymphalidae	Limenitidinae	*Limenitis archippus* (Cramer, 1776)	MG747621	C	15220	-0,034	80,926	-0,189	19,074	
MIQGO	Papilionoidea	Nymphalidae	Limenitidinae	*Limenitis arthemis* (Drury, 1773)	MG747622	C	15279	-0,027	80,136	-0,218	19,864	
MIQGO	Papilionoidea	Nymphalidae	Limenitidinae	*Limenitis camilla* (Linnaeus, 1764)	LR990255	C	15177	-0,041	80,694	-0,212	19,306	new annotation
MIQGO	Papilionoidea	Nymphalidae	Limenitidinae	*Limenitis camilla* (Linnaeus, 1764)	MG747618	C	15129	-0,041	80,646	-0,213	19,347	
MIQGO	Papilionoidea	Nymphalidae	Limenitidinae	*Limenitis ciocolatina* Poujade, 1885	MG747625	C	15214	-0,020	79,953	-0,241	20,047	
MIQGO	Papilionoidea	Nymphalidae	Limenitidinae	*Limenitis cleophas* Oberthür, 1893	MG747638	C	15228	-0,023	80,556	-0,239	19,444	
MIQGO	Papilionoidea	Nymphalidae	Limenitidinae	*Limenitis doerriesi* Staudinger, 1892	KY569552	C	15233	-0,043	81,133	-0,192	18,867	
MIQGO	Papilionoidea	Nymphalidae	Limenitidinae	*Limenitis doerriesi* Staudinger, 1892	MG747637	C	15176	-0,040	80,957	-0,209	19,037	
MIQGO	Papilionoidea	Nymphalidae	Limenitidinae	*Limenitis elwesi* Oberthür, 1884	MG747620	C	15214	-0,045	81,156	-0,200	18,838	
MIQGO	Papilionoidea	Nymphalidae	Limenitidinae	*Limenitis glorifica* Fruhstorfer, 1909	MG747615	C	15177	-0,040	80,886	-0,205	19,088	
MIQGO	Papilionoidea	Nymphalidae	Limenitidinae	*Limenitis helmanni* Lederer, 1853	KY290566	C	15178	-0,043	81,084	-0,193	18,916	
MIQGO	Papilionoidea	Nymphalidae	Limenitidinae	*Limenitis lorquini* (Boisduval, 1852)	MG747641	C	15228	-0,033	80,713	-0,203	19,287	
MIQGO	Papilionoidea	Nymphalidae	Limenitidinae	*Limenitis moltrechti* Kardakov, 1928	KY569554	C	15178	-0,036	80,814	-0,217	19,186	
MIQGO	Papilionoidea	Nymphalidae	Limenitidinae	*Limenitis moltrechti* Kardakov, 1928	MG747633	C	15192	-0,035	80,799	-0,217	19,194	
MIQGO	Papilionoidea	Nymphalidae	Limenitidinae	*Limenitis populi* (Linnaeus, 1758)	MG747632	C	15219	-0,039	80,932	-0,201	19,068	
MIQGO	Papilionoidea	Nymphalidae	Limenitidinae	*Limenitis reducta* Staudinger, 1901	MG747617	C	15175	-0,020	80,442	-0,232	19,539	
MIQGO	Papilionoidea	Nymphalidae	Limenitidinae	*Limenitis sydyi* Lederer, 1853	KY569553	C	15200	-0,038	81,184	-0,199	18,816	
MIQGO	Papilionoidea	Nymphalidae	Limenitidinae	*Limenitis sydyi* Lederer, 1853	KY593939	C	15209	-0,037	81,228	-0,201	18,772	
MIQGO	Papilionoidea	Nymphalidae	Limenitidinae	*Limenitis weidemeyerii* (W.H. Edwards, 1861)	MG747614	C	15199	-0,034	80,722	-0,197	19,278	
MIQGO	Papilionoidea	Nymphalidae	Limenitidinae	*Litinga cottini* (Oberthür, 1884)	MG747631	C	15205	-0,025	80,283	-0,226	19,711	
MIQGO	Papilionoidea	Nymphalidae	Limenitidinae	*Litinga mimica* (Poujade, 1885)	MG747628	C	15183	-0,031	80,696	-0,225	19,304	
MIQGO	Papilionoidea	Nymphalidae	Limenitidinae	*Moduza lysanias* (Hewitson, 1859)	MG747612	C	15238	-0,039	80,765	-0,192	19,235	
MIQGO	Papilionoidea	Nymphalidae	Limenitidinae	*Moduza procris* (Cramer, 1777)	KY593938	C	15195	-0,048	80,684	-0,197	19,316	
MIQGO	Papilionoidea	Nymphalidae	Limenitidinae	*Neptis alwina* Bremer & Grey, 1852	MG457128	C	15226	-0,041	81,197	-0,183	18,803	
MIQGO	Papilionoidea	Nymphalidae	Limenitidinae	*Neptis clinia* Moore, 1872	KM244664	C	15189	-0,031	80,071	-0,200	19,751	
MIQGO	Papilionoidea	Nymphalidae	Limenitidinae	*Neptis philyra* Ménétriés, 1858	KF590552	C	15164	-0,035	80,117	-0,197	19,843	
MIQGO	Papilionoidea	Nymphalidae	Limenitidinae	*Neptis soma* Linnaeus, 1758	KF590533	C	15130	-0,024	79,960	-0,214	20,033	
MIQGO	Papilionoidea	Nymphalidae	Limenitidinae	*Pandita sinope* Moore, 1858	KF590530	C	15257	-0,044	81,438	-0,186	18,562	
MIQGO	Papilionoidea	Nymphalidae	Limenitidinae	*Pantoporia hordonia* (Stoll, 1790)	KF590534	C	15603	-0,036	80,702	-0,198	19,298	
MIQGO	Papilionoidea	Nymphalidae	Limenitidinae	*Parasarpa albomaculata* (Leech, 1891)	MG747629	C	15272	-0,033	80,991	-0,215	19,009	
MIQGO	Papilionoidea	Nymphalidae	Limenitidinae	*Parasarpa dudu* (Doubleday, 1848)	KF590537	C	15236	-0,031	81,065	-0,214	18,929	
MIQGO	Papilionoidea	Nymphalidae	Limenitidinae	*Parasarpa zayla* (Doubleday, 1848)	MG747624	C	15269	-0,039	80,359	-0,222	19,641	
MIQGO	Papilionoidea	Nymphalidae	Limenitidinae	*Parthenos sylvia* (Cramer, [1776])	KF590550	C	15249	-0,039	81,074	-0,198	18,919	
MIQGO	Papilionoidea	Nymphalidae	Limenitidinae	*Patsuia sinensium* (Oberthür 1876)	KY593937	C	15192	-0,037	81,240	-0,204	18,760	

GO	SUPERFAMILY	FAMILY	SUBFAMILY	SPECIES	GenBank	C/P	Size	AT-skew	AT%	GC-skew	GC%	NOTE
MIQGO	Papilionoidea	Nymphalidae	Limenitidinae	*Phaedyma columella* (Cramer, [1780])	KY593936	C	15197	-0,031	80,312	-0,204	19,688	
MIQGO	Papilionoidea	Nymphalidae	Limenitidinae	*Sumalia zulema* (Doubleday, 1848)	MG747611	C	15297	-0,032	79,996	-0,237	20,004	
MIQGO	Papilionoidea	Nymphalidae	Limenitidinae	*Tacola eulimene* (Godart, 1824)	MG747616	C	15669	-0,037	78,269	-0,231	21,731	
MIQGO	Papilionoidea	Nymphalidae	Limenitidinae	*Tacola larymna* (Doubleday, [1848])	MG747644	C	15503	-0,045	79,978	-0,234	20,015	
MIQGO	Papilionoidea	Nymphalidae	Limenitidinae	*Tanaecia julii* Lesson, 1837	KF590548	C	15316	-0,043	81,209	-0,178	18,686	
MIQGO	Papilionoidea	Nymphalidae	Limenitidinae	*Tarattia gutama* Moore, 1858	MG747608	C	15266	-0,048	81,194	-0,177	18,748	
MIQGO	Papilionoidea	Nymphalidae	Nymphalinae	*Aglais ladakensis* (Moore, 1878)	MN732892	C	15222	-0,008	80,535	-0,221	19,465	
MIQGO	Papilionoidea	Nymphalidae	Nymphalinae	*Aglais urticae* (Linnaeus, 1758)	LR990014	C	15196	-0,006	80,482	-0,213	19,518	new annotation
MIQGO	Papilionoidea	Nymphalidae	Nymphalinae	*Anartia jatrophae saturata* Staudinger, 1885	MT712074	C	15297	-0,040	81,382	-0,192	18,618	
MIQGO	Papilionoidea	Nymphalidae	Nymphalinae	*Araschnia levana* (Linnaeus, 1758)	MT712075	C	15207	-0,009	81,647	-0,190	18,353	
MIQGO	Papilionoidea	Nymphalidae	Nymphalinae	*Baeotus beotus* (Doubleday, 1849)	MW566598	C	15131	-0,010	80,457	-0,215	19,543	
MIQGO	Papilionoidea	Nymphalidae	Nymphalinae	*Doleschallia melana* Staudinger, 1886	MT704829	C	15269	0,006	80,018	-0,237	19,982	
MIQGO	Papilionoidea	Nymphalidae	Nymphalinae	*Hypolimnas bolina* (Linnaeus, 1758)	KF990127	C	15260	0,018	79,672	-0,242	20,328	
MIQGO	Papilionoidea	Nymphalidae	Nymphalinae	*Inachis io* (Linnaeus, 1758)	LR989926	C	15369	-0,003	80,688	-0,227	19,312	new annotation
MIQGO	Papilionoidea	Nymphalidae	Nymphalinae	*Inachis io* (Linnaeus, 1758)	KM592970	C	15250	-0,004	80,525	-0,226	19,462	
MIQGO	Papilionoidea	Nymphalidae	Nymphalinae	*Junonia almana* (Linnaeus, 1758)	KF590539	C	15256	-0,010	80,257	-0,227	19,736	
MIQGO	Papilionoidea	Nymphalidae	Nymphalinae	*Junonia almana* (Linnaeus, 1758)	KX267580	C	15232	-0,010	80,213	-0,227	19,761	
MIQGO	Papilionoidea	Nymphalidae	Nymphalinae	*Junonia coenia grisea* Austin & J.F. Emmel, 1998	KX267578	C	15228	-0,017	80,667	-0,199	19,326	
MIQGO	Papilionoidea	Nymphalidae	Nymphalinae	*Junonia coenia* Hübner, [1822]	KT380025	C	15222	-0,018	80,699	-0,198	19,301	
MIQGO	Papilionoidea	Nymphalidae	Nymphalinae	*Junonia coenia* Hübner, [1822]	KX267572	C	15211	-0,016	79,456	-0,198	19,262	
MIQGO	Papilionoidea	Nymphalidae	Nymphalinae	*Junonia coenia* Hübner, [1822]	KX267579	C	15206	-0,018	80,633	-0,200	19,367	
MIQGO	Papilionoidea	Nymphalidae	Nymphalinae	*Junonia evarete flirtea* (Fabricius, 1793)	KX267569	C	15121	-0,017	80,597	-0,198	19,403	
MIQGO	Papilionoidea	Nymphalidae	Nymphalinae	*Junonia evarete zonalis* C. Felder & R. Felder, [1867]	KX267573	C	15240	-0,019	80,709	-0,197	19,291	
MIQGO	Papilionoidea	Nymphalidae	Nymphalinae	*Junonia genoveva hilaris* C. & R. Felder, [1867]	KX267570	C	15224	-0,016	80,748	-0,201	19,233	
MIQGO	Papilionoidea	Nymphalidae	Nymphalinae	*Junonia genoveva hilaris* C. & R. Felder, [1867]	KX267571	C	15238	-0,017	80,883	-0,204	19,117	
MIQGO	Papilionoidea	Nymphalidae	Nymphalinae	*Junonia iphita* (Cramer, 1779)	KU577289	P	14957	-0,001	79,902	-0,215	20,004	
MIQGO	Papilionoidea	Nymphalidae	Nymphalinae	*Junonia iphita* (Cramer, 1779)	KU577290	C	15190	-0,019	80,619	-0,198	19,335	
MIQGO	Papilionoidea	Nymphalidae	Nymphalinae	*Junonia lemonias* (Linnaeus, 1758)	KP941756	C	15230	-0,009	80,374	-0,224	19,606	
MIQGO	Papilionoidea	Nymphalidae	Nymphalinae	*Junonia orithya* (Linnaeus, 1758)	KF199862	C	15214	-0,016	80,380	-0,180	19,620	
MIQGO	Papilionoidea	Nymphalidae	Nymphalinae	*Junonia orithya madagascariensis* Guenée, 1865	KX267581	C	15231	-0,018	80,664	-0,199	19,322	
MIQGO	Papilionoidea	Nymphalidae	Nymphalinae	*Junonia rhadama* (Boisduval, 1833)	KX423732	P	15208	-0,017	79,964	-0,213	19,509	
MIQGO	Papilionoidea	Nymphalidae	Nymphalinae	*Junonia stygia* (Aurivillius, 1894)	MN623383	C	15233	-0,001	79,354	-0,248	20,442	
MIQGO	Papilionoidea	Nymphalidae	Nymphalinae	*Junonia vestina* C. & R. Felder, [1867]	KX267577	C	15224	-0,019	81,102	-0,193	18,891	
MIQGO	Papilionoidea	Nymphalidae	Nymphalinae	*Kallima inachus* (Doyère, 1840)	MN012995	C	15172	-0,013	80,616	-0,212	19,384	new annotation
MIQGO	Papilionoidea	Nymphalidae	Nymphalinae	*Kallima inachus* (Doyère, 1840)	HM243591	C	15150	-0,012	80,601	-0,213	19,399	
MIQGO	Papilionoidea	Nymphalidae	Nymphalinae	*Kallima inachus* (Doyère, 1840)	JN857943	C	15183	-0,013	80,300	-0,212	19,700	
MIQGO	Papilionoidea	Nymphalidae	Nymphalinae	*Kallima paralekta* (Horsfield, [1829])	MW192438	C	15200	-0,002	79,533	-0,241	20,467	
MIQGO	Papilionoidea	Nymphalidae	Nymphalinae	*Kallimoides rumia* (Westwood, 1850)	MT704827	C	15234	-0,007	80,898	-0,217	19,102	
MIQGO	Papilionoidea	Nymphalidae	Nymphalinae	*Mallika jacksoni* (Sharpe, 1896)	MT704828	C	15193	-0,015	79,385	-0,227	20,615	
MIQGO	Papilionoidea	Nymphalidae	Nymphalinae	*Melitaea athalia* (Rottemburg, 1775)	HG992208	C	15157	-0,020	80,326	-0,216	19,674	new annotation
MIQGO	Papilionoidea	Nymphalidae	Nymphalinae	*Melitaea cinxia* (Linnaeus, 1758)	HG992240	C	15173	0,006	79,826	-0,251	20,174	new annotation
MIQGO	Papilionoidea	Nymphalidae	Nymphalinae	*Melitaea cinxia* (Linnaeus, 1758)	CM002851	C	15171	0,004	79,810	-0,243	20,190	
MIQGO	Papilionoidea	Nymphalidae	Nymphalinae	*Melitaea cinxia* (Linnaeus, 1758)	GQ398377	C	15170	0,007	80,013	-0,245	19,987	
MIQGO	Papilionoidea	Nymphalidae	Nymphalinae	*Melitaea cinxia* (Linnaeus, 1758)	HM243592	C	15162	0,009	79,963	-0,247	20,037	
MIQGO	Papilionoidea	Nymphalidae	Nymphalinae	*Melitaea yuenty* Oberthür, 1886	MN013001	C	15149	0,006	79,695	-0,246	20,305	new annotation
MIQGO	Papilionoidea	Nymphalidae	Nymphalinae	*Mellicta ambigua* (Ménétriés, 1859)	MK252271	C	15205	-0,019	80,572	-0,219	19,428	
MIQGO	Papilionoidea	Nymphalidae	Nymphalinae	*Nymphalis polychloros* (Linnaeus, 1758)	HG992273	C	15249	0,007	79,730	-0,245	20,270	new annotation
MIQGO	Papilionoidea	Nymphalidae	Nymphalinae	*Polygonia c aureum* (Linnaeus, 1758)	MT654530	C	15202	-0,006	80,614	-0,231	19,386	new annotation
MIQGO	Papilionoidea	Nymphalidae	Nymphalinae	*Polygonia c aureum* (Linnaeus, 1758)	KX096653	C	15208	-0,006	80,635	-0,231	19,365	
MIQGO	Papilionoidea	Nymphalidae	Nymphalinae	*Polygonia c-aureum* (Linnaeus, 1758)	MF407452	C	15209	-0,006	80,604	-0,230	19,396	
MIQGO	Papilionoidea	Nymphalidae	Nymphalinae	*Protogoniomorpha anacardii duprei* (Vinson, 1863)	MT702382	C	15220	0,002	80,033	-0,233	19,967	
MIQGO	Papilionoidea	Nymphalidae	Nymphalinae	*Vanessa atalanta* (Linnaeus, 1758)	LR990581	C	15240	-0,001	80,400	-0,226	19,600	new annotation
MIQGO	Papilionoidea	Nymphalidae	Nymphalinae	*Vanessa cardui* (Linnaeus, 1758)	LR999956	C	15303	-0,022	81,036	-0,192	18,964	new annotation
MIQGO	Papilionoidea	Nymphalidae	Nymphalinae	*Vanessa indica* (Herbst, 1794)	MG736927	C	15191	0,002	80,074	-0,234	19,926	
MIQGO	Papilionoidea	Nymphalidae	Nymphalinae	*Yoma sabina* (Cramer, 1780)	KF590535	C	15191	0,002	80,074	-0,188	18,801	
MIQGO	Papilionoidea	Nymphalidae	Satyrinae	*Callerebia suroia* Tytler, 1914	KF906483	C	15208	-0,042	79,484	-0,223	20,516	
MIQGO	Papilionoidea	Nymphalidae	Satyrinae	*Coenonympha amaryllis* (Stoll, 1782)	MN756798	C	15125	-0,039	79,425	-0,226	20,575	
MIQGO	Papilionoidea	Nymphalidae	Satyrinae	*Davidina armandi* Oberthür, 1879	KF881046	C	15214	-0,023	79,696	-0,241	20,304	
MIQGO	Papilionoidea	Nymphalidae	Satyrinae	*Elymnias hypermnestra* (Linnaeus, 1763)	KF906484	C	15167	-0,044	80,458	-0,200	19,542	
MIQGO	Papilionoidea	Nymphalidae	Satyrinae	*Faunis aerope* (Leech, 1890)	MN012985	C	16381	-0,056	80,838	-0,214	19,150	new annotation
MIQGO	Papilionoidea	Nymphalidae	Satyrinae	*Hipparchia autonoe* (Esper, 1783)	MT090762	C	15300	-0,017	78,876	-0,239	21,124	
MIQGO	Papilionoidea	Nymphalidae	Satyrinae	*Hipparchia autonoe* (Esper, 1784)	GQ868707	C	15489	-0,016	79,088	-0,243	20,912	
MIQGO	Papilionoidea	Nymphalidae	Satyrinae	*Lasiommata deidamia* (Eversmann, 1851)	MG880214	C	15244	-0,034	81,120	-0,194	18,880	
MIQGO	Papilionoidea	Nymphalidae	Satyrinae	*Lasiommata majuscula* (Leech, 1892)	MN012997	C	15263	-0,038	81,629	-0,190	18,371	new annotation
MIQGO	Papilionoidea	Nymphalidae	Satyrinae	*Lethe albolineata* (Poujade, 1884)	KF881051	C	15248	-0,034	79,164	-0,240	20,836	
MIQGO	Papilionoidea	Nymphalidae	Satyrinae	*Lethe baileyi* South, 1913	MN611526	C	15225	-0,023	79,074	-0,238	20,926	
MIQGO	Papilionoidea	Nymphalidae	Satyrinae	*Lethe baucis* Leech, 1891	MN611527	C	15251	-0,031	78,736	-0,248	21,264	
MIQGO	Papilionoidea	Nymphalidae	Satyrinae	*Lethe confusa* Aurivillius, 1897	MT654529	P	14945	-0,056	80,422	-0,201	19,578	new annotation
MIQGO	Papilionoidea	Nymphalidae	Satyrinae	*Lethe dura* Marshall 1882	KF906485	C	15259	-0,035	79,265	-0,243	20,735	
MIQGO	Papilionoidea	Nymphalidae	Satyrinae	*Lethe hayashii* Koiwaya, 1993	MN611528	C	15246	-0,036	79,070	-0,235	20,930	
MIQGO	Papilionoidea	Nymphalidae	Satyrinae	*Lethe helle* Leech, 1891	MN611529	C	15253	-0,019	78,588	-0,258	21,412	
MIQGO	Papilionoidea	Nymphalidae	Satyrinae	*Lethe marginalis* Motschulsky, 1860	MN611530	C	15229	-0,042	80,189	-0,231	19,811	
MIQGO	Papilionoidea	Nymphalidae	Satyrinae	*Lethe nigrifascia* Leech, 1890	MN611531	C	15239	-0,023	79,316	-0,244	20,684	
MIQGO	Papilionoidea	Nymphalidae	Satyrinae	*Lethe oculatissima* (Poujade, 1885)	MN611532	C	15243	-0,037	79,276	-0,233	20,724	
MIQGO	Papilionoidea	Nymphalidae	Satyrinae	*Lethe satyrina* Butler, 1871	MN611533	C	15271	-0,041	78,764	-0,218	21,236	
MIQGO	Papilionoidea	Nymphalidae	Satyrinae	*Lethe syrcis* Hewitson, 1863	MN611534	C	15252	-0,038	79,144	-0,245	20,856	
MIQGO	Papilionoidea	Nymphalidae	Satyrinae	*Lethe titania* Leech, 1891	MN611535	C	15257	-0,028	78,469	-0,244	21,531	
MIQGO	Papilionoidea	Nymphalidae	Satyrinae	*Lethe uemurai* (Sugiyama, 1994)	MN611536	C	15272	-0,022	78,529	-0,250	21,471	
MIQGO	Papilionoidea	Nymphalidae	Satyrinae	*Lethe uemurai* (Sugiyama, 1994)	MN611537	C	15262	-0,022	78,522	-0,251	21,478	
MIQGO	Papilionoidea	Nymphalidae	Satyrinae	*Lethe verma* (Kollar, 1844)	MN611538	C	15239	-0,042	79,270	-0,227	20,730	
MIQGO	Papilionoidea	Nymphalidae	Satyrinae	*Lethe verma* (Kollar, 1844)	MN611539	C	15238	-0,041	79,275	-0,226	20,725	
MIQGO	Papilionoidea	Nymphalidae	Satyrinae	***Lopinga achine* (Scopoli, 1763)**	**ON087695**	C	15411	-0,028	79,781	-0,234	20,219	new mitogenome
MIQGO	Papilionoidea	Nymphalidae	Satyrinae	*Maniola jurtina* (Linnaeus, 1758)	HG995237	C	15256	-0,038	79,719	-0,234	20,281	new annotation
MIQGO	Papilionoidea	Nymphalidae	Satyrinae	*Melanargia asiatica* (Oberthür & Houlbert, 1922)	KF906486	C	15142	-0,036	79,045	-0,239	20,955	
MIQGO	Papilionoidea	Nymphalidae	Satyrinae	*Melanargia caoi* Lang, 2018	MN012999	C	15469	-0,028	79,766	-0,251	20,234	new annotation
MIQGO	Papilionoidea	Nymphalidae	Satyrinae	*Melanitis leda* (Linnaeus, 1758)	JF905446	C	15122	-0,037	79,804	-0,240	20,196	
MIQGO	Papilionoidea	Nymphalidae	Satyrinae	*Melanitis phedima* (Cramer, 1780)	KF590538	C	15142	-0,037	79,937	-0,242	20,063	
MIQGO	Papilionoidea	Nymphalidae	Satyrinae	*Minois dryas* Scopoli, 1763	MK521433	C	15195	-0,028	80,191	-0,222	19,809	
MIQGO	Papilionoidea	Nymphalidae	Satyrinae	*Mycalesis intermedia* Moore, 1892	MN610565	C	15386	-0,029	80,859	-0,235	19,141	
MIQGO	Papilionoidea	Nymphalidae	Satyrinae	*Mycalesis mineus* (Linnaeus, 1758)	KM244676	C	15267	-0,030	80,795	-0,231	19,205	
MIQGO	Papilionoidea	Nymphalidae	Satyrinae	*Neope pulaha* (Moore, [1858])	KF590543	C	15209	-0,039	79,151	-0,236	20,718	
MIQGO	Papilionoidea	Nymphalidae	Satyrinae	*Ninguta schrenckii* (Ménétriés, 1859)	KF881052	C	15261	-0,053	80,159	-0,226	19,822	
MIQGO	Papilionoidea	Nymphalidae	Satyrinae	*Oeneis urda* (Eversmann, 1847)	MN917147	C	15248	-0,022	79,958	-0,214	20,042	
MIQGO	Papilionoidea	Nymphalidae	Satyrinae	*Pararge aegeria* (Linnaeus, 1758)	KJ547676	C	15240	-0,034	80,459	-0,212	19,541	
MIQGO	Papilionoidea	Nymphalidae	Satyrinae	*Pararge aegeria* Linnaeus, 1758	LR990920	C	15237	-0,034	80,495	-0,213	19,505	new annotation
MIQGO	Papilionoidea	Nymphalidae	Satyrinae	*Ypthima akragas* Fruhstorfer, 1911	KF590553	C	15227	-0,054	81,861	-0,194	18,113	
MIQGO	Papilionoidea	Nymphalidae	Satyrinae	*Ypthima baldus* (Fabricius, 1775)	MN708051	C	15304	-0,045	80,809	-0,217	19,185	
MIQGO	Papilionoidea	Nymphalidae	Satyrinae	*Ypthima motschulskyi* (Bremer & Grey, 1852)	MN242788	C	15232	-0,051	81,847	-0,207	18,153	
MIQGO	Papilionoidea	Papilionidae	Baroniinae	*Baronia brevicornis* Bryk, 1913	LT999970	P	14918	0,003	80,111	-0,181	18,789	
MIQGO	Papilionoidea	Papilionidae	Papilioninae	*Atrophaneura alcinous* (Klug, 1836)	KJ540880	C	15266	-0,048	81,082	-0,217	18,918	
MIQGO	Papilionoidea	Papilionidae	Papilioninae	*Atrophaneura alcinous* (Klug, 1836)	LT999969	C	15779	-0,056	80,164	-0,196	19,836	
MIQGO	Papilionoidea	Papilionidae	Papilioninae	*Atrophaneura dixoni* (Grose-Smith, 1900)	LT999977	C	15238	-0,056	80,175	-0,215	19,707	
MIQGO	Papilionoidea	Papilionidae	Papilioninae	*Atrophaneura hedistus* (Matsumura, 1910)	MN012972	C	15135	-0,058	80,866	-0,210	19,134	new annotation
MIQGO	Papilionoidea	Papilionidae	Papilioninae	*Battus polydamas* (Linnaeus, 1758)	LS974637	C	15462	-0,013	79,091	-0,235	20,903	
4QGO	Papilionoidea	Papilionidae	Papilioninae	*Bhutanitis thaidina* Blanchard, 1871	MN012973	C	16044	-0,020	81,339	-0,236	18,661	new annotation
MIQGO	Papilionoidea	Papilionidae	Papilioninae	*Euryades corethrus* (Boisduval, 1836)	LT999972	C	15133	-0,052	81,174	-0,204	18,826	
MIQGO	Papilionoidea	Papilionidae	Papilioninae	*Eurytides dolicaon* (cramer, 1776)	LR031513	C	15354	0,007	79,836	-0,195	20,040	
MIQGO	Papilionoidea	Papilionidae	Papilioninae	*Graphium androcles* (Boisduval, 1836)	LT999976	C	15104	-0,033	80,204	-0,204	18,922	
iGO	Papilionoidea	Papilionidae	Papilioninae	*Graphium antiphates* (Cramer, 1775)	MN013005	P	14642	-0,019	80,208	-0,225	19,792	new annotation
MIQGO	Papilionoidea	Papilionidae	Papilioninae	*Graphium chironides* (Honrath, 1884)	KP159289	C	15235	-0,014	80,400	-0,216	19,600	
MIQGO	Papilionoidea	Papilionidae	Papilioninae	*Graphium confucius* Hu, Duan & Cotton, 2018	MN013012	C	15242	0,008	79,156	-0,226	20,844	new annotation
MIQGO	Papilionoidea	Papilionidae	Papilioninae	*Graphium doson* (Felder & Felder, 1864)	MK144328	C	15285	-0,016	80,877	-0,202	19,123	
MIQGO	Papilionoidea	Papilionidae	Papilioninae	*Graphium eurous asakurae* (Matsumura, 1908)	MW549198	C	15228	-0,010	80,391	-0,200	19,609	

GO	SUPERFAMILY	FAMILY	SUBFAMILY	SPECIES	GenBank	C/P	Size	AT-skew	AT%	GC-skew	GC%	NOTE
MIQGO	Papilionoidea	Papilionidae	Papilioninae	*Graphium eurypylus* (Linnaeus, 1758)	MN012987	C	15193	-0,015	80,833	-0,203	19,167	new annotation
MIQGO	Papilionoidea	Papilionidae	Papilioninae	*Graphium leechi* (Rothschild, 1895)	KX011066	C	15836	-0,012	81,031	-0,212	18,969	
MIQGO	Papilionoidea	Papilionidae	Papilioninae	*Graphium mullah chungianus* (Murayama, 1961)	MW549197	C	15240	-0,009	80,965	-0,201	19,035	
MIQGO	Papilionoidea	Papilionidae	Papilioninae	*Graphium nomius* (Esper, 1793)	MN013014	C	15113	-0,037	80,097	-0,207	19,903	new annotation
MIQGO	Papilionoidea	Papilionidae	Papilioninae	*Graphium parus* (de Nicéville, 1886)	MT198821	C	15231	0,003	79,929	-0,218	20,071	
MIQGO	Papilionoidea	Papilionidae	Papilioninae	*Graphium sarpedon* (Linnaeus, 1758)	MN012989	C	15306	-0,005	80,655	-0,219	19,228	new annotation
MIQGO	Papilionoidea	Papilionidae	Papilioninae	*Graphium timur* (Ney, 1911)	KJ472924	C	15226	-0,008	80,363	-0,197	19,637	
MIQGO	Papilionoidea	Papilionidae	Papilioninae	*Iphiclides podalirius* (Linnaeus, 1758)	MK507891	P	14914	0,013	80,636	-0,184	18,338	
MIQGO	Papilionoidea	Papilionidae	Papilioninae	*Lamproptera curius* (Fabricius, 1787)	KJ141168	C	15277	-0,008	80,474	-0,193	19,526	
MIQGO	Papilionoidea	Papilionidae	Papilioninae	*Lamproptera meges* (Zinken-Sommer, 1831)	LT999978	C	15113	0,006	80,811	-0,195	19,189	
MIQGO	Papilionoidea	Papilionidae	Papilioninae	*Losaria neptunus* (Guérin-Méneville, 1840)	LT999979	P	14901	-0,041	80,632	-0,186	19,368	
MIQGO	Papilionoidea	Papilionidae	Papilioninae	*Meandrusa payeni* (Boisduval, 1836)	MN013000	C	15249	0,004	80,156	-0,237	19,844	new annotation
MIQGO	Papilionoidea	Papilionidae	Papilioninae	*Meandrusa sciron* (Leech, 1890)	LS975123	P	14875	-0,006	79,408	-0,219	20,578	
MIQGO	Papilionoidea	Papilionidae	Papilioninae	*Mimoides lysithous* (Hübner, [1821])	LT999982	C	15038	-0,008	80,842	-0,203	19,158	
MIQGO	Papilionoidea	Papilionidae	Papilioninae	*Ornithoptera priamus* (Linnaeus, 1758)	LT999981	P	14804	-0,040	79,202	-0,254	20,798	
MIQGO	Papilionoidea	Papilionidae	Papilioninae	*Ornithoptera richmondia* (Gray, [1853])	LT999980	P	14763	-0,042	79,293	-0,246	20,687	
MIQGO	Papilionoidea	Papilionidae	Papilioninae	*Pachliopta aristolochiae* (Fabricius, 1775)	KU950357	C	15232	-0,037	81,624	-0,199	18,376	
MIQGO	Papilionoidea	Papilionidae	Papilioninae	*Papilio bianor* Cramer, 1777	MN013007	C	15339	-0,013	80,631	-0,213	19,369	new annotation
MIQGO	Papilionoidea	Papilionidae	Papilioninae	*Papilio bianor* Cramer, 1777	JN019809	C	15340	-0,015	80,554	-0,212	19,446	
MIQGO	Papilionoidea	Papilionidae	Papilioninae	*Papilio bianor* Cramer, 1777	KC433409	C	15332	-0,014	80,648	-0,213	19,352	
MIQGO	Papilionoidea	Papilionidae	Papilioninae	*Papilio dardanus* Brown, 1776	KX033351	C	15349	-0,014	79,393	-0,249	20,451	
MIQGO	Papilionoidea	Papilionidae	Papilioninae	*Papilio dardanus* Brown, 1776	KX033352	C	15311	-0,013	80,511	-0,226	19,333	
MIQGO	Papilionoidea	Papilionidae	Papilioninae	*Papilio dardanus* Brown, 1776	KX033358	C	15346	-0,007	79,063	-0,265	20,937	
MIQGO	Papilionoidea	Papilionidae	Papilioninae	*Papilio dardanus polytrophus* Rothschild & Jordan, 1903	KX033353	P	15084	-0,005	78,275	-0,272	21,062	
MIQGO	Papilionoidea	Papilionidae	Papilioninae	*Papilio demoleus* Linnaeus, 1758	MN013008	C	15216	-0,031	80,935	-0,189	19,065	new annotation
MIQGO	Papilionoidea	Papilionidae	Papilioninae	*Papilio demoleus* Linnaeus, 1758	KR024009	C	15249	-0,028	80,884	-0,186	19,116	
MIQGO	Papilionoidea	Papilionidae	Papilioninae	*Papilio glaucus* Linnaeus, 1758	KR822739	C	15306	-0,006	80,426	-0,221	19,574	
MIQGO	Papilionoidea	Papilionidae	Papilioninae	*Papilio helenus* Linnaeus, 1758	MN013010	C	15352	-0,004	80,113	-0,249	19,887	new annotation
MIQGO	Papilionoidea	Papilionidae	Papilioninae	*Papilio helenus* Linnaeus, 1758	KM244656	C	15349	-0,005	80,096	-0,250	19,904	
MIQGO	Papilionoidea	Papilionidae	Papilioninae	*Papilio maackii* Ménétries, 1859	KC433408	C	15357	-0,014	80,693	-0,214	19,307	
MIQGO	Papilionoidea	Papilionidae	Papilioninae	*Papilio machaon* Linnaeus, 1758	LS975121	C	15179	-0,026	80,302	-0,205	19,685	
MIQGO	Papilionoidea	Papilionidae	Papilioninae	*Papilio maraho* (Shiraki & Sonan, 1934)	FJ810212	C	16094	0,006	80,496	-0,262	19,430	
MIQGO	Papilionoidea	Papilionidae	Papilioninae	*Papilio memnon* Linnaeus, 1758	LD700014	C	15296	0,007	79,812	-0,246	20,188	
MIQGO	Papilionoidea	Papilionidae	Papilioninae	*Papilio memnon* Linnaeus, 1758	MH981597	C	15262	-0,008	79,741	-0,246	20,259	
MIQGO	Papilionoidea	Papilionidae	Papilioninae	*Papilio paris* Linnaeus, 1758	MN629008	C	15363	-0,016	80,525	-0,228	19,417	
MIQGO	Papilionoidea	Papilionidae	Papilioninae	*Papilio polytes* Linnaeus, 1758	KM014701	C	15256	-0,023	81,142	-0,208	18,858	
MIQGO	Papilionoidea	Papilionidae	Papilioninae	*Papilio protenor* Cramer, 1775	MN013015	C	15257	-0,016	80,514	-0,233	19,486	new annotation
MIQGO	Papilionoidea	Papilionidae	Papilioninae	*Papilio protenor* Cramer, 1775	KY272622	C	15268	-0,018	80,495	-0,228	19,505	
MIQGO	Papilionoidea	Papilionidae	Papilioninae	*Papilio rex* Oberthür, 1886	KX033354	C	15239	-0,021	79,526	-0,236	20,474	
MIQGO	Papilionoidea	Papilionidae	Papilioninae	*Papilio slateri* Hewitson, 1857	LT999985	P	14821	-0,010	80,595	-0,208	19,398	
MIQGO	Papilionoidea	Papilionidae	Papilioninae	*Papilio syfanius* Oberthür, 1886	KJ396621	C	15359	-0,020	80,604	-0,205	19,396	
MIQGO	Papilionoidea	Papilionidae	Papilioninae	*Papilio thoas* Linnaeus, 1771	MW548255	C	15258	-0,009	80,646	-0,225	19,347	
MIQGO	Papilionoidea	Papilionidae	Papilioninae	*Papilio xuthus* Linnaeus, 1767	KT922004	C	15359	-0,013	80,526	-0,228	19,474	
MIQGO	Papilionoidea	Papilionidae	Papilioninae	*Papilio xuthus* Linnaeus, 1767	KU356933	C	15359	-0,013	80,467	-0,226	19,533	
MIQGO	Papilionoidea	Papilionidae	Papilioninae	*Papilio xuthus* Linnaeus, 1767	MW548256	C	15350	-0,013	80,469	-0,229	19,466	
MIQGO	Papilionoidea	Papilionidae	Papilioninae	*Parides ascanius* (Cramer, 1775)	LS974638	C	15212	-0,054	79,306	-0,213	19,005	
MIQGO	Papilionoidea	Papilionidae	Papilioninae	*Pharmacophagus antenor* (Drury, 1775)	LS975119	C	15096	-0,048	80,975	-0,212	18,866	
MIQGO	Papilionoidea	Papilionidae	Papilioninae	*Protesilaus protesilaus* (Linnaeus, 1758)	LT999984	C	14964	-0,004	81,917	-0,173	18,083	
MIQGO	Papilionoidea	Papilionidae	Papilioninae	*Protographium marcellus* (Cramer, 1777)	MK507890	P	14784	-0,007	78,355	-0,179	18,676	
MIQGO	Papilionoidea	Papilionidae	Papilioninae	*Teinopalpus aureus* Mell, 1923	HM563681	C	15242	-0,005	79,806	-0,238	20,194	
MIQGO	Papilionoidea	Papilionidae	Papilioninae	*Teinopalpus aureus* Mell, 1923	KP941013	C	15234	-0,003	79,828	-0,241	20,172	
MIQGO	Papilionoidea	Papilionidae	Papilioninae	*Teinopalpus aureus* Mell, 1923	KP941014	C	15234	-0,003	79,848	-0,241	20,146	
MIQGO	Papilionoidea	Papilionidae	Papilioninae	*Teinopalpus aureus* Mell, 1923	KP941015	C	15234	-0,004	79,795	-0,240	20,205	
MIQGO	Papilionoidea	Papilionidae	Papilioninae	*Teinopalpus aureus* Mell, 1923	KP941016	C	15234	-0,003	79,808	-0,240	20,192	
MIQGO	Papilionoidea	Papilionidae	Papilioninae	*Teinopalpus aureus* Mell, 1923	KP941017	C	15234	-0,004	79,828	-0,240	20,172	
MIQGO	Papilionoidea	Papilionidae	Papilioninae	*Teinopalpus aureus* Mell, 1923	KP941018	C	15235	-0,004	79,810	-0,241	20,190	
MIQGO	Papilionoidea	Papilionidae	Papilioninae	*Teinopalpus aureus* Mell, 1923	KP941019	C	15234	-0,004	79,815	-0,240	20,185	
MIQGO	Papilionoidea	Papilionidae	Papilioninae	*Teinopalpus imperialis* Hope, 1843	KR018842	C	15229	-0,005	79,592	-0,232	20,408	
MIQGO	Papilionoidea	Papilionidae	Papilioninae	*Teinopalpus imperialis* Hope, 1843	LS975139	C	15229	-0,004	79,565	-0,236	20,356	
MIQGO	Papilionoidea	Papilionidae	Parnassiinae	*Allancastria cerisyi* Godart, 1824	LS974636	P	15279	-0,021	80,575	-0,205	18,738	
MIQGO	Papilionoidea	Papilionidae	Parnassiinae	*Archon apollinus* (Herbst, 1789)	LT999971	P	14971	-0,020	81,077	-0,175	18,229	
MIQGO	Papilionoidea	Papilionidae	Parnassiinae	*Bhutanitis mansfieldi* (Riley 1939)	LT999973	P	14994	-0,017	81,539	-0,213	18,447	
MIQGO	Papilionoidea	Papilionidae	Parnassiinae	*Luehdorfia chinensis* Leech, 1893	KM453725	C	16028	-0,008	81,963	-0,211	18,037	
MIQGO	Papilionoidea	Papilionidae	Parnassiinae	*Luehdorfia chinensis* Leech, 1893	KM453726	C	15580	-0,007	81,624	-0,208	18,376	
MIQGO	Papilionoidea	Papilionidae	Parnassiinae	*Luehdorfia chinensis* Leech, 1893	KM453727	C	15580	-0,007	81,605	-0,209	18,395	
MIQGO	Papilionoidea	Papilionidae	Parnassiinae	*Luehdorfia japonica* Leech, 1889	LT999996	P	15347	-0,011	81,449	-0,196	18,551	
MIQGO	Papilionoidea	Papilionidae	Parnassiinae	*Luehdorfia taibai* Chou, 1994	KC952673	C	15553	-0,009	81,463	-0,202	18,537	
MIQGO	Papilionoidea	Papilionidae	Parnassiinae	***Parnassius apollo* (Linnaeus, 1758)**	**ON087696**	C	15352	-0,012	81,364	-0,194	18,636	new mitogenome
MIQGO	Papilionoidea	Papilionidae	Parnassiinae	*Parnassius apollo* (Linnaeus, 1758)	KF746065	C	15404	-0,016	81,304	-0,190	18,696	
MIQGO	Papilionoidea	Papilionidae	Parnassiinae	*Parnassius apollonius* (Eversmann, 1847)	MG252290	C	15381	-0,014	81,737	-0,190	18,263	
MIQGO	Papilionoidea	Papilionidae	Parnassiinae	*Parnassius bremeri* Bremer, 1864	FJ871125	C	15389	-0,011	81,266	-0,194	18,734	
MIQGO	Papilionoidea	Papilionidae	Parnassiinae	*Parnassius bremeri* Bremer, 1864	HM243588	C	15390	-0,019	81,046	-0,194	18,954	
MIQGO	Papilionoidea	Papilionidae	Parnassiinae	*Parnassius cephalus* Grumm-Grshimailo, 1891	KP100655	C	15343	-0,012	81,399	-0,193	18,601	
MIQGO	Papilionoidea	Papilionidae	Parnassiinae	*Parnassius choui* Huang & Shi, 1994	KY072797	C	15367	-0,011	81,460	-0,198	18,540	
MIQGO	Papilionoidea	Papilionidae	Parnassiinae	*Parnassius epaphus* Oberthür, 1879	KM373898	C	15458	-0,018	81,395	-0,195	18,605	
MIQGO	Papilionoidea	Papilionidae	Parnassiinae	*Parnassius imperator* Oberthür, 1883	KM507326	C	15424	-0,013	81,088	-0,207	18,912	
MIQGO	Papilionoidea	Papilionidae	Parnassiinae	*Parnassius mercurius* Grum-Grshimailo, 1891	MN728989	C	15372	-0,014	81,447	-0,194	18,553	
MIQGO	Papilionoidea	Papilionidae	Parnassiinae	*Parnassius nomion* von Waldheim, 1823	MF496134	C	15362	-0,016	81,324	-0,190	18,676	
MIQGO	Papilionoidea	Papilionidae	Parnassiinae	*Parnassius smintheus* Doubleday, [1847]	LT999983	C	15411	-0,012	80,618	-0,195	18,889	
MIQGO	Papilionoidea	Papilionidae	Parnassiinae	*Sericinus montela* Gray, 1852	HQ259122	C	15243	-0,008	80,955	-0,223	19,045	
MIQGO	Papilionoidea	Papilionidae	Parnassiinae	*Zerynthia polyxena* (Denis & Schiffermüller, 1775)	MK507888	C	15092	-0,024	80,625	-0,214	19,368	
MIQGO	Papilionoidea	Pieridae	Coliadinae	*Catopsilia pomona* (Fabricius, 1775)	MN012978	C	15145	-0,031	81,367	-0,185	18,633	new annotation
MIQGO	Papilionoidea	Pieridae	Coliadinae	*Catopsilia pomona* Fabricius, 1775	JX274649	C	15142	-0,029	81,290	-0,183	18,710	
MIQGO	Papilionoidea	Pieridae	Coliadinae	*Colias croceus* (Geoffroy in Fourcroy, 1785)	HG991990	C	15183	-0,017	81,328	-0,199	18,672	new annotation
MIQGO	Papilionoidea	Pieridae	Coliadinae	*Colias croceus* (Geoffroy in Fourcroy, 1785)	KM592967	C	15254	-0,017	80,766	-0,200	18,579	
MIQGO	Papilionoidea	Pieridae	Coliadinae	*Colias erate* (Esper, 1805)	KP715146	C	15184	-0,019	81,342	-0,199	18,658	
MIQGO	Papilionoidea	Pieridae	Coliadinae	*Colias fieldii* Ménétriés, 1855	MT371042	C	15150	-0,017	80,964	-0,196	19,036	
MIQGO	Papilionoidea	Pieridae	Coliadinae	*Eurema blanda* Boisduval, 1836	KU160635	C	15123	-0,020	80,566	-0,242	19,434	
MIQGO	Papilionoidea	Pieridae	Coliadinae	*Eurema hecabe* (Linnaeus, 1758)	KF881047	C	15112	-0,027	80,545	-0,224	19,455	
MIQGO	Papilionoidea	Pieridae	Coliadinae	*Eurema hecabe* (Linnaeus, 1758)	KX681479	C	15124	-0,028	80,508	-0,222	19,492	
MIQGO	Papilionoidea	Pieridae	Coliadinae	*Gandaca harina* (Horsfield, 1829)	MN012988	C	15219	-0,040	81,175	-0,195	18,825	new annotation
MIQGO	Papilionoidea	Pieridae	Coliadinae	*Gonepteryx mahaguru* Gistel, 1857	KF881048	C	15221	-0,034	80,875	-0,198	19,125	
MIQGO	Papilionoidea	Pieridae	Coliadinae	*Gonepteryx rhamni* (Linnaeus, 1758)	KC461234	C	15203	-0,027	80,293	-0,206	19,707	
MIQGO	Papilionoidea	Pieridae	Coliadinae	*Gonepteryx rhamni* (Linnaeus, 1758)	MF536894	C	15223	-0,034	80,845	-0,197	19,155	
MIQGO	Papilionoidea	Pieridae	Coliadinae	*Zerene cesonia* (Stoll, 1790)	CM022631	C	15138	-0,018	80,790	-0,204	19,210	new annotation
MIQGO	Papilionoidea	Pieridae	Dismorphiinae	*Leptidea morsei* (Fenton, 1881)	MN012998	C	15688	-0,038	80,928	-0,205	19,072	new annotation
MIQGO	Papilionoidea	Pieridae	Dismorphiinae	*Leptidea morsei* (Fenton, 1881)	JX274648	C	15122	-0,040	80,241	-0,197	19,759	
MIQGO	Papilionoidea	Pieridae	Dismorphiinae	*Leptidea sinapis* (Linnaeus, 1758)	FR990201	C	15959	-0,038	81,120	-0,205	18,880	new annotation
MIQGO	Papilionoidea	Pieridae	Pierinae	*Anthocharis bambusarum* (Oberthür, 1876)	KC465748	C	15180	-0,025	80,211	-0,218	19,789	
MIQGO	Papilionoidea	Pieridae	Pierinae	*Anthocharis cardamines* (Linnaeus, 1758)	FR989981	C	15204	-0,029	80,538	-0,217	19,462	new annotation
MIQGO	Papilionoidea	Pieridae	Pierinae	*Anthocharis scolymus* (Butler, 1866)	MT499329	C	15230	-0,032	80,499	-0,220	19,501	
MIQGO	Papilionoidea	Pieridae	Pierinae	*Aporia bieti* (Oberthür, 1884)	MN012970	C	15139	-0,015	80,659	-0,236	19,341	new annotation
MIQGO	Papilionoidea	Pieridae	Pierinae	*Aporia bieti* (Oberthür, 1884)	KX495165	C	15147	-0,010	80,049	-0,236	19,951	
MIQGO	Papilionoidea	Pieridae	Pierinae	*Aporia crataegi* (Linnaeus, 1758)	JN796473	C	15140	-0,024	81,255	-0,219	18,745	
MIQGO	Papilionoidea	Pieridae	Pierinae	*Aporia crataegi* (Linnaeus, 1758)	MF576059	C	15148	-0,024	81,238	-0,220	18,762	
MIQGO	Papilionoidea	Pieridae	Pierinae	*Aporia hippia* (Bremer, 1861)	KX495166	C	15154	0,001	79,557	-0,263	20,443	
MIQGO	Papilionoidea	Pieridae	Pierinae	*Aporia intercostata* O. Bang-Haas, 1927	KC461928	C	15144	-0,004	80,388	-0,246	19,612	
MIQGO	Papilionoidea	Pieridae	Pierinae	*Aporia largeteaui* (Oberthür, 1881)	MW284889	C	15143	0,001	79,872	-0,260	20,128	
MIQGO	Papilionoidea	Pieridae	Pierinae	*Aporia martineti* (Oberthür, 1884)	KX495167	C	15192	-0,015	80,602	-0,237	19,398	
MIQGO	Papilionoidea	Pieridae	Pierinae	*Appias albina* (Boisduval, 1836)	MN643595	C	15193	-0,029	79,570	-0,216	20,430	
MIQGO	Papilionoidea	Pieridae	Pierinae	*Appias lyncida* Cramer, 1777	MK263224	C	15082	-0,037	80,188	-0,220	19,812	
MIQGO	Papilionoidea	Pieridae	Pierinae	*Appias nero* (Wallace, 1867)	MK263223	C	15099	-0,026	78,840	-0,236	21,160	
MIQGO	Papilionoidea	Pieridae	Pierinae	*Appias remedios* Schröder, H & Treadaway, 1980	MF576060	C	15118	-0,017	80,196	-0,214	19,804	

GO	SUPERFAMILY	FAMILY	SUBFAMILY	SPECIES	GenBank	C/P	Size	AT-skew	AT%	GC-skew	GC%	NOTE
MIQGO	Papilionoidea	Pieridae	Pierinae	*Baltia butleri* Alphéraky, 1889	MH380204	C	15124	0,013	79,708	-0,251	20,292	
MIQGO	Papilionoidea	Pieridae	Pierinae	*Cepora nadina* (H. Lucas, 1852)	MN012977	C	15229	-0,014	79,526	-0,236	20,474	new annotation
MIQGO	Papilionoidea	Pieridae	Pierinae	*Delias hyparete* (Linnaeus, 1758)	JX094279	C	15186	-0,017	79,843	-0,229	20,157	
MIQGO	Papilionoidea	Pieridae	Pierinae	*Delias pasithoe* (Linnaeus, 1767)	MN012983	C	15204	0,000	79,203	-0,264	20,797	new annotation
MIQGO	Papilionoidea	Pieridae	Pierinae	*Delias pasithoe* (Linnaeus, 1767)	MK252291	C	15203	-0,001	79,195	-0,263	20,805	
MIQGO	Papilionoidea	Pieridae	Pierinae	*Hebomoia glaucippe* (Linnaeus, 1758)	MN012992	C	15138	-0,034	79,647	-0,235	20,353	new annotation
MIQGO	Papilionoidea	Pieridae	Pierinae	*Mesapia peloria* (Hewitson, 1853)	KX495168	P	15159	-0,018	81,107	-0,229	18,893	
MIQGO	Papilionoidea	Pieridae	Pierinae	*Pieris brassicae* (Linnaeus, 1758)	LR989948	C	15324	0,016	79,340	-0,226	20,660	new annotation
MIQGO	Papilionoidea	Pieridae	Pierinae	*Pieris canidia* (Sparrman, 1768)	KP162184	C	15153	0,013	79,661	-0,226	20,339	
MIQGO	Papilionoidea	Pieridae	Pierinae	*Pieris melete* Menetries, 1857	EU597124	C	15140	0,012	79,782	-0,222	20,218	
MIQGO	Papilionoidea	Pieridae	Pierinae	*Pieris napi* (Linnaeus, 1758)	HG993187	C	15095	0,011	80,139	-0,227	19,861	new annotation
MIQGO	Papilionoidea	Pieridae	Pierinae	*Pieris napi* (Linnaeus, 1758)	MT576638	C	15178	0,011	80,076	-0,224	19,924	
MIQGO	Papilionoidea	Pieridae	Pierinae	*Pieris rapae* (Linnaeus, 1758)	LR990608	C	15161	0,014	79,665	-0,233	20,335	new annotation
MIQGO	Papilionoidea	Pieridae	Pierinae	*Pieris rapae* Linnaeus, 1758	GQ398376	C	15164	0,014	79,662	-0,232	20,338	
MIQGO	Papilionoidea	Pieridae	Pierinae	*Pieris rapae* Linnaeus, 1758	HM156697	C	15157	0,014	79,739	-0,232	20,261	
MIQGO	Papilionoidea	Pieridae	Pierinae	*Pontia callidice* Hübner, 1799-1800	MH380206	C	15109	-0,003	79,866	-0,229	20,134	
MIQGO	Papilionoidea	Pieridae	Pierinae	*Pontia daplidice* (Linnaeus, 1758)	MH380207	C	15124	-0,001	79,926	-0,218	20,074	
MIQGO	Papilionoidea	Pieridae	Pierinae	*Pontia edusa* Fabricius, 1777	MN013009	C	15127	-0,001	79,903	-0,219	20,097	new annotation
MIQGO	Papilionoidea	Pieridae	Pierinae	*Pontia edusa* Fabricius, 1777	MK252290	C	15125	-0,001	79,914	-0,218	20,086	
MIQGO	Papilionoidea	Pieridae	Pierinae	*Prioneris clemanthe* Doubleday, 1846	MK263225	C	15131	-0,022	80,761	-0,233	19,239	
MIQGO	Papilionoidea	Pieridae	Pierinae	*Talbotia nagana* (Moore 1884)	MH380205	C	15155	0,002	79,815	-0,221	20,185	
MIQGO	Papilionoidea	Pieridae	Pierinae	*Talbotia nagana* (Moore 1884)	MW013541	C	15155	0,002	79,729	-0,220	20,271	
MIQGO	Papilionoidea	Riodinidae	Nemeobiinae	*Abisara fylloides* Moore, 1902	HQ259069	C	15301	-0,028	81,171	-0,203	18,829	
MIQGO	Papilionoidea	Riodinidae	Nemeobiinae	*Dodona durga* (Kollar, 1844)	MN012981	C	15349	-0,025	82,018	-0,149	17,982	new annotation
MIQGO	Papilionoidea	Riodinidae	Nemeobiinae	*Dodona eugenes* Bates, 1868	MT890732	C	15680	-0,019	81,014	-0,167	18,986	
MIQGO	Papilionoidea	Riodinidae	Nemeobiinae	*Zemeros flegyas* (Cramer, 1780)	MK521434	C	15219	-0,035	81,792	-0,160	18,208	
MIQGO	Papilionoidea	Riodinidae	Riodininae	*Apodemia mormo* (Felder & Felder, 1859)	KJ647171	C	15262	-0,047	81,523	-0,192	18,477	

**C/P**	Complete/Partial genome
**Size**	Genome Size
**AT-skew**	
**AT%**	percentage of A+T
**GC-skew**	
**GC%**	percentage of G+C

Table S2. Mitogenomes of Papilionoidea excluded from the final data set.

GO	SUPERFAMILY	FAMILY	SUBFAMILY	SPECIES	GenBank	C/P	Size	NOTE
MIQGO	Papilionoidea	Hesperiidae	Hesperiinae	*Ampittia dioscorides* (Fabricius, 1793)	KM102732	**C**	15313	bad *atp6* and *cox3*; **excluded form final data set**
2S1GO	Papilionoidea	Hesperiidae	Pyrginae	*Ctenoptilum vasava* (Moore, [1866])	JF713818	**C**	15468	bad *cox1* and *trnK*; **excluded form final data set**
iGO	Papilionoidea	Hesperiidae	Pyrginae	*Pyrgus armoricanus* (Oberthür, 1910)	MT628570	P	9120	Incomplete gene order iGO; **excluded form final data set**
MIQGO	Papilionoidea	Nymphalidae	Apaturinae	*Apatura ilia* (Denis & Schiffermüller, 1775)	JF437925	**C**	15245	bad *atp6*; **excluded form final data set**
iGO	Papilionoidea	Nymphalidae	Charaxinae	*Polyura schreiber* (Godart, [1824])	KT232257	**C**	15420	iGO no *trnF*; **excluded form final data set**
iGO	Papilionoidea	Nymphalidae	Cyrestinae	*Dichorragia nesimachus* (Doyere, 1840)	KF990126	**P**	14367	iGO , some genes lacking; **excluded form final data set**
MIQGO	Papilionoidea	Nymphalidae	Danainae	*Tirumala limniace* (Cramer, [1775])	KJ784473	**C**	15285	bad *trnH*; **excluded form final data set**
MIQGO	Papilionoidea	Nymphalidae	Danainae	*Tirumala limniace* (Cramer, [1775])	KM593191	**C**	15275	bad sequence, multiple framshifts; **excluded form final data set**
MIQGO	Papilionoidea	Nymphalidae	Danainae	*Tirumala limniace* (Cramer, [1775])	KX889069	**C**	15278	bad sequence, multiple framshifts; **excluded form final data set**
MIQGO	Papilionoidea	Nymphalidae	Heliconiinae	*Acraea* (*Pareba*) *issoria* (Hübner, 1819)	GQ376195	**C**	15245	bad *nad5*; **excluded form final data set**
MIQGO	Papilionoidea	Nymphalidae	Heliconiinae	*Heliconius clysonymus* Latreille, [1817]	KP784455	**C**	15302	bad *rrnS* and CoRe; **excluded form final data set**
iGO	Papilionoidea	Nymphalidae	Nymphalinae	*Junonia genoveva* (Cramer, [1780])	KX267576	**P**	15206	iGO; **excluded form final data set**
iGO	Papilionoidea	Nymphalidae	Nymphalinae	*Junonia genoveva genoveva* Cramer, [1780]	KX423729	**C**	15233	iGO; **excluded form final data set**
MIQGO	Papilionoidea	Nymphalidae	Nymphalinae	*Junonia genoveva infuscata* C. & R. Felder, [1867]	KX423730	**P**	15145	multiple unresolved bases; **excluded form final data set**
MIQGO	Papilionoidea	Nymphalidae	Nymphalinae	*Junonia litoralis* Brevignon, 2009	KX267568	**P**	15214	multiple unresolved bases; **excluded form final data set**
MIQGO	Papilionoidea	Nymphalidae	Nymphalinae	*Junonia litoralis* Brevignon, 2010	KX267574	**C**	15177	multiple unresolved bases and bad trnF; **excluded form final data set**
MIQGO	Papilionoidea	Nymphalidae	Nymphalinae	*Junonia vestina vestina* C. Felder & R. Felder, 1867	KX423728	**C**	15191	bad *nad3* and *trnE*; **excluded form final data set**
MIQGO	Papilionoidea	Nymphalidae	Nymphalinae	*Precis andremiaja* (Boisduval, 1833)	MH917706	**C**	15239	bad *trnL1* and *trnC*; **excluded form final data set**
MIQGO	Papilionoidea	Nymphalidae	Nymphalinae	*Salamis anteva* (Ward, 1870)	MH917707	**C**	15201	bad *trnA* and *trnC*; **excluded form final data set**
MIQGO	Papilionoidea	Nymphalidae	Satyrinae	*Stichophthalma louisa* Wood-Mason, 1877	KP247523	**C**	15721	bad *nad3* and *trnH*; **excluded form final data set**
MIQGO	Papilionoidea	Nymphalidae	Satyrinae	*Triphysa phryne* (Pallas, 1771)	KF906487	**C**	15143	bad *nad2*; **excluded form final data set**
MIQGO	Papilionoidea	Papilionidae	Papilioninae	*Graphium sarpedon* (Linnaeus, 1758)	LT999975	**P**	15098	bad sequence; **excluded form final data set**
iGO	Papilionoidea	Papilionidae	Papilioninae	*Pachliopta kotzebuea* (Eschscholtz, 1821)	LS975120	**C**	15045	iGO, *trnE* lacking; **excluded form final data set**
MIQGO	Papilionoidea	Papilionidae	Papilioninae	*Papilio bianor* Cramer, 1777	KF859738	**C**	15357	different than conspecific sequences; **excluded form final data set**
MIQGO	Papilionoidea	Papilionidae	Papilioninae	*Papilio dardanus* Brown, 1776	JX313686	**C**	15337	multiple unresolved bases; **excluded form final data set**
MIQGO	Papilionoidea	Papilionidae	Papilioninae	*Papilio dardanus* Brown, 1776	KX033355	**C**	15338	multiple unresolved bases; **excluded form final data set**
MIQGO	Papilionoidea	Papilionidae	Papilioninae	*Papilio helenus* Linnaeus, 1758	KP247522	**C**	15607	bad sequence; **excluded form final data set**
MIQGO	Papilionoidea	Papilionidae	Papilioninae	*Papilio machaon* Linnaeus, 1758	HM243594	**C**	15185	bad *na5*, *rrnS*, and CoRe; **excluded form final data set**
MIQGO	Papilionoidea	Papilionidae	Papilioninae	*Papilio polytes* Linnaeus, 1758	KM215138	**C**	15260	bad *nad1*, *trnV* and *nad2*; **excluded form final data set**
iGO	Papilionoidea	Papilionidae	Papilioninae	*Papilio xuthus* Linnaeus, 1767	EF621724	**P**	13964	iGO; **excluded form final data set**
iGO	Papilionoidea	Papilionidae	Papilioninae	*Trogonoptera brookiana* Wallace, 1855	LT999986	**C**	15005	iGO; **excluded form final data set**
MIQGO	Papilionoidea	Papilionidae	Parnassiinae	*Hypermnestra helios* (Nickerl, 1846)	LS975127	**P**	14919	bad sequence; **excluded form final data set**
MIQGO	Papilionoidea	Papilionidae	Parnassiinae	*Luehdorfia chinensis* Leech, 1893	KU360130	**C**	15550	bad sequence; **excluded form final data set**
MIQGO	Papilionoidea	Pieridae	Coliadinae	*Eurema hecabe* (Linnaeus, 1758)	KC257480	**C**	15160	nad2 very different than conspecific taxa; **excluded form final data set**
iGO	Papilionoidea	Pieridae	Pierinae	*Delias pasithoe parthenope* Wallace, 1867	KM244698	**P**	13155	iGO; **excluded form final data set**
MIQGO	Papilionoidea	Pieridae	Pierinae	*Hebomoia glaucippe* (Linnaeus, 1758)	KC489093	**C**	15701	bad *trnL1*; **excluded form final data set**
iGO	Papilionoidea	Pieridae	Pierinae	*Pieris brassicae* (Linnaeus, 1758)	KM592974	**C**	12887	iGO; **excluded form final data set**
iGO	Papilionoidea	Pieridae	Pierinae	*Pieris rapae* Linnaeus, 1758	KM592973	**P**	9751	iGO; **excluded form final data set**

**iGO**	incomplete gene order

Table S3. Mitogenomes of Outgroups included in the final data set.

GO	SUPERFAMILY	FAMILY	SUBFAMILY	SPECIES	GenBank	C/P	Size	AT-skew	AT%	GC-skew	GC%
MIQGO	Alucitoidea	Alucitidae		*Alucita montana* Barnes & Lindsey, 1921	KJ508059	P	15272	0,001	79,970	-0,198	19,880
MIQGO	Bombycoidea	Bombycidae	Bombycinae	*Bombyx mandarina* (Moore, 1872)	AB070263	C	15928	0,055	81,680	-0,213	18,320
MIQGO	Bombycoidea	Bombycidae	Bombycinae	*Ernolatia moorei* (Moore, 1879)	MF100139	C	15377	0,016	80,757	-0,227	19,243
MIQGO	Bombycoidea	Bombycidae	Bombycinae	*Ocinara albicollis* (Walker, 1862)	MF100144	C	15439	0,004	81,378	-0,200	18,622
MIQGO	Bombycoidea	Bombycidae	Bombycinae	*Rondotia menciana* Moore, 1885	KC881286	C	15301	0,050	78,864	-0,260	21,136
MIQGO	Bombycoidea	Bombycidae	Oberthuerinae	*Andraca olivacea* Matsumura, 1927	MF100135	C	15880	0,033	79,509	-0,258	20,491
MIQGO	Bombycoidea	Bombycidae	Oberthuerinae	*Mustilia undulosa* Yang & Mao, 1995	MF100142	C	15720	0,030	80,134	-0,242	19,866
MIQGO	Bombycoidea	Bombycidae	Oberthuerinae	*Mustilizans hepatica* (Moore, 1879)	MF100141	C	15745	0,017	80,210	-0,214	19,790
MIQGO	Bombycoidea	Bombycidae	Oberthuerinae	*Oberthueria jiatongae* Zolotuhin & Xing Wang, 2013	MF100143	C	15673	0,014	79,678	-0,224	20,322
MIQGO	Bombycoidea	Brahmaeidae		*Brahmaea hearseyi* (White, 1862)	KU884326	C	15442	-0,007	80,806	-0,221	19,194
MIQGO	Bombycoidea	Endromidae		*Prismosticta fenestrata* Butler, 1880	MF100145	C	15772	0,016	81,176	-0,189	18,824
MIQGO	Bombycoidea	Eupterotidae		*Ganisa cyanogrisea* Mell, 1929	MF100140	C	15250	-0,001	79,121	-0,246	20,380
MIQGO	Bombycoidea	Saturniidae	Saturniinae	*Actias artemis aliena* (Butler, 1879)	KF927042	C	15243	-0,013	78,620	-0,251	21,380
MIQGO	Bombycoidea	Saturniidae	Saturniinae	*Antheraea assamensis* Helfer, 1837	KU301792	C	15312	-0,018	80,179	-0,222	19,821
MIQGO	Bombycoidea	Saturniidae	Saturniinae	*Attacus atlas* (Linnaeus, 1758)	KF006326	C	15282	0,004	79,302	-0,241	20,698
MIQGO	Bombycoidea	Saturniidae	Saturniinae	*Cricula trifenestrata* Helfer, 1837	KY644697	C	15425	0,011	77,679	-0,277	22,314
MIQGO	Bombycoidea	Saturniidae	Saturniinae	*Eriogyna pyretorum* Westwood, 1847	FJ685653	C	15327	-0,031	80,818	-0,205	19,182
MIQGO	Bombycoidea	Saturniidae	Saturniinae	*Neoris haraldi* Schawerda, 1922	MF664471	C	15383	-0,011	79,510	-0,219	20,230
MIQGO	Bombycoidea	Saturniidae	Saturniinae	*Samia canningi* (Hutton, 1860)	KJ159909	C	15384	-0,007	79,875	-0,227	20,125
MIQGO	Bombycoidea	Saturniidae	Saturniinae	*Saturnia boisduvalii* Eversmann, 1847	EF622227	C	15360	-0,024	80,625	-0,217	19,375
MIQGO	Bombycoidea	Saturniidae	Saturniinae	*Saturnia jonasii* (Butler, 1877)	MF346379	C	15261	-0,018	79,484	-0,214	20,516
MIQGO	Bombycoidea	Sphingidae	Macroglossinae	*Ampelophaga rubiginosa* Bremer & Grey, 1853	KT153024	C	15282	0,017	81,534	-0,189	18,466
MIQGO	Bombycoidea	Sphingidae	Macroglossinae	*Macroglossum stellatarum* Linnaeus, 1758	MG747645	C	15290	0,002	81,164	-0,193	18,836
MIQGO	Bombycoidea	Sphingidae	Macroglossinae	*Theretra japonica* (Boisduval, 1869)	MG655620	C	15399	0,023	80,356	-0,245	19,644
MIQGO	Bombycoidea	Sphingidae	Smerinthinae	*Parum colligata* (Walker, 1856)	MG888667	C	15288	0,005	81,057	-0,192	18,943
MIQGO	Bombycoidea	Sphingidae	Sphinginae	*Manduca sexta* (Linnaeus, 1763)	EU286785	C	15516	-0,005	81,787	-0,181	18,213
MIQGO	Bombycoidea	Sphingidae	Sphinginae	*Notonagemia analis scribae* (Austaut, 1911)	KU934302	C	15303	0,025	80,246	-0,212	19,754
MIQGO	Bombycoidea	Sphingidae	Sphinginae	*Psilogramma increta* (Walker 1865)	MF974243	C	15252	0,025	80,330	-0,221	19,670
MIQGO	Bombycoidea	Sphingidae	Sphinginae	*Sphinx morio* (Rothschild & Jordan, 1903)	KC470083	C	15299	0,001	81,169	-0,195	18,831
MIQGO	Copromorphoidea	Carposinidae		*Carposina sasakii* Matsumura, 1900	HQ840719	C	15611	0,031	81,500	-0,162	18,500
MIQGO	Cossoidea	Cossidae	Cossinae	*Eogystia hippolophaecolus* (Hua, Chou, Fang & Chen, 1990)	KC831443	C	15431	0,030	78,433	-0,269	21,567
MIQGO	Drepanoidea	Drepanidae	Drepaninae	*Drepana arcuata* Walker, 1855	KJ508053	P	15302	0,005	81,127	-0,180	18,723
MIQGO	Gelechioidea	Autostichidae	Symmocinae	*Oegoconia novimundi* (Busck, 1915)	KJ508036	P	15408	-0,032	77,603	-0,329	22,209
MIQGO	Gelechioidea	Cosmopterigidae	Chrysopeleiinae	*Perimede* sp.	KJ508041	P	15131	-0,057	80,424	-0,229	19,397
MIQGO	Gelechioidea	Elachistidae	Ethmiinae	*Ethmia eupostica* Powell, 1985	KJ508047	P	15347	-0,014	79,553	-0,237	20,317
MIQGO	Gelechioidea	Elachistidae	Stenomatinae	*Promalactis suzukiella* (Matsumura, 1931)	KM875542	C	15507	-0,025	81,486	-0,183	18,514
MIQGO	Gelechioidea	Gelechiidae	Anacampsinae	*Mesophleps albilinella* (Park, 1990)	KU366707	C	15274	-0,061	80,536	-0,229	19,464
MIQGO	Gelechioidea	Gelechiidae	Dichomeridinae	*Dichomeris ustalella* (Fabricius, 1794)	KU366706	C	15410	-0,037	81,116	-0,179	18,884
MIQGO	Gelechioidea	Gelechiidae	Gelechiinae	*Tecia solanivora* (Povolný, 1973)	KT326187	C	15251	-0,013	78,264	-0,224	21,736
MIQGO	Gelechioidea	Gelechiidae	Pexicopiinae	*Pectinophora gossypiella* Saunders, 1843	KM225795	C	15202	-0,007	80,687	-0,204	19,313
MIQGO	Gelechioidea	Oecophoridae	Oecophorinae	*Endrosis sarcitrella* (Linnaeus, 1758)	KJ508037	P	15317	-0,010	79,611	-0,226	20,232
MIQGO	Gelechioidea	Oecophoridae	Oecophorinae	*Stathmopoda auriferella* (Walker, 1864)	KX138529	C	15456	-0,059	81,496	-0,197	18,504
MIQGO	Gelechioidea	Stathmopodidae		*Atrijuglans hetaohei* Yang, 1977	KT581634	C	15379	-0,034	81,319	-0,186	18,681
MIQGO	Gelechioidea	Stathmopodidae		*Hieromantis kurokoi* Yasuda, 1988	KU605775	C	15208	-0,045	80,260	-0,203	19,740
MIQGO	Geometroidea	Geometridae	Ennominae	*Abraxas suspecta* (Warren, 1894)	KY095828	C	15537	0,021	80,871	-0,191	19,129
MIQGO	Geometroidea	Geometridae	Ennominae	*Apocheima cinerarius* Erschoff, 1874	KR478686	C	15661	0,029	80,538	-0,192	19,462
MIQGO	Geometroidea	Geometridae	Ennominae	*Biston panterinaria* (Bremer & Grey, 1853)	JX406146	C	15517	0,064	79,551	-0,260	20,442
MIQGO	Geometroidea	Geometridae	Ennominae	*Celenna* sp.	KM244697	C	15403	0,037	80,751	-0,211	19,249
MIQGO	Geometroidea	Geometridae	Ennominae	*Ectropis obliqua* Prout, 1915	MF417804	C	15475	0,006	80,931	-0,178	19,069
MIQGO	Geometroidea	Geometridae	Ennominae	*Jankowskia athleta* Oberthür, 1884	KR822683	C	15534	0,042	79,529	-0,217	20,471
MIQGO	Geometroidea	Geometridae	Ennominae	*Phthonandria atrilineata* (Butler, 1881)	EU569764	C	15499	0,007	81,025	-0,192	18,975
MIQGO	Geometroidea	Geometridae	Larentiinae	*Dysstroma truncata* (Hufnagel, 1767)	KJ508061	P	15828	-0,008	79,953	-0,201	19,901
MIQGO	Geometroidea	Geometridae	Larentiinae	*Operophtera brumata* (Linnaeus, 1758)	KP027400	C	15748	0,005	79,966	-0,209	20,034
MIQGO	Gracillarioidea	Gracillariidae		*Cameraria ohridella* Deschka & Dimic, 1986	KJ508042	P	15513	0,019	81,113	-0,178	18,739
MIQGO	Gracillarioidea	Gracillariidae		*Phyllonorycter froelichiella* (Zeller, 1839)	KJ508048	P	15538	0,010	82,025	-0,185	17,827
MIQGO	Lasiocampoidea	Lasiocampidae		*Apatelopteryx phenax* De Lajonquière, 1968	KJ508055	P	15552	0,027	80,331	-0,207	19,515
MIQGO	Lasiocampoidea	Lasiocampidae		*Dendrolimus houi* De Lajonquière, 1979	KY000409	C	15381	0,030	79,930	-0,243	20,070
MIQGO	Lasiocampoidea	Lasiocampidae		*Euthrix laeta* (Walker, 1855)	KU870700	C	15368	0,019	80,193	-0,213	19,807
LacoGO	Mimallonoidea	Mimallonidae		*Lacosoma valva* Schaus, 1905	KJ508050	P	16108	0,022	80,873	-0,196	18,922
MIQGO	Noctuoidea	Doidae		*Doa* sp.	KJ508058	P	15228	0,005	80,437	-0,200	19,412
MIQGO	Noctuoidea	Erebidae	Aganainae	*Asota plana lacteata* Butler, 1881	KJ173908	C	15416	-0,002	80,345	-0,238	19,655
MIQGO	Noctuoidea	Erebidae	Arctiinae	*Aglaomorpha histrio* (Walker, 1855)	KY800518	C	15472	-0,003	79,899	-0,254	20,088
MIQGO	Noctuoidea	Erebidae	Arctiinae	*Amata formosae* Butler, 1876	KC513737	C	15463	-0,027	79,493	-0,266	20,507
MIQGO	Noctuoidea	Erebidae	Arctiinae	*Callimorpha dominula* (Linnaeus, 1758)	KP973953	C	15496	-0,011	81,021	-0,201	18,979
MIQGO	Noctuoidea	Erebidae	Arctiinae	*Cyana* sp.	KM244679	C	15494	-0,014	81,199	-0,223	18,801
MIQGO	Noctuoidea	Erebidae	Arctiinae	*Hyphantria cunea* (Drury, 1773)	GU592049	C	15481	0,010	80,389	-0,230	19,611
MIQGO	Noctuoidea	Erebidae	Arctiinae	*Lemyra melli* (Daniel, 1943)	KP307017	C	15418	0,001	78,668	-0,225	21,332
MIQGO	Noctuoidea	Erebidae	Arctiinae	*Paraona staudingeri* Alphéraky, 1897	KY827330	C	15427	0,009	80,191	-0,251	19,809
MIQGO	Noctuoidea	Erebidae	Arctiinae	*Vamuna virilis* (Rothschild, 1913)	KJ364659	C	15417	0,000	80,392	-0,229	19,608
MIQGO	Noctuoidea	Erebidae	Herminiinae	*Hydrillodes lentalis* Guenee, 1854	MH013484	C	15570	-0,020	81,079	-0,207	18,921
MIQGO	Noctuoidea	Erebidae	Hypeninae	*Paragabara curvicornuta* Kononenko & Matov, 2010	KT362742	C	15532	-0,008	80,440	-0,226	19,553
MIQGO	Noctuoidea	Lymantriidae		*Euproctis cryptosticta* Collenette, 1934	KY996558	C	15462	0,011	79,860	-0,245	20,140
MIQGO	Noctuoidea	Lymantriidae		*Gynaephora alpherakii* (Grum-Grzhimailo, 1891)	KJ957168	C	15755	0,004	81,441	-0,263	18,559
MIQGO	Noctuoidea	Lymantriidae		*Lymantria dispar* (Linnaeus, 1758)	FJ617240	C	15569	0,016	79,877	-0,247	20,123
MIQGO	Noctuoidea	Lymantriidae		*Somena scintillans* (Walker, 1856)	MH051839	C	15410	-0,009	80,818	-0,248	19,182
MIQGO	Noctuoidea	Noctuidae	Amphipyrinae	*Sesamia inferens* (Walker, 1856)	JN039362	C	15413	-0,001	80,237	-0,230	19,763
MIQGO	Noctuoidea	Noctuidae	Amphipyrinae	*Spodoptera exigua* (Hübner, 1808)	JX316220	C	15365	0,010	80,931	-0,195	19,069
MIQGO	Noctuoidea	Noctuidae	Catocalinae	*Eudocima phalonia* (Linnaeus, 1763)	KY196412	C	15575	-0,012	80,687	-0,219	19,313
MIQGO	Noctuoidea	Noctuidae	Hadeninae	*Mythimna separata* Walker, 1865	KF730242	C	15332	-0,011	80,987	-0,196	19,013
MIQGO	Noctuoidea	Noctuidae	Heliothinae	*Australothis rubrescens* Walker, 1858	KF977797	C	15382	0,000	81,355	-0,187	18,645
MIQGO	Noctuoidea	Noctuidae	Heliothinae	*Helicoverpa armigera* (Hübner, [1808])	GU188273	C	15347	0,001	80,973	-0,192	19,027
MIQGO	Noctuoidea	Noctuidae	Noctuinae	*Agrotis ipsilon* (Hufnagel, 1766)	KF163965	C	15377	-0,006	81,251	-0,177	18,749
MIQGO	Noctuoidea	Noctuidae	Noctuinae	*Striacosta albicosta* (Smith, 1888)	KM488268	C	15553	0,012	79,329	-0,238	20,671
MIQGO	Noctuoidea	Noctuidae	Plusiinae	*Ctenoplusia limbirena* (Guenée, 1852)	KM244665	C	15306	-0,036	80,994	-0,174	19,006
MIQGO	Noctuoidea	Nolidae	Chloephorinae	*Gabala argentata* Butler, 1878	KJ410747	C	15337	-0,029	81,691	-0,174	18,309
MIQGO	Noctuoidea	Nolidae	Risobinae	*Risoba prominens* Moore, 1881	KJ396197	C	15343	-0,007	81,066	-0,176	18,934
MIQGO	Noctuoidea	Notodontidae	Pygaerinae	*Clostera anachoreta* (Denis & Schiffermüller, 1775)	KX108766	C	15456	-0,019	80,719	-0,217	19,281
MIQGO	Noctuoidea	Notodontidae	Thaumetopoeinae	*Ochrogaster lunifer* Herrich-Schäffer, 1855	AM946601	C	15593	0,030	77,843	-0,318	22,157
MIQGO	Pterophoroidea	Pterophoridae	Pterophorinae	*Emmelina monodactyla* (Linnaeus, 1758)	KJ508063	P	15252	-0,018	80,488	-0,157	19,361
MIQGO	Pyraloidea	Crambidae	Crambinae	*Chilo suppressalis* (Walker, 1863)	HQ860290	C	15465	0,008	79,703	-0,243	20,297
MIQGO	Pyraloidea	Crambidae	Crambinae	*Diatraea saccharalis* (Fabricius, 1794)	FJ240227	C	15490	0,021	80,019	-0,258	19,981
MIQGO	Pyraloidea	Crambidae	Crambinae	*Pseudargyria interruptella* (Walker, 1866)	KP071469	C	15231	-0,011	79,397	-0,216	20,603
MIQGO	Pyraloidea	Crambidae	Evergestinae	*Evergestis junctalis* (Warren, 1892)	KP347976	C	15438	-0,015	81,079	-0,168	18,921
MIQGO	Pyraloidea	Crambidae	Nymphulinae	*Elophila interruptalis* (Pryer, 1877)	KC894961	C	15351	-0,011	80,321	-0,229	19,679
MIQGO	Pyraloidea	Crambidae	Nymphulinae	*Paracymoriza distinctalis* (Leech, 1889)	KF859965	C	15354	-0,002	82,265	-0,155	17,735
MIQGO	Pyraloidea	Crambidae	Nymphulinae	*Parapoynx crisonalis* (Walker, 1859)	KT443883	C	15374	0,017	82,028	-0,153	17,972
MIQGO	Pyraloidea	Crambidae	Pyraustinae	*Cnaphalocrocis medinalis* (Guenée, 1854)	JN246082	C	15388	-0,015	81,940	-0,175	18,060
MIQGO	Pyraloidea	Crambidae	Pyraustinae	*Loxostege sticticalis* (Linnaeus, 1761)	KR080490	C	15218	0,002	80,819	-0,191	19,181
MIQGO	Pyraloidea	Crambidae	Schoenobiinae	*Scirpophaga incertulas* (Walker, 1863)	KF751706	C	15220	0,029	77,168	-0,320	22,832
MIQGO	Pyraloidea	Crambidae	Scopariinae	*Eudonia angustea* (J. Curtis, 1827)	KJ508052	P	15386	-0,022	81,431	-0,190	18,419
MIQGO	Pyraloidea	Crambidae	Spilomelinae	*Conogethes punctiferalis* (Guenée, 1854)	JX448619	C	15355	-0,025	80,599	-0,207	19,401
MIQGO	Pyraloidea	Crambidae	Spilomelinae	*Glyphodes quadrimaculalis* Bremer & Grey, 1853	KF234079	C	15255	-0,007	80,800	-0,192	19,200
MIQGO	Pyraloidea	Crambidae	Spilomelinae	*Haritalodes derogata* (Fabricius, 1775)	KR233479	C	15253	0,002	80,699	-0,218	19,301
MIQGO	Pyraloidea	Crambidae	Spilomelinae	*Maruca testulalis* (Geyer, 1832)	KJ623250	C	15110	-0,005	80,814	-0,171	19,186
MIQGO	Pyraloidea	Crambidae	Spilomelinae	*Nomophila noctuella* (Denis & Schiffermüller, 1775)	KM244688	C	15309	0,002	81,410	-0,176	18,590
MIQGO	Pyraloidea	Crambidae	Spilomelinae	*Omiodes indicata* (Fabricius, 1775)	MG770232	C	15367	-0,012	81,577	-0,162	18,423
MIQGO	Pyraloidea	Crambidae	Spilomelinae	*Palpita hypohomalia* Inoue, 1996	MG869628	C	15271	-0,001	81,029	-0,196	18,971
MIQGO	Pyraloidea	Crambidae	Spilomelinae	*Pycnarmon lactiferalis* (Walker, 1859)	KX426346	C	15219	-0,004	81,681	-0,173	18,319
MIQGO	Pyraloidea	Crambidae	Spilomelinae	*Spoladea recurvalis* (Fabricius, 1775)	KJ739310	C	15273	-0,024	80,894	-0,183	19,106
MIQGO	Pyraloidea	Crambidae	Spilomelinae	*Tyspanodes striata* (Butler, 1879)	KP347977	C	15255	-0,018	81,383	-0,177	18,617

GO	SUPERFAMILY	FAMILY	SUBFAMILY	SPECIES	GenBank	C/P	Size	AT-skew	AT%	GC-skew	GC%
**MIQGO**	Pyraloidea	Pyralidae	Epipaschiinae	*Lista haraldusalis* (Walker, 1859)	KF709449	**C**	15213	-0,007	81,516	-0,171	18,484
**MIQGO**	Pyraloidea	Pyralidae	Galleriinae	*Galleria mellonella* (Linnaues, 1758)	KT750964	**C**	15320	-0,039	80,418	-0,237	19,582
**MIQGO**	Pyraloidea	Pyralidae	Phycitinae	*Amyelois transitella* (Walker, 1863)	KT692987	**C**	15205	-0,048	79,638	-0,237	20,362
**MIQGO**	Pyraloidea	Pyralidae	Phycitinae	*Ephestia elutella* (Hübner, 1796)	MG748858	**C**	15346	-0,043	80,653	-0,217	19,347
**MIQGO**	Pyraloidea	Pyralidae	Phycitinae	*Euzophera pyriella* Yang, 1994	KY825744	**C**	15184	-0,052	79,814	-0,242	20,179
**MIQGO**	Pyraloidea	Pyralidae	Phycitinae	*Meroptera pravella* (Grote, 1878)	MF073207	**C**	15260	-0,019	80,550	-0,199	19,332
**MIQGO**	Pyraloidea	Pyralidae	Phycitinae	*Plodia interpunctella* (Hübner, 1813)	KP729178	**C**	15287	-0,049	80,153	-0,231	19,847
**MIQGO**	Pyraloidea	Pyralidae	Pyralinae	*Hypsopygia regina* (Butler, 1879)	KP327714	**C**	15212	-0,037	78,655	-0,228	21,345
**MIQGO**	Thyridoidea	Thyrididae		*Pyrinioides aurea* Butler, 1881	KT337662	**C**	15362	0,000	79,938	-0,216	20,062
**MIQGO**	Thyridoidea	Thyrididae		*Rhodoneura mellea* (Saalmüller, 1881)	KJ508038	**P**	15615	-0,023	80,666	-0,232	19,167
**MIQGO**	Tortricoidea	Tortricidae	Olethreutinae	*Cydia pomonella* (Linnaeus, 1758)	JX407107	**C**	15253	-0,004	80,128	-0,207	19,872
**MIQGO**	Tortricoidea	Tortricidae	Olethreutinae	*Grapholita dimorpha* Komai, 1979	KJ671625	**C**	15813	-0,011	80,845	-0,189	19,155
**MIQGO**	Tortricoidea	Tortricidae	Olethreutinae	*Retinia pseudotsugaicola* Liu & Wu, 2001	KF498969	**C**	15282	-0,005	79,774	-0,224	20,226
**MIQGO**	Tortricoidea	Tortricidae	Olethreutinae	*Spilonota lechriaspis* Meyrick, 1932	HM204705	**C**	15368	-0,018	81,195	-0,188	18,805
**MIQGO**	Tortricoidea	Tortricidae	Tortricinae	*Acleris fimbriana* (Thunberg & Becklin, 1791)	HQ662522	**C**	15933	-0,012	80,951	-0,169	19,049
**MIQGO**	Tortricoidea	Tortricidae	Tortricinae	*Adoxophyes orana* (Fischer von Röslerstamm, 1834)	JX872403	**C**	15343	-0,001	79,958	-0,211	20,042
**MIQGO**	Tortricoidea	Tortricidae	Tortricinae	*Choristoneura longicellana* (Walsingham, 1900)	HQ452340	**C**	15759	0,010	81,141	-0,193	18,859
**MIQGO**	Urodoidea	Urodidae		*Urodus decens* Meyrick, 1925	KJ508062	**P**	15279	-0,018	80,202	-0,205	19,668
**MIQGO**	Yponomeutoidea	Lyonetiidae		*Leucoptera malifoliella* Costa, 1836	JN790955	**C**	15646	0,016	82,564	-0,199	17,436
**MIQGO**	Yponomeutoidea	Lyonetiidae		*Lyonetia clerkella* Linnaeus, 1758	MF045483	**C**	15259	0,017	79,920	-0,180	20,080
**MIQGO**	Yponomeutoidea	Plutellidae		*Plutella australiana* Landry & Hebert, 2013	MG787473	**C**	15962	0,014	80,673	-0,185	19,327
**MIQGO**	Yponomeutoidea	Yponomeutidae	Praydinae	*Prays oleae* Bernard, 1788	KM874804	**C**	16499	0,019	81,817	-0,179	18,183
**MIQGO**	Zygaenoidea	Limacodidae		*Monema flavescens* Walker, 1855	KU946971	**C**	15396	0,013	80,475	-0,226	19,525
**ParaGO**	Zygaenoidea	Limacodidae		*Parasa consocia* Walker, 1865	KX108765	**C**	15296	-0,001	80,583	-0,238	19,417
**ChalGO**	Zygaenoidea	Zygaenidae	Chalcosiinae	*Eterusia aedea* Clerck, 1759	MH316560	**C**	15196	-0,005	79,606	-0,211	20,394
**ChalGO**	Zygaenoidea	Zygaenidae	Chalcosiinae	*Histia rhodope* (Cramer, 1775)	MF542357	**C**	15209	0,015	78,447	-0,243	21,553
**ChalGO**	Zygaenoidea	Zygaenidae	Chalcosiinae	*Pidorus atratus* Butler, 1877	MG882482	**C**	15383	0,010	79,861	-0,207	20,139
**ChalGO**	Zygaenoidea	Zygaenidae	Chalcosiinae	*Rhodopsona rubiginosa* (Leech, 1898)	KM244668	**C**	15248	0,010	78,922	-0,228	21,078

**C/P**	Complete/Partial genome
**Size**	Genome Size
**AT-skew**	
**AT%**	percentage of A+T
**GC-skew**	
**GC%**	percentage of G+C

Alignment S1

nad2 vs ISP *trnQ-nad2* pairwise alignments in *Lopinga achine* (Nymphalidae, Satyrinae)

Segment (Starting nucleotide-ending nucleotide)

nad2 segment A (18-79)

```
                                                10        20        30        40        50        60
                                                 |         |         |         |         |         |
Lopinga achine new00000 nad2 segment A     TTCTAATAAAATATTTTTTTATTTACTTTATTTTTAGAACTA-TAATTTCAATT-TCTTCTA
Lopinga achine new00000 ISP trnQ-nad2      TTTTAAAAAAAAATGA---TAATTTAAATCATCCCTAAGAAATAAT-ATTTC--TTAT-TTTTA
```

Alignment length: 64; identity: 40 (62.50%); different 24 (37.50 %).

nad2 segment B (233-288)

```
                                                10        20        30        40        50        60
                                                 |         |         |         |         |         |
Lopinga achine new00000 nad2 segment B     TCTTAAGATTATTTTTAATAAA--AAATT-TTTTTATGG-ATAA-ATTCATTTCAATTTTA
Lopinga achine new00000 ISP trnQ-nad2      TTTTAAAAAAAAA--TGATAATTTAAATCATCCCTAAGAAATAATATT--TCTTATTTTTA
```

Alignment length: 61; identity: 35 (57.38%); different: 26 (42.62%)

nad2 segment C (684-738)

```
                                                10        20        30        40        50        60
                                                 |         |         |         |         |         |
Lopinga achine new00000 nad2 segment C     TTTTAATATTAAATTTTCTATT-AAAT--TTTCTATTATAATTAAT-TTTCTT-TCTTTA
Lopinga achine new00000 ISP trnQ-nad2      TTTTAAAAAAAAATGATA-ATTTAAATCATCCCTA--AGAAATAATATTTCTTATTTTTA
```

Alignment length: 60; identity: 41 (68.33%); different: 19 (31.67%).

nad2 segment D (900-955)

```
                                                10        20        30        40        50        60
                                                 |         |         |         |         |         |
Lopinga achine new00000 nad2 segment D     TTTTAAATTTAAATGAT--TTAAAATTTATAT-TAA-AAATAAT-TTT-TTAATTATAATTA
Lopinga achine new00000 ISP trnQ-nad2      TTTTAAAAAAAAATGATAATTTAAATC-ATCCCTAAGAAATAATATTTCTTA-TT-T--TTA
```

Alignment length: 62; identity: 44 (70.97%); different: 18 (29.03%)

Alignment S2

***nad2* vs ISP *trnQ*-*nad2* pairwise alignments in *Parnassius apollo* (Papilionidae, Parnassiinae)**

Segment (Starting nucleotide-ending nucleotide)

nad2 segment A (3-44)

```
                                           10        20        30        40
                                            |         |         |         |
Parnassius apollo new00000 nad2 segment A  TTTTTTTAATTTAAATTCAAATAAAA-TATTTTTTTATTTTAT
Parnassius apollo new00000 ISP trnQ-nad2   TTTATTGAATTTAAAT---AACAAAACTAACCCCCTATTTTAG
```

Alignment length: 43; identity: 29 (67.44%); different: 14 (32.56 %).

nad2 segment B (102-146)

```
                                           10        20        30        40
                                            |         |         |         |
Parnassius apollo new00000 nad2 segment B  TGGATTAGAAATCAACTTATTAAGATTTATCCCCCTAATTTCCAA
Parnassius apollo new00000 ISP trnQ-nad2   TTTATT-GAATTTAAATAAC-AAAAC-TAACCCCCTA-TTTT-AG
```

Alignment length: 45; identity: 28 is (62.22%); different: 17 (37.78 %).

nad2 segment C (249-289)

```
                                           10        20        30        40
                                            |         |         |         |
Parnassius apollo new00000 nad2 segment C  ATTAAAAAATTTTGAAATTAATAAT-TTAATTT-CTATTTTAA
Parnassius apollo new00000 ISP trnQ-nad2   TTTATTGAATTT--AAAT-AACAAAACTAACCCCCTATTTTAG
```

Alignment length: 43; Identity: 27 (62.79%); Different: 16 (37.21%).

nad2 segment D (585-626)

```
                                           10        20        30        40
                                            |         |         |         |
Parnassius apollo new00000 nad2 segment D  TTTAT-GAATTTTTTATTTAATAATA-TA-TTCATTT-TTAATTAG
Parnassius apollo new00000 ISP trnQ-nad2   TTTATTGAATTT---AAATAACAAAACTAACCC-CCTATT--TTAG
```

Alignment length: 46; Identity: 28 (60.87%); Different: 18 (39.13%).

nad2 segment E (718-760)

```
                                           10        20        30        40
                                            |         |         |         |
Parnassius apollo new00000 nad2 segment E  TTAATT-AATTTTTTATCCTTAGGAGGATTACCCCCCT-TTTTAG
Parnassius apollo new00000 ISP trnQ-nad2   TTTATTGAATTTAA-AT----AACAAAACTAACCCCCTATTTTAG
```

Alignment length: 45; Identity: 29 (64.44%); Different: 16 (35.56%).

nad2 segment F (942-983)

```
                                           10        20        30        40
                                            |         |         |         |
Parnassius apollo new00000 nad2 segment F  ATTATT-AATTATAAATATTTCAAGAA-TAATTT-CT-TTATTAG
Parnassius apollo new00000 ISP trnQ-nad2   TTTATTGAATT-TAAATAA--CAA-AACTAACCCCCTATT-TTAG
```

Alignment length: 45; identity: 31 (68.89%); different: 14 (31.11%).

Alignment S3

***nad2* vs ISP *trnQ-nad2* pairwise alignments in *Choaspes benjaminii* (KJ629164) (Hesperiidae, Coliadinae)**

Segment (Starting nucleotide-ending nucleotide)

***nad2* segment A (9-78)**

```
                                                                  10        20        30        40        50        60        70        80
                                                                   |         |         |         |         |         |         |         |
Choaspes benjaminii KJ629164 nad2 segment A                TAATATTAATTCTAATAAA-A-ATTTTT--T---TTT-T--TTTATTTTAATTATAAGAACCCTAATTTCTATTTCTTCA
Choaspes benjaminii KJ629164 ISP trnQ-nad2     6 x (TA) + TAAAAATAATTTTTATAAATATATTTTTAATAAATTTATAATTTATTT--ATTTTAATAA----AAT---TATTT-TT-A
```

Alignment length: 80; Identity: 53 (66.25%); Different: 27 (33.75%).

***nad2* segment B (236-303)**

```
                                                                 10        20        30        40        50        60        70
                                                                   |         |         |         |         |         |         |
Choaspes benjaminii KJ629164 nad2 segment B                TAAAAATAATTTTAATAAAAA-ATTTT--G-AAATTAATAATTTATTTTCAATTTTAATT-AATTCTTCCTTA
Choaspes benjaminii KJ629164 ISP trnQ-nad2     6 x (TA) + TAAAAATAATTTTTATAAATATATTTTTAATAAATTTATAATTTATTT---ATTTTAATAAAATTATTT-TTA
```

Alignment length: 73; identity: 57 (78.08%); different: 16 (21.92%).

***nad2* segment C (572-646)**

```
                                                                  10        20        30        40        50        60        70        80
                                                                   |         |         |         |         |         |         |         |
Choaspes benjaminii KJ629164 nad2 segment C                TAATTAGAGAAAATTTATTTATATTT-TATTTTT--T-AATTTATTCTTT-CTTAATTATAATTATATGTTTTATTTTTC
Choaspes benjaminii KJ629164 ISP trnQ-nad2     6 x (TA) + TAA--A-A-ATAATT--TTTATAAATATATTTTTAATAAATTTATAATTTATTTA-TTTTAATAA-A-A--TTATTTTTA
```

Alignment length: 80; identity: 54 (67.50%); different: 26 (32.50%).

***nad2* segment D (651-733) (Kim et al. 2014, segment)**

```
                                                                  10        20        30        40        50        60        70        80
                                                                   |         |         |         |         |         |         |         |
Choaspes benjaminii KJ629164 nad2 segment D                TATAAATATTTATTTTATAAATCAATTATTTATCTATAATATAAATTTTTTAATTAAATTTATTATATTT-ATTAA--TT-TTCTTT-
Choaspes benjaminii KJ629164 ISP trnQ-nad2     6 x (TA) + TAAAAATAAT--TTTTATAAAT--A-TATTT---T-TA--ATAAATTT----AT--AATTTATT-TATTTTAATAAAATTATT-TTTA
```

Alignment length: 88; identity: 61 (69.32%); different: 27 (30.68%).

***nad2* segment E (917-994)**

```
                                                                  10        20        30        40        50        60        70        80
                                                                   |         |         |         |         |         |         |         |
Choaspes benjaminii KJ629164 nad2 segment E                TTAAAAT--TTTCATTAAAAATA-ATTACTTTATGTTTATTATATTATTTAATTTAATTTCCTTAATAGGAATAATTTTTA
Choaspes benjaminii KJ629164 ISP trnQ-nad2     6 x (TA) + TAAAAATAATTT--TTATAAATATATT--TTTA-ATAAATT-TATAATTTA-TTTA-TT---TTAATAA-AATTATTTTTA
```

Alignment length: 81; identity: 58 (71.60%); different: 23 (28.40%).

6 x (TA), repeated dinucleotide not considered in the alignment

Kim, J.M, Whang, A.R., Park, J.S., Kim, I. Complete mitochondrial genomes of five skippers (Lepidoptera: Hesperiidae) and phylogenetic reconstruction of Lepidoptera. *Gene* **2014**, 549, 97-112.

Alignment S4

nad2 _vs_ ISP _trnQ_-_nad2_ pairwise alignments _in Lobocla bifasciatus_ (KJ629166) (Hesperiidae, Eudaminae)

Segment (Starting nucleotide-ending nucleotide)

nad2 segment A (230-302)

```
                                                                   10        20        30        40        50        60        70
                                                                    |         |         |         |         |         |         |
Lobocla bifasciatus KJ629166 nad2 segment A                   TTTTATTAAAAATAATTTTATTAAAAAATTTTGAAATAAATAATTT--TTTAT-CAATTTTAATTAATTCCTCATT
Lobocla bifasciatus KJ629166 ISP trnQ-nad2    11 x (TA) + TATTAAAAAAAATAATTTAATTT----ATTT--AAATAAATT-TTTAATTTATTCTATTTAAACTA-TT--TT-TT
```

Alignment length: 76; identity: 52 (**68.42%**); different: 24 (31.58%).

nad2 segment B (641-717) (Kim et al. 2014, segment)

```
                                                                  10        20        30        40        50        60        70
                                                                   |         |         |         |         |         |         |
Lobocla bifasciatus KJ629166 nad2 segment B                   TATTTTATATTTTTAATA-TTTATTTTATTAATCAATTATTTATTTTTAATATAAATTCCCTATTAAAATT-TTTTATT
Lobocla bifasciatus KJ629166 ISP trnQ-nad2    11 x (TA) + TATTAAAAAA----AATAATTTAATTTATTTA--AATAA---ATTTTTAATTT--ATT--CTATTTAAACTATTTT-TT
```

Alignment length: 79; identity: 53 (**67.09%**); different: 26 (32.91%).

nad2 segment C (901-972)

```
                                                                   10        20        30        40        50        60        70
                                                                    |         |         |         |         |         |         |
Lobocla bifasciatus KJ629166 nad2 segment C                   TATTTAAAATTAAAATGATTTAAAATATTTATTAAAAATAAATATTTCTCTTTTATTTTATTTAA--TATTTTAATT
Lobocla bifasciatus KJ629166 ISP trnQ-nad2    11 x (TA) + TA-TTAAAA--AAAATAATTTA----ATTTATTT-AAATAAAT-TTT-TAATTTATTCTATTTAAACTATTTT--TT
```

Alignment length: 77; identity: 58 (**75.32%**); different: 19 (24.68%).

11 x (TA), repeated dinucleotide not considered in the alignment

Kim, J.M, Whang, A.R., Park, J.S., Kim, I. Complete mitochondrial genomes of five skippers (Lepidoptera: Hesperiidae) and phylogenetic reconstruction of Lepidoptera. _Gene_ **2014**, 549, 97-112.

Alignment S5

nad2 *vs* ISP *trnQ*-*nad2* pairwise alignments in *Euschemon rafflesia* (KY513288) (Hesperiidae, Euschemoninae)

Segment (Starting nucleotide-ending nucleotide)

nad2 segment A (7-85)

```
                                            10        20        30        40        50        60        70
                                             |         |         |         |         |         |         |
Euschemon rafflesia KY513288 nad2 segment A    TTACTTAATATTAATTCTAACAAAATATTTTTTATTTTTATTTTATTTTTTAGAACCCTTATTTCAATCTCAGCTAATT
Euschemon rafflesia KY513288 ISP trnQ-nad2     CTA-TTAAAATAAATT-----AAATTAATTTTT-TCATTATATAAATTTT-AAA---TTTATATTATTTTTAATTATTT
```

Alignment length: 79; identity: 48 (60.76%); different: 31 (39.24%).

nad2 segment B (179-250)

```
                                            10        20        30        40        50        60        70
                                             |         |         |         |         |         |         |
Euschemon rafflesia KY513288 nad2 segment B    CT-TTAAAATATTTTTTAACTCAATCTATTGCC-TCTAT-TAATTTTTTATTTTCTATT-TTATTGAAAATAATTT
Euschemon rafflesia KY513288 ISP trnQ-nad2     CTATTAAAATAAA--TTAAATTAATTT-TT-TCAT-TATATAAATTTTAAATTTATATTATT-TTT--AATTATTT
```

Alignment length: 76; identity: 52 (68.42%); different: 24 (31.58%).

nad2 segment C (391-466)

```
                                            10        20        30        40        50        60        70        80
                                             |         |         |         |         |         |         |         |
Euschemon rafflesia KY513288 nad2 segment C    -TCTTGACAAAAAATTACCCCTATAATTTTATTGTCATATTATATAAATAA-AAATTT-T-TTATTATTAATTATAATTA
Euschemon rafflesia KY513288 ISP trnQ-nad2     CTATTAA-AATAAATTAAAT---TAATTTT-TT----CATTATATAAATTTTAAATTTATATTATTTTTAATTAT--TT-
```

Alignment length: 80; identity: 54 (67.50%); different: 26 (32.50%).

nad2 segment D (572-645)

```
                                            10        20        30        40        50        60        70
                                             |         |         |         |         |         |         |
Euschemon rafflesia KY513288 nad2 segment D    TAATTAGAGAAAATTTATGATTATTATATTTTATTTTATATTCTTTTTTAATTAGAGTTATATGCTTATTATTT
Euschemon rafflesia KY513288 ISP trnQ-nad2     CTATTAAAATAAATTAA--ATTA--AT-TTTTTCATTATATAAATTTTAAATTTATATTAT-TTTTAATTATTT
```

Alignment length: 74; identity: 48 (64.86%); different: 26 (35.14%).

nad2 segment E (650-723)

```
                                            10        20        30        40        50        60        70
                                             |         |         |         |         |         |         |
Euschemon rafflesia KY513288 nad2 segment E    TTATAAATATTTTTTTTATTAATCAATTATTTATTTTTAATATAAATTTTATATTAAAATTATTTTTAAT-ATTT
Euschemon rafflesia KY513288 ISP trnQ-nad2     CTATTAAAATAAATTAAATTAAT---TTTTTCATT----ATATAAATTTTAAATTTATATTATTTTTAATTATTT
```

Alignment length: 75; identity: 54 (72.00%); different: 21 (28.00%).

nad2 segment F (830-902)

```
                                            10        20        30        40        50        60        70
                                             |         |         |         |         |         |         |
Euschemon rafflesia KY513288 nad2 segment E    TTATTATTATAAGCTTAATCACATTATTTTTTTATATTCGAATTATTTACTCTTGTATCATATTTAATTATTT
Euschemon rafflesia KY513288 ISP trnQ-nad2     CTATTAAAATAAATTAAATTA-ATTTTTTCATTATAT--AAAT--TTTAAATTTATATTATTTTTAATTATTT
```

Alignment length: 73; identity: 51 (69.86%); Different: 22 (30.14%).

Alignment S6

nad2 *vs* ISP *trnQ*-*nad2* pairwise alignments in *Potanthus flavus* (KJ629167) (Hesperiidae, Hesperiinae)

Segment (**Starting nucleotide**-**ending nucleotide**)

nad2 segment A (227-288)

```
                                                   10        20        30        40        50        60
                                                    |         |         |         |         |         |
Potanthus flavus KJ629167 nad2 segment A   CTATTTTAATTAATTTATTATTTATAAA--AAATTTTGAAATT--AATTT-TT-TAATATCAATCTTA
Potanthus flavus KJ629167 ISP trnQ-nad2    TTATAAAAATAAATTAATTAT--ATAAATTAAATTT-AAAATTTTACTTTATTATAAT-TTATTTTTA
```

Alignment length: 68; identity: 47 (**69.12%**); different: 21 (30.88 %).

nad2 segment B (559-622)

```
                                                   10        20        30        40        50        60        70
                                                    |         |         |         |         |         |         |
Potanthus flavus KJ629167 nad2 segment B   TTATCTTCAATTATAATTAG--AGA-AAATTTATTTTTAA--TTTATTTTATATTATA-TTCT-TTTATTA
Potanthus flavus KJ629167 ISP trnQ-nad2    TTATAA--AAATA-AATTAATTATATAAATTAAATTTAAAATTTTACTTTA--TTATAATT-TATTT-TTA
```

Alignment length: 71; identity: 48 (**67.61%**); different: 23 (32.39 %).

nad2 segment C (665-738) (Kim et al. 2014, segment)

```
                                                        10        20        30        40        50        60        70
                                                         |         |         |         |         |         |         |
Potanthus flavus KJ629167 nad2 segment C   TTATTAATCAATTATTTATTTTTAATATAAATTATATAATTAAAATATT--TTTATTGATTAATTTTCTTTCTTTA
Potanthus flavus KJ629167 ISP trnQ-nad2    TTATAAA--AATAAATTAA--TT-ATATAAATTAAAT--TTAAAATTTTACTTTATT-A-TAATTTA-TTT--TTA
```

Alignment length: 76; identity: 55 (**72.37%**); different: 21 (27.63 %).

nad2 segment D (897-976)

```
                                                   10        20        30        40        50        60        70        80
                                                    |         |         |         |         |         |         |         |
Potanthus flavus KJ629167 nad2 segment D   TTATATTAAATTAAAATGATTTAATGTATTTATTAAAAATAATTATTTTCATCTTATTATAATTTTTAGAATAATTTCTA
Potanthus flavus KJ629167 ISP trnQ-nad2    TTATA--AAAATAAATTAATT--ATATA--AATTAAATTTAAA-ATTTT-ACTTTATTATAATTT-----AT--TTT-TA
```

Alignment length: 80; identity: 54 (**67.50%**); different: 26 (32.50%).

Kim, J.M, Whang, A.R., Park, J.S., Kim, I. Complete mitochondrial genomes of five skippers (Lepidoptera: Hesperiidae) and phylogenetic reconstruction of Lepidoptera. *Gene* **2014**, 549, 97-112.

Alignment S7

***nad2* vs ISP *trnQ*-*nad2* pairwise alignments in *Carterocephalus silvicola* (KJ629163) (Hesperiidae, Heteropterinae)**

Segment (Starting nucleotide-ending nucleotide)

nad2 segment A (17-85)

```
                                                           10        20        30        40        50        60        70
                                                            |         |         |         |         |         |         |
Carterocephalus silvicola KJ629163 nad2 segment A  ACTCTAATAAAATATTTTTTTT-TTTTATTTTAATTTTTAGTACTTTAA-TCTC-AATCTCAGCTAATTCTT
Carterocephalus silvicola KJ629163 ISP trnQ-nad2   GCCC-A--AAAATAATTTATTTACTTTAGTATAAA-TTTA-TAATTTAAATTTAAAAT-TAAATTATTTTTT
```

```
Alignment length: 72; identity: 47 (65.28%); different: 25 (34.72%).
```

nad2 segment B (139-208)

```
                                                           10        20        30        40        50        60        70
                                                            |         |         |         |         |         |         |
Carterocephalus silvicola KJ629163 nad2 segment B  ATTAATAATTGTAATAATTTATTAACTTCAG-A-AGCATCTT-TAA---AATATTTTCTAGTTCAATCTATTGCTT
Carterocephalus silvicola KJ629163 ISP trnQ-nad2   GCCCA-AA----AATAATTTATTTACTTTAGTATAA-AT-TTATAATTTAA-ATTTAA-AATTAAAT-TATTTTTT
```

```
Alignment length: 76; identity: 47 (61.84%); different: 29 (38.16%).
```

nad2 segment C (233-298)

```
                                                           10        20        30        40        50        60        70
                                                            |         |         |         |         |         |         |
Carterocephalus silvicola KJ629163 nad2 segment C  TCATTAAAAATAATTT---TA--TTAA-A-AAATTTTGAAATGAATTT---TTTACTATCAATTTTAATCAATTCCT
Carterocephalus silvicola KJ629163 ISP trnQ-nad2   GC-CCAAAAATAATTTATTTACTTTAGTATAAATTT----AT-AATTTAAATTTA--A--AATTA-AATTATTTTTT
```

```
Alignment length: 77; identity: 47 (61.04%); different: 30 (38.96%).
```

nad2 segment D (408-480) (Kim et al. 2014, segment)

```
                                                           10        20        30        40        50        60        70
                                                            |         |         |         |         |         |         |
Carterocephalus silvicola KJ629163 nad2 segment D  -CCCCATAAT--TTTATTGTCCTATTACTTAAATAAAAATTTTTTAATATTAA-TTATAATAACTAATACAATTATT-
Carterocephalus silvicola KJ629163 ISP trnQ-nad2   GCCCAAAAATAATTTATT----TACT--TTAG-TATAAATTTA-TAAT-TTAAATT-TAA-AATTAA-ATTATTTTTT
```

```
Alignment length: 78; identity: 51 (65.38%); different: 27 (34.62%).
```

nad2 segment E (927-994)

```
                                                           10        20        30        40        50        60        70
                                                            |         |         |         |         |         |         |
Carterocephalus silvicola KJ629163 nad2 segment E  TATT-AAAAATAATT-ATTTTTCTTTTGTATT-ATTTAATAGCTTAA-TTTCAATTTTAGGAATAATTCTTA
Carterocephalus silvicola KJ629163 ISP trnQ-nad2   -GCCCAAAAATAATTTATTTA-CTTTAGTATAAATTTA-TAATTTAAATTTAAAA-TTA--AATTATTTTTT
```

```
Alignment length: 72; Identity: 49 (68.06%); Different: 23 (31.94%).
```


Kim, J.M, Whang, A.R., Park, J.S., Kim, I. Complete mitochondrial genomes of five skippers (Lepidoptera: Hesperiidae) and phylogenetic reconstruction of Lepidoptera. *Gene* **2014**, 549, 97-112.

Alignment S8

***nad2* vs ISP *trnQ*-*nad2* pairwise alignments in *Daimio tethys* (KJ629165) (Hesperiidae, Tagiadinae)**

Segment (Starting nucleotide-ending nucleotide)

nad2 segment A (17-86)

```
                                                                    10         20         30         40         50         60         70
                                                                     |          |          |          |          |          |          |
Daimio tethys KJ629165 nad2 segment-A                     ACTCAAATAAAATATTTTTTTTTTTTAT-ATTAACTTTTAGTACTTTTATCTCTATTTCATCAAA----TTCATG
Daimio tethys KJ629165 ISP trnQ-nad2     7 x (TA) + TCTCATA-AAAATATTTTTTTAGTTAAAAAATAAGTTT-A-TAATTT-AT-T-T-TTTTATTAAAAATATTTTTA
```

Alignment length: 75; identity: 49 (**65.33%**); different: 26 (34.67%).

nad2 segment B (173-250)

```
                                                                    10         20         30         40         50         60         70         80
                                                                     |          |          |          |          |          |          |          |
Daimio tethys KJ629165 nad2 segment B                     CCTCTTTAAAATATTTTTT-AACTCAATCAATT-GCTTCAATTAATTTCTTATTTTCTATCTTATTAAAAATAATTTTCA
Daimio tethys KJ629165 ISP trnQ-nad2     7 x (TA) + TCTCATAAAAATATTTTTTTAG-TTAAAAAATAAGTTT--A-TAAT---TTATTTT---T-TTATTAAAAATA-TTTTTA
```

Alignment length: 80; identity: 56 (**70.00%**); different: 24 (30.00%).

nad2 segment C (408-478)

```
                                                                    10         20         30         40         50         60         70
                                                                     |          |          |          |          |          |          |
Daimio tethys KJ629165 nad2 segment C                     CCCCATAATTTTATTGTCTTATTATTTAAATACA-AATTTTTTAATT-ATTATTATAATTATTAA---TACTAGCA
Daimio tethys KJ629165 ISP trnQ-nad2     7 x (TA) + TCTCATAAAAATATT-T-TT-TTAGTTAAAAA-ATAAGTTTATAATTTATT-TT-T--TTATTAAAAATATTTTTA
```

Alignment length: 76; identity: 50 (**65.79%**); different: 26 (34.21%).

nad2 segment D (681-759) (Kim et al. 2014, segment)

```
                                                                    10         20         30         40         50         60         70         80
                                                                     |          |          |          |          |          |          |          |
Daimio tethys KJ629165 nad2 segment D                     TAT-ATTTAATATAA-ATTTCTT-A-TTCAAATTTT--TTCTATTAATAAATTTTTTATCTTTGGGAGGATTACCCCCTCTTTTA
Daimio tethys KJ629165 ISP trnQ-nad2     7 x (TA) + TCTCA--TAA-A-AATATTTTTTTAGTTAAAAAATAAGTT-TA-TAATTTATTTTTT-TATT---AAAAAT-A-----T-TTTTA
```

Alignment length: 85; identity: 50 (**58.82%**) different: 35 (41.18%).

nad2 segment E (808-880)

```
                                                                    10         20         30         40         50         60         70
                                                                     |          |          |          |          |          |          |
Daimio tethys KJ629165 nad2 segment E                     TTTC-TAAT--TACTTCTTTTATTTTTATTATAATAAGTTTAATTACTTTATTTTTTTATATTCGAATTATTTATTC
Daimio tethys KJ629165 ISP trnQ-nad2     7 x (TA) + TCTCATAAAAATATTT-TTTTAG--TTAA-AAAATAAGTTTA--TAATTTATTTTTTTAT--TAAAAATATTT-TTA
```

Alignment length: 77; identity: 54 (**70.13%**); different: 23 (29.87%).

The repeated element (TA)$_7$ was not included in the alignment

Kim, J.M, Whang, A.R., Park, J.S., Kim, I. Complete mitochondrial genomes of five skippers (Lepidoptera: Hesperiidae) and phylogenetic reconstruction of Lepidoptera. *Gene* **2014**, 549, 97-112.

Alignment S9

Multiple alignment of ***trnMs*** and ISPs ***trnQ*-*nad2*** in selected species of the family Hesperiidae.

Hesperiidae

Sequence	Alignment (positions 10, 20, 30, 40, 50, 60, 70, 80, 90 marked)	Subfamily
***Daimio tethys* trnM**	`-------TTA---AAAATAAGCT---------AA-T-TA-AAGCTTTTG-GGCT-CATACCTCAAATATAAAG--GA---ATTCCCTTTTTTTTAAA`	Tagiadinae
***Pyrgus maculatus* trnM**	`-------TTA---AAAATAAGCT---------AAATA-A-A-GCTTTTG-GGTT-CATACCCCAAATATAAAG--GA---ATACCCTTTTTTTTAAA`	Pyrginae
***Rachelia extrusus* trnM**	`-------TTA---AAAATAAGCT---------AATT-TA-A-GCTTTTG-GGTT-CATACCCCAAATATATAG--GAT--ATAC-CTTTTTTTTAAA`	Trapezitinae
***Barca bicolor* trnM**	`-------TTA---AAAATAAGCT---------AA-TA-A-AAGCTTTTG-GGTT-CATACCTCAAATATAAA---GAC--ATTCTCTTTTTTTTAAA`	Barcinae
***Malaza carmides* trnM**	`-------TTA---AAAATAAGCT---------AAAT-TA-A-GCTTTTG-GGTT-CATACCCCAAATATAGAG--GA---ATACCCTTTTTTTTAAA`	Malazinae
***Potanthus flavus* trnM**	`-------TTA---AAAATAAGCT---------AAAT-TA-A-GCTTTTG-GGTT-CATACCCCAAATATAAAG--GA---ATAACCTTTTTTTTAAA`	Hesperiinae
Daimio tethys* ISP *trnQ*-*nad2	`TATCTCA-TA---AAAATA-TTTTTTTAGTTAAAA-A-ATAAG-TTT-ATAATT---TATTTT---TTTAT---TAA---A-A--AT-ATTTTTA--`	
***Pyrgus maculatus* ISP trnQ-nad2**	`TA-CATA-TAA-AAAAATAA-TTC--------AAATATAT-TACTAT-A-AATTTTA-AATTTAAATGTAAAAATGA---A-A---T-ATTTTTT--`	
Barca bicolor* ISP *trnQ*-*nad2	`--------TAA-GAAAATAAATTTTT-A---AAAATATTTATAAT-T-A-AATT-TATAATTTTAATGTATAA-T-AC--A-ACT-TTATTTTTT--`	
Rachelia extrusus* ISP *trnQ*-*nad2	`--------TA---AAAATAATTTTTTTA---AAAATA-ATAAA-TTT-AAAATT--ATAATTTAAATTTACA--TAA---A-A--ATTATTTTTA--`	
Malaza carmides* ISP *trnQ*-*nad2	`---TTTATTAATAAAAATAA-TTT--------AATTAAAT-AA-TAT-A-AATT-TAAAATTTTAATTTAT---T-ACGCA-A--ATTATTTTTT--`	
***Potanthus flavus* ISP trnQ-nad2**	`-------TTA-TAAAAATAAATT---------AATTATATAAA-T-T-A-AATT-TAAAATTTTACTTTAT---T-AT--A-A-T-TTATTTTTA--`	

N, fully conserved nucleotide; N, partly conserved nucleotide;
N, nucleotide peculiar for *trnM*; N, nucleotide peculiar for **ISP trnQ**-*nad2*
N, position where the alignment cannot be done with certainty.

Alignment S10

Multiple alignment of ***trnMs*** and ISPs ***trnQ-nad2*** in selected species of the families Lycaenidae and Riodinidae.

Lycaenidae + Riodinidae

```
                                           10        20        30        40        50        60        70
                                            |         |         |         |         |         |         |
Heliophorus eventa trnM            ----TTAAAAATAAGCTAAATTAAAGCTTTTGGGTTCA-TACCCCAAATATAA--AGGAAAC-CCTT-TTTTTTAAA   Lycaeninae
Lycaena li trnM                    ----TTAAAAATAAGCTAAATTTAAGCTTTTGGGTTCA-TACCCCAAATATAA--AGAAAACT-CTT-TTTTTTAAA
Ahlbergia circe trnM               ----TTAAAAATAAGCTAAA-TAAAGCTTTTGGGTTCA-TACCCCAAATATAA--AGGAAATACCTT-TTTTTTAAA   Theclinae
Favonius orientalis trnM           ----TTAAAAATAAGCTAAACTAA-GCTTTTGGGTTCA-TACCCCAAATATAA--AGGAAACCCCTT-TTTTTTAAA
Aricia agestis trnM                ----TTAAAAATAAGCTAAA-TAAAGCTTTTGGGTTCA-TACCCCAAATATAA--AGGAAATACCTT-TTTTTTAAA   Polyommatinae
Celastrina argiolus trnM           ----TTAAAAATAAGCTAAA-TAAAGCTTTTGGGTTCA-TACCCCAAATATAA--AGGAAATGCCTT-TTTTTTAAA
Apodemia mormo trnM                ----TTAAAAATAAGCTAAA-TTAAGCTTTTGGGCTCA-TACCTCAACTATAA--AGGAAAT-CCTT-TTTTTTAAA   Riodinidae
Dodona_durga trnM                   ---TTAAAAATAAGCTAAA-TAAAGCTTTTGGGTTCA-TACCCCAAATATAA--AGAAAA-ACCTT-TTTTTTAAA
Heliophorus eventa ISP trnQ-nad2   TTT-TTAAA--TAA---AAA-TAAA-CTTATA-GTTTA-TA-----AATG-AA-TATT-AATT-CTT-TTTTAC---
Lycaena li ISP trnQ-nad2           CT--TT-----TAA---AAA-TAAA-TTTATA-ATTTA-T-T----AA-G-AA-TACT-AATTTCTA-TTTTAT---
Ahlbergia circe ISP trnQ-nad2      TTTATTAAA--TAA---AAT-TAAA-TTTTTA-ATTTAAT-T----A-TGAAA-TA---ATTTTCTT-TTTTAT---
Favonius orientalis ISP trnQ-nad2  -TT-TTACTA-TAA---AAA-TAAA-TTTATA-ATTTA-TA-----AATG-AATTA-A-AATT-CTT-TTTTAT---
Aricia agestis ISP trnQ-nad2       CTT-TTAA---TAA---AAA-TAAA-CTTACA-GTTTA-TATT-AGAAT--AATT-TT-ATTTTCTT-TTTTAT---
Celastrina argiolus ISP trnQ-nad2  TTTTTTA----TAA---AAA-TAAA-CTTTTA-ATTTA-T-T-C--AA-G-AA-TA-ACA-TT-CTT-TTTTTT---
Dodona_durga ISP trnQ-nad2          TTTTTA----TAA---AAA--AAA-C-TG-A-ATTTAAAATTCTTATT--AG--ATT-AATTTCTTATATTTT---
Apodemia_mormo trnQ-nad2            ----TA----CAA---ATA-TAAAGAATT----TTTA--ATTCT-ACTA-AG--ATTAAATT-CTTATTTGAT---
```

N, fully conserved nucleotide; N, partly conserved nucleotide;
N, nucleotide peculiar for *trnM*; N, nucleotide peculiar for **ISP** ***trnQ-nad2***
N, position where the alignment cannot be done with certainty.

Alignment S11

Multiple alignment of *trnMs* and ISPs *trnQ-nad2* in selected species of the subfamily Pierinae.

Pierinae

```
                                          10        20        30        40        50        60        70
                                           |         |         |         |         |         |         |
Anthocharis cardamines trnM          -------TTAAGAATA-AGCTAAATTAAGCTTTTGGGCTCATACCTCAAATATAAAGA-TATTTCTTTTTCTTAAT
Baltia butleri trnM                  -------TTAAGAATA-AGCTAAGTTAAGCTTTTGGGTTCATACCTCAAATATAAAGA-TATC-CTTTTTCTTAAA
Pontia callidice trnM                -------TTAAGAATA-AGCTAAGTTAAGCTTTTGGGCTCATACCTCAAATATAAAGA-TATC-CCTTTTCTTAAT
Talbotia_nagana trnM                 -------TTAAGAATA-AGCTAAATTAAGCTTTTGGGCTCATACCTCAAATATAAAGAATA-CCCTTTTTCTTAAA
Appias lyncida trnM                  -------TTAAGAATA-AGCTAAGTTAAGCTTTTGGGCTCATACCTCAAATATAAAGA-TATC-CTTTTTCTTAAT
Pieris brassicae trnM                -------TTAAGAATA-AGCTAAATTAAGCTATTGGGCTCATACCCCAAATATAAAGA-TAT-ACTTTTTCTTAAA
Aporia crataegi trnM                 -------TTAAGAATA-AGCTAAATTAAGCTTTTGGGCTCATACCTCAAATATAAAGA-T-CCTCTTTTTCTTAAT
Delias hyparete trnM                 -------TTAAGAATA-AGCTAAGTTAAGCTTTTGGGCTCATACCTCAAATATAAAGA-T-TC-CTTTTTCTTAAT
Anthocharis cardamines ISP trnQ-nad2 -TT--TTATAAAAAAAATG--AATTTAAA--TTT---CT--TA-AT--AATATATTAA-TA----TTTATTTTTA-
Baltia butleri ISP trnQ-nad2         -TT--TTTTAA-AA-ACTG--AACTTAAA--TTT---CT--T-T-T--AAT-TA-----T-T---TATATTTTA-T
Pontia callidice ISP trnQ-nad2       -TT-TTTC-AAAAA-ACTG--AACTTAAA--TTT---CT--T-T-TT--AT-TA-----T-----TTTATTTTA-T
Talbotia nagana ISP trnQ-nad2        -TT--TTCTAAAA-TA--G--AACTTATA--ATT---CT--TAC----AAT-TAC--A-T-----TTTATTTTA-T
Appias lyncida ISP trnQ-nad2         ---------AA-AA-A--G--AATTTAAA--TTT---CTTTTA----AAATA-AT----TAT---TTTATTTTAA-
Aporia crataegi ISP trnQ-nad2        TTTA-TTTTAATAA-A-AG--AATTTAAA--TTT---CT--TTCAT--AAT-T------T-----TTTATTTTAA-
Delias hyparete ISP trnQ-nad2        -TTACCCCTAA-A-----G--AACTTAAA--TTT---CT---------AATATAAA-C-TA----TTTATTTTAA-
Pieris brassicae ISP trnQ-nad2       ------TTTAATAA-ATAG--AACTTAAA--TTT---CTTTTA----AAAT-T------T-----TTTATTTTAA-
```

N, fully conserved nucleotide; N, partly conserved nucleotide;
N, nucleotide peculiar for *trnM*; N, nucleotide peculiar for **ISP** ***trnQ-nad2***
N, position where the alignment cannot be done with certainty.

Alignment S12

Multiple alignment of *trnMs* and ISPs *trnQ*-*nad2* in selected species of the subfamily Satyrinae (Nymphalidae).

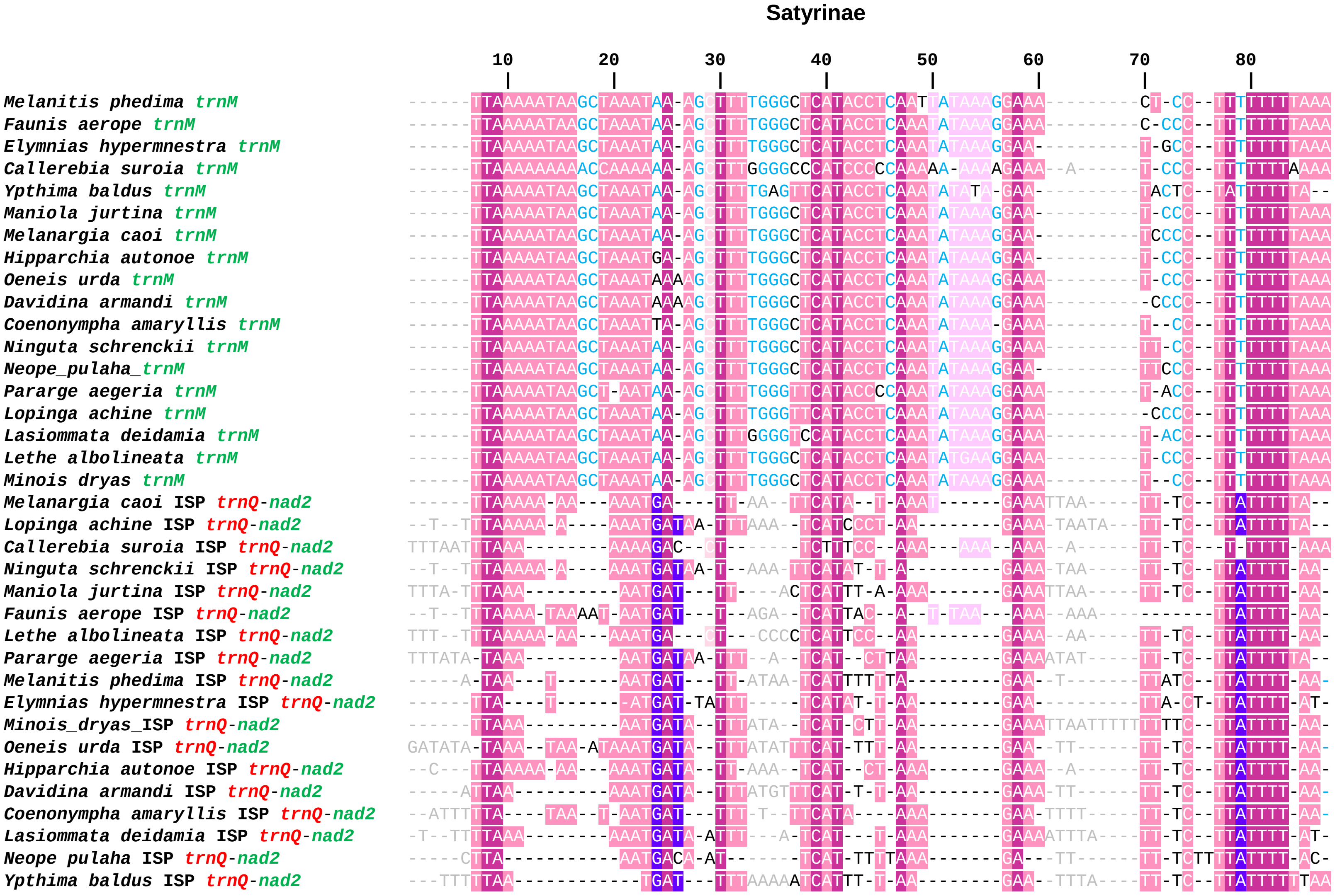

N, fully conserved nucleotide; N, partly conserved nucleotide;
N, nucleotide peculiar for *trnM*; N, nucleotide peculiar for **ISP** *trnQ*-*nad2*
N, position where the alignment cannot be done with certainty.

Alignment S13

Pemphigidae (Hemiptera)

trnM *vs* **ISP** *trnQ*-*nad2* alignment

```
                                            10        20        30        40        50        60        70        80        90
                                             |         |         |         |         |         |         |         |         |
Meitanaphis elongallis trnM           ----------------AGAAAAA-TAAGCTAAATTTAAGCTTT--TGGA-TTCATAC-TTCAAATA-TAG-ATTAAAA-TCTTTTTTCTA
Nurudea ibofushi trnM                 ----------------AGAAAAA-TAAGCTAAATTTAAGCTTT--TGGA-TTCATAC-TTCAAATA-TAGAATTAAAAATCTTTTTTCTA
Schlechtendalia peitan trnM           ----------------AGAAAAA-TAAGCTAAATATAAGCTTT--TGGA-TTCATAC-TTCAAATA-TAGAATAATAAATCTTTTTTCTA
Floraphis choui trnM                  ----------------AGAAAAA-TAAGCTAAATTTAAGCTTT--TGGA-TTCATAC-TTCAAATA-TAGAAATATAAATCTTTTTTCTA
Melaphis rhois trnM                   ----------------AGAAAAA-TAAGCTAAATTCAAGCTTT--TGGA-TTCATAC-TTCAAATA-TAGAACAACAAATCTTTTTTCTA
Melaphis rhois ISP trnQ-nad2          --AAT------ATCTAAGAAAAAATAAACTATAT--AAGTTTT--TGAA-ATTAAAAATTCAATTT-TA-AAAAAAAA----TTTTTCTA
Floraphis choui ISP trnQ-nad2         TAAAT---AATATAT-AGAAAAA-TAAACTA-AT--AAG-TT-AA-GAAAATTTTAAATTCAATTA-TA--ATAAAAA----TTTTTCTA
Schlechtendalia peitan ISP trnQ-nad2  --AAT---AA-ATTT-AGAAAAA-TAAACTT-AT--AAG-TTTA-TGAA-ATTA-AAATTCAAGAAATA--ATATAAA----TTTTTCTA
Nurudea_ibofushi ISP trnQ-nad2        AAAATCTTAAATTAT-AGAAAAA-TAAACTATAT--AAG-TTCA-TGAA-ATT-TAAATTCATATA-TA--AACAAAT----TTTTTCTA
Meitanaphis elongallis ISP trnQ-nad2  ---AT---------TAAGAAAAA-TAAATTAATT--AAAATTTAATGAA-ATTAAAA-TTCAATTA-TA-AAATAAAA----TTTTTCTA
```

trnI *vs* **ISP** *trnM*-*trnQ* alignment

```
                                            10        20        30        40        50        60        70        80        90        100
                                             |         |         |         |         |         |         |         |         |         |
Floraphis_choui ISP trnM-trnQ         -----------------------------------TAAA-CAA---ATTA---TT---TAA--TAAA----------------------------------
Meitanaphis_elongallis ISP trnM-trnQ  ATTAACTTATTTATTTATAAATATT-AAATTATTATAAA-AAATAAATTAAACTTAAATAA--TAAAATTAAAATTAATT-AAATTTAATTAATTT-----
Schlechtendalia peitan ISP trnM-trnQ  ---------------------------AAA---CCTACTTAAA---ATTAAA-TT---TTA--TATAAC--AAA--AT-T-AA------------------
Melaphis rhois ISP trnM-trnQ          -------------------ATAAATTAAAAT-TTATAAATCAATC--TTAAA-TT---TAAATTATAA---AAATTATTTTAAT-TAATTTATTTTTA--A
Nurudea_ibofushi ISP trnM-trnQ        -----------------------------A------G--TA--T--AT-AAATTT---TAA--TATT-T--ATATAAT---AAT--AA-TTA--------A
Nurudea ibofushi trnI                 ----------------------AAT-AAAATG-CCTGATTAAA-GGATTA--CTT---TGA--TAGAGT--AAATAATGTAAAT--ATATTACTTTTATTA
Schlechtendalia peitan trnI           ----------------------AAT-AAAATG-CCTGAAAAAA-GGATTA--CTT---TGA--TAGAGT--AAAACATGT-AAT-TAAA-TACTTTTATTA
Melaphis rhois trnI                   ----------------------AAT-AAAATG-CCTGAA-AAA-GGATTA--CTT---TGA--TAGAGT--AAATTATGTAAAT-TAAATTACTTTTATTA
Meitanaphis elongallis trnI           ----------------------AAT-AAAATG-CCTGAA-AAA-GGATTA--CTT---TGA--TAGAGT--AAATCATGT-AAATTAAAATACTTTTATTA
Floraphis_choui_trnI                  ----------------------AAT-AAAATG-CCTGAA-AAA-GGATTA--CTT---TGA--TAGAGT--AAATCATGTAAAATTAA-TTACTTTTATTA
```

Alignment S14

Curculionidae (Coleoptera)

trnM *vs* **ISP** ***trnQ*-*nad2*** alignment

```
                                                10        20        30        40        50        60        70        80
                                                 |         |         |         |         |         |         |         |
Trypodendron domesticum ISP trnQ-nad2 3'p   -TTATAAA-TTATGCCTCATCTACCT--CTATTGAAGTTATAGTTCAATCTA-AAA-------------------------
Trypodendron signatum ISP trnQ-nad2         AAAATAAAGA-AAGG-T-AGCTACCTTTCTCTTGAAATTATAATTCAA-CTA-AAAAAT-T---T---TTTAATAATTAAT
Trypodendron signatum trnM                  --AA-AAAGATAAG-CT-AA-TAAAG--CTTTTGGGTTCATACCCCAA-CCACAAAGGTGTCACTCC-TTTTCTTTTTA--
Trypodendron domesticum trnM                --AA-AAAGGTAAG-CT-AA-TAAAG--CTTTTGGGTTCATACCCCAA-CCATAAGAGTTTCAACCCCTTT-CTTTTTA--
```

CoRe 3'p + ***trnI*** *vs* **ISP *trnM*-*trnQ* 5'p** alignment

```
                                                     10        20        30        40        50        60
                                                      |         |         |         |         |         |
Trypodendron signatum CoRe 3'p + trnI          ATTTAAAATTTAATATTATAATTTTA-TACAAAAAATAAATTTTACCAAA-ACTA-AAAA
Trypodendron signatum ISP trnM-trnQ 5'p        ATTTAAAACCAAATCTTTTACCCTTAATTTT-AAAATAA-TTTTA--AATTA-TATAAAA

                                                     70        80        90       100       110       120
                                                      |         |         |         |         |         |
Trypodendron signatum CoRe 3'p + trnI          C--TCAATAAATTTAAATAAAATAATTTCAATTAAAACTACAAATTATATGTTTAAAAAA
Trypodendron signatum ISP trnM-trnQ 5'p        CTCTCAATAAA----AATAAACTA-TTT-ATTTAAAAC-GTGAA--AT-C-TTTAAAAAA

                                                    130       140       150       160       170       180
                                                      |         |         |         |         |         |
Trypodendron signatum CoRe 3'p + trnI          TT-TTACCTTTTAAAAA-AATTTACA--A--A-AT-A--ATTCT-TAATTTTAATT--TT
Trypodendron signatum ISP trnM-trnQ 5'p        TTGTT---TTTTAAGTGCAACC-ACACCACCACGTTATTATTCAGCAATTT-AATCCCTT

                                                    190       200       210       220       230       240
                                                      |         |         |         |         |         |
                                                       <-------------------- trnI --------------------
Trypodendron signatum CoRe 3'p + trnI          AAAAATTAAATTAAATATGATGCCTGATAAAA-GGAATATTTTGATAGAATATTACATGG
Trypodendron signatum ISP trnM-trnQ 5'p        ACAAAT-AAGTT--TTATTATA-CTAA-AAAATGG---ATTTTGA---AA-ATT--AT--

                                                    250       260
                                                      |         |
                                               ----------------------->
Trypodendron signatum CoRe 3'p + trnI          AAATCCTAATTTCCTCATA-TTA
Trypodendron signatum ISP trnM-trnQ 5'p        ---T--TATTTTCCTTTTAGCTA
```

Trypodendron signatum: pairwise alignment of a genomic portion (3' end of Control Region + *trnI*) and the first 221 bases of the **ISP *trnM*-*trnQ***

Alignment S15

Multiple alignment of *trnS1* sequences of selected species of Lepidoptera

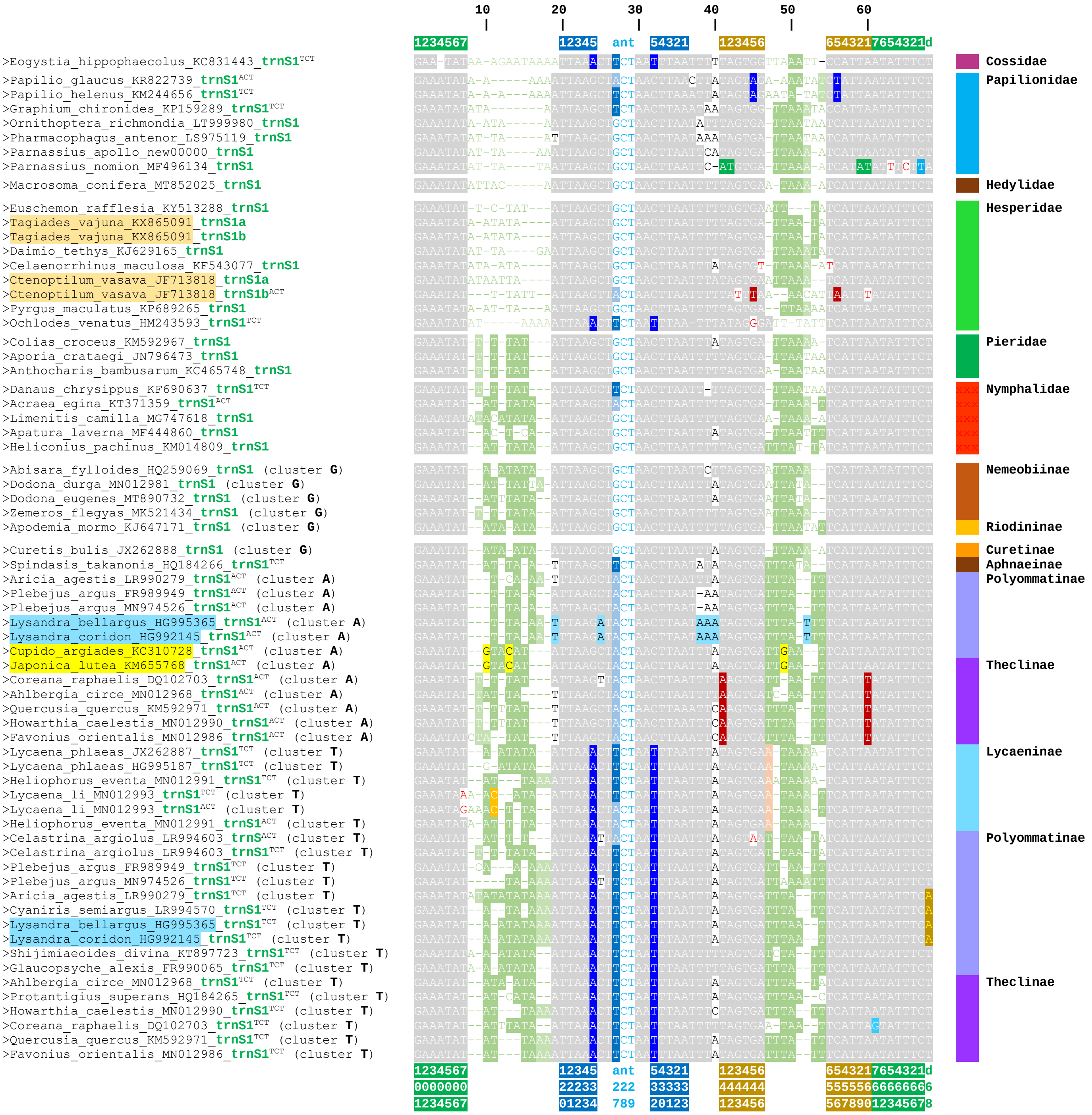

Legend

N, the background base for the position.
N, half-compensatory base change in the stem pair (e.g. T – G vs C – G; A-T vs G-T).
N, N half-compensatory base change in the stem pair exhibiting a mismatch (e.g. T-A vs A-A).
N, N, fully compensatory base change in the stem pair exhibiting a mismatch (e.g. C-G vs T-T).
N, N, type I fully compensatory base change in the stem pair (i.e. purine – pyrimidine vs purine – pyrimidine, e.g. G – C vs A – T).
N, N, type II fully compensatory base change in the stem pair (i.e. purine – pyrimidine vs pyrimidine – purine, e.g. A – T vs T – A).
Different colors are used to better differentiate the changes.
N, a mismatch in the in the stem pair; N, a mismatch in a pair not always fully consistent (e.g. 5 5).
N, substitution pattern not modelled.
*, position in which a mismatch is prominent.
N, N, molecular signature for a taxon.
X, position 1-7 in the acceptor stem; X position 1-4 in the DHU stem; X position 1-5 in the anticodon stem; X position 1-5 in the TΨC stem; ant, anticodon; d, discriminator nucleotide
A compensatory base change implies the substitution of a nucleotide with a different base that does not disrupt the pairing in the stem.
Only trnS1s different from plesiomorphic condition trnS1GCT, are labelled with their anticodon i.e. trnS1ACT and trnS1TCT.

Reference

Montelli, S., Peruffo, A., Patarnello, T., Cozzi, B., Negrisolo, E. Back to water: signature of adaptive evolution in cetacean mitochondrial tRNAs. *PLoS ONE* **2016**, 11, e0158129.

Alignment S16

Multiple alignment of *trnF* sequences in Hesperiidae

```
                                                   10       20        30        40        50        60        70
                                                    |        |         |         |         |         |         |
                                               1234567 1234      4321 12345 ant 54321  1234     4321 7654321d
Hasora anura KR189008 trnF                     ATTTAAATAGCTTAAAAATTAGAGTATAATATTGAAGATATTAGGGTGATTGA---TAAAATCTTTAAATA   Coliadinae
Hasora vitta KR076553 trnF                     ATTTAAATAGCTTAA---ATAGAGTGTAATATTGAAGATATTAAGGTGATTGT----AAAATCTTTAAATA
Hasora badra MK238674 trnF                     ATTTAAATAGCTTA--AAATAGAGTATAATATTGAAGATATTAGGGTGATTG----TCAAATCTTTAAATA
Hasora chromus MN182753 trnF                   ATTTAAATAGCTTAAAAATTAGAGTATAATATTGAAGATATTAGGGTGATTT----TATAATCTTTAAATA
Burara striata KY524446 trnF                   ATTTAAATAGCTTAA---AAAGAGTATAATATTGAAGATATTAGGGTGATTAA---TTAAATCTTTAGATA
Choaspes benjaminii KJ629164 trnF              ATTTAAATAGCTTAT-ATAAAGAGTATAATATTGAAGATATTAGGGTGATTAA---ATTAATCTTTAAATA
Euschemon rafflesia KY513288 trnF              ATTTAAATAGCTTAA---AAAGAGTATAATATTGAAGATATTATGGTGATTAA--AAAAAATCTTTAAATA   Euschemoninae
Achalarus lyciades CM009487 trnF               ATTTAAATAGCTTAT---AAAGAGTATAATATTGAAGATATTAGGGTGATTAT---TTTAATCTTTAAATA   Eudaminae
Lobocla bifasciatus KJ629166 trnF              ATTTAAATAGCTTAA--AAAAGAGTATAATATTGAAGATATTAGGGTGATTAT----ATAATCTTTAAATA
Daimio tethys KJ629165 trnF                    ATTTGAATAGCTTAA-AATTAGAGCATAATATTGAAGATATTAAGGTGATTAT----TAAATCTTTAAATA   Tagiadinae
Tagiades vajuna KX865091 trnF                  ATTTAAATAGCTTAAATTTAGAGTTTAATATTGAAGATATTAAGGTGATTAA----AAAATCTTTAAATA
Abraximorpha davidii MT371044 trnF             ATTTAAATAGCTTAAAATTAAGAGTTTAATATTGAAGATATTAAGGTGATTG---TAAAAATCTTTAAATA
Celaenorrhinus maculosa KF543077 trnF          ATTTAAATAGCTTAA--TAAAGAGTATGATATTGAAGATATTAGGGTGATTTT---TTTAATCTTTAAATA
Erynnis montanus MN012984 trnF                 ATTTAAATAGCTTA---AAAAGAGTATAATATTGAAGATATTAAGGTAATTA--TTTATAATTTTTAAATA   Pyrginae
Erynnis tages LR990102 trnF                    ATTTAAATAGCTTA---AAAAGAGTATAATATTGAAGATATTAGGGTGATTA--TTTATAATTTTTAAATA
Pyrgus maculatus KP689265 trnF                 ATTTAAATAGCTTAA---AAAGAGTATAATATTGAAGATATTAGGGTAATTAT--TTATAATTTTTAAATA
Pyrgus maculatus MN013011 trnF                 ATTTAAATAGCTTA---AAAAGAGTATAATATTGAAGATATTAGGGTAATTA--TTTATAATTTTTAAATA
Pyrgus malvae BK013352 trnF                    ATTTAAATAGCTTA---AAAAGAGTATAATATTGAAGATATTAGGGTAATTA--TTTATAATTTTTAAATA
Leptalina unicolor MK265705 trnF               ATTTAAATAGCTCA--ATTAAGAGTTTAATATTGAAGATATTAAGGTGATTT----TATAATCTTTAAATA   Heteropterinae
Heteropterus morpheus KF881050 trnF            ATTTAAATAGCTTAA--AATAGAGTGTAATATTGAAGATATTAAGGTGATTTT-----TAATCTTTAAATA
Carterocephalus alcina MN012974 trnF           ATTTAAATAGCTTA-ATTTAAGAGTATAATATTGAAGATATTAGGGTGATTA---TTAAAATCTTTAAATA
Carterocephalus silvicola KJ629163 trnF        ATTTAAATAGCTTAA-TTTAAGAGTATAATATTGAAGATATTAGGGTGATTAT---TTGAATCTTTAGATA
Rachelia extrusus MN919192 trnF                ATTTAAATAGCTTAA-TTTAAGAGTGTAATATTGAAGATATTAAGGTGATAT---TTATAGTCTTTGAATA   Trapezitinae
Apostictopterus fuliginosus MH985707 trnF      ATTTAAATAGCTTAA---AAAGAGTGTAATATTGAAGATATTATGGTGATATA---TTATATCTTTAAATA   Barcinae
Barca bicolor MH985708 trnF                    ATTTAAATAGCTTAA---AAAGAGTACAATATTGAAGATATTGGGGTGATTGT----TAAATCTTTAAATA
Malaza empyreus MN919190 trnF                  ATTTAAATAGCTTAAATTAAAGAGTATAATATTGAAGATATTAGGGTGATTA---TTTAAATCTTTAAATA   Malazinae
Malaza fastuosus MK301537 trnF                 ATTTAAATAGCTTAATTAGAAGAGTATAATATTGAAGATATTGAGGTGATTT----TTAAATCTTTAAATA
Malaza carmides MN919191 trnF                  ATTTAAATAGCTTA---AAAAGAGTATAATATTGAAGATATTAGGGTGATTA-----TTAATCTTTAGATA
Ampittia dioscorides KM102732_trnF             ATTTAAATAGCTTAAATAATAGAGTATGATATTGAAGATATTATGGTGATTTT---GATAATCTTTAAATA   Hesperiinae
Ampittia subvittatus MN013003 trnFa            ATTTAAATAGCTTAAAATTAAGAGTTTAATATTGAAGATATTAAGGTGATTAAA--AAAAATCTTTAAATA
Ampittia subvittatus MN013003 trnFb            ATTTAAATAGCTTAAAATTAAAAGTTTAATATTGAAAATATTAAGGTGATTA-----AAAATCTTTAAATA
Ochlodes sylvanus FR990152 trnF                ATTTAAATAGCTTA-ATTAAAGAGTTTAATATTGAAGATATTAAGGTGATTG----AAAAATCTTTAAATA
Ochlodes thibetana MN013004 trnF               ATTTAAGTAGCTTA-AGGAAAGAGTATAATATTGAAGATATTAAGGTGATTT---ATGAAATCTTTAAATA
Hesperia comma FR990041 trnF                   ATTTAAATAGCTTAAATTTAAGAGTATAATATTGAAGATATTAAGGTGATTA----CTAAGTCTTTAAATA
Polytremis jigongi KP765762 trnF               ATTTAAATAGCTTAA--TAAAGAGTATAATATTGAAGATATTAAGGTGATTGT----ATTATCTTTAAATA
Polytremis nascens KM981865 trnF               ATTTAAATAGCTTAA--TAAAGAGTATAATATTGAAGATATTAAGGTGATTGT----ATTATCTTTAAATA
Ochlodes venatus HM243593 trnF                 ATTTAAATAGCTTAA-ATTTAGAGTTTAATATTGAAGATATTAAGGTGATAAA----ATTATCTTTAAATA
Lerema accius KT598278 trnF                    ATTTAAATAGCTTAA-AAGAAGAGTATAATATTGAAGATATTAAGGTGATTGAAAAAAAAATCTTTAAATA
Parnara guttata JX101619 trnF                  ATTTAAATAGCTTAA-ATAAAGAGTGTAATATTGAAGATATTAAGGTGATTTT-----TAATCTTTAGATA
Pelopidas mathias MW264491 trnF                ATTTAAATAGCTTAA-ATTGAGAGTGTAATATTGAAGATATTAAGGTAATTT----TATAATTTTTAAATA
Erionota torus MW586888 trnF                   ATTTAAATAGCTTAAATAAAAGAGTTTAATATTGAAGATATTAAGGTGATTA----AAGAATCTTTAAATA
Notocrypta curvifascia MH763665 trnF           ATTTAAATAGCTTA---AAAAGAGTATAATATTGAAGATATTAAGGTGATTA---ATAAAATCTTTAAATA
Potanthus flavus KJ629167 trnF                 ATTTAAATAGCTTAA-TTAAAGAGTATAATATTGAAGATATTGGGGTGATTGT----ATAATCTTTAAATA
Astictopterus jama MH763663 trnF               ATTTAAATAGCTTAATATTAAGAGCATGATATTGAAGATATTAAGGTAATTG---TAAAATTTTTAAATA
Isoteinon lamprospilus MH763664 trnF           ATTTAAATAGCTTAAAATTAAGAGTATAATATTGAAGATATTAGGGTGATTA-----AGAATCTTTAGATA
Megathymus beulahae KY630505 trnF              ATTTAAATAGCTTAA-GATAAGAGTTTAATATTGAAGATATTAAGGTGATTGT----AAAATCTTTAAATA
Megathymus yuccae KY630500 trnF                ATTTAAATAGCTTAA--TTAAGAGTTTAATATTGAAGATATTAAGGTGATTGA----AAAATCTTTAAATA
Megathymus ursus violae KY630502 trnF          ATTTAAATAGCTTAA-ATAAAGAGTTTAATATTGAAGATATTAAGGTGATTTA----AAAATCTTTAAATA
Megathymus cofaqui cofaqui KY630503 trnF       ATTTAAATAGCTTAA-ATTAAGAGTTTAATATTGAAGATATTAAGGTGATTG-----TAAATCTTTAGATA
Megathymus streckeri KY630501 trnF             ATTTAAATAGCTTAA-ATTAAGAGTTTAATATTGAAGATATTAAGGTGATTAA----AAAATCTTTAAATA
Agathymus mariae KY630504 trnF                 ATTTAAATAGCTTAA---ATAGAGTTTAATATTGAAGATATTAAGGTGATTAA----TTGATCTTTAAATA
                                               1234567 1234      4321 12345 ant 54321  1234     4321 7654321d
                                               0000000 1111      2222 22233 333 34444  4455     6666666666677
                                               1234567 0123      3456 78901 456 90123  8901     0123456789011
```

Pairwise alignment of trnFa and trnFb in *Ampittia subvittatus*

```
Ampittia subvittatus MN013003 trnFa    ATTTAAATAGCTTAAAATTAAGAGTTTAATATTGAAGATATTAAGGTGATTAAAAAAATCTTTAAATA [69 nucleotides]
Ampittia subvittatus MN013003 trnFb    ATTTAAATAGCTTAAAATTAAAAGTTTAATATTGAAAATATTAAGGTGATTA---AAAATCTTTAAATA [66 nucleotides]
                                       1234567 1234      4321 12345 ant 54321  1234    4321 7654321d
```

Alignment length: 69; identity: 64 (92.75%); different: 5 (7.25%).

Legend

N, the background base for the position.
N, half-compensatory base change in the stem pair (e.g. T - G vs C - G; A-T vs G-T).
N, N half-compensatory base change in the stem pair exhibiting a mismatch (e.g. T-A vs A-A).
N, N, fully compensatory base change in the stem pair exhibiting a mismatch (e.g. C-G vs T-T).
N, N, type I fully compensatory base change in the stem pair (i.e. purine - pyrimidine vs purine - pyrimidine, e.g. G - C vs A - T).
N, N, type II fully compensatory base change in the stem pair (i.e. purine - pyrimidine vs pyrimidine - purine, e.g. A - T vs T - A).
Different colors are used to better differentiate the changes.
N, a mismatch in the in the stem pair; N, a mismatch in a pair not always fully consistent (e.g. 5 5).
N, substitution pattern not modelled.
*, position in which a mismatch is prominent.
N, N, molecular signature for a taxon.
X, position 1-7 in the acceptor stem; X position 1-4 in the DHU stem; X position 1-5 in the anticodon stem; X position 1-5 in the TΨC stem; ant, anticodon; d, discriminator nucleotide
A compensatory base change implies the substitution of a nucleotide with a different base that does not disrupt the pairing in the stem.
Only trnS1s different from plesiomorphic condition trnS1[GCT], are labelled with their anticodon i.e. trnS1[ACT] and trnS1[TCT].

Reference

Montelli, S., Peruffo, A., Patarnello, T., Cozzi, B., Negrisolo, E. Back to water: signature of adaptive evolution in cetacean mitochondrial tRNAs. *PLoS ONE* **2016**, 11, e0158129.

Alignment S17

The intergenic spacers: **ISP *trnQa*-*trnQb*, ISP *trnQb*-*trnQc* and ISP *trnQc*-*trnQd***

Bhutanitis_thaidina MN012973 ISP ***trnQa*-*trnQb*** (segments: A, B, C, D)
ATTACCCTATTATATTGAATTTATATTTCAAAAAAAAAAAATCCCCCTGATTTGATTTTTTAATATTAATTCAAATAAAATATTTTTTTACCCTTTTTTTTTAAAAAAATTGCCTG
ATCAAAAGGATTATTTTAATAAATAAAAAATGTAATATAACTACCTTTA

Pairwise alignments of portions of ISP *trnQa*-*trnQb* with ISP *trnQd*-nad2, CoRe 3'-end, *trnM an trnI*

Bhutanitis thaidina **MN012973 ISP *trnQa*-*trnQb***	(**segment A**)	ATTACCCTATTATATTGAATTTATATTTCAAAAAAAAAAAATCCCCCT-------
Bhutanitis thaidina **MN012973 ISP *trnQd*-nad2**		ATTACCCTATTATATTGAATTTATATTTCAAAAAAAAAAA-TCCCCCTATTTTAA

Bhutanitis thaidina **MN012973 ISP *trnQa*-*trnQb***	(**segment B**)	GA-TTTGATTT-TTTAATATTAAT--TCAA-A-TAAAATATTTTTTTA
Bhutanitis thaidina **MN012973 CoRe (3' end)**		GAATACGATTTATTTAATATTAATATTTAATATTAAAAAAATTCTTTA

Bhutanitis thaidina **MN012973** ISP ***trnQa*-*trnQb***	(**segment C**)	---CCCTTTTTTTTTAAA
Bhutanitis thaidina **MN012973 *trnM***		TTAAAAATAAGCTAAATTTAAGCTTTTGGGTTCATACCCCAAATATAAAGGAAACCCCTTTTTTTTTAAA

Bhutanitis thaidina **MN012973** ISP ***trnQa*-*trnQb***	(**segment D**)	-A-AAATTGCCTGATCAAAAGGATTATTTTAATA-AATAAAAAATGTAATATAACTACCTTTA---
Bhutanitis thaidina **MN012973 *trnI***		AATAAAGTGCCTGATCAAAAGGATTATTTTGATAGAATAAAAAATGTAATATAATTACCTTTATTA

Pairwise alignment of ISPs trnQ-nad2 in species of *Bhutanitis*

Bhutanitis thaidina **MN012973 ISP *trnQd*-nad2**	ATTACCCTATTATATTGAATTTATATTTCAAAAAAAAAAATCCCCCTATTTTAA
Bhutanitis mansfieldi **LT999973 ISP trnQ-nad2**	AT-ATTTTATTATATTGAATTTATATTTCAAAAAAAAAAATCCCCCTATTTTAA
